# Seizure pathways change on circadian and slower timescales in individual patients with focal epilepsy

**DOI:** 10.1073/pnas.1922084117

**Published:** 2020-05-04

**Authors:** Gabrielle M. Schroeder, Beate Diehl, Fahmida A. Chowdhury, John S. Duncan, Jane de Tisi, Andrew J. Trevelyan, Rob Forsyth, Andrew Jackson, Peter N. Taylor, Yujiang Wang

**Affiliations:** ^a^Interdisciplinary Computing and Complex BioSystems Group, School of Computing Science, Newcastle University, Newcastle upon Tyne, NE4 5TG, United Kingdom;; ^b^UCL Queen Square Institute of Neurology, University College London, London, WC1N 3BG, United Kingdom;; ^c^Faculty of Medical Sciences, Newcastle University, Newcastle upon Tyne, NE2 4HH, United Kingdom

**Keywords:** focal epilepsy, seizure dynamics, functional connectivity, within-patient variability, intracranial EEG

## Abstract

Personalized medicine requires that treatments adapt to not only the patient but also changing factors within each individual. Although epilepsy is a dynamic disorder characterized by pathological fluctuations in brain state, surprisingly little is known about whether and how seizures vary in the same patient. We quantitatively compared within-patient seizure network evolutions using intracranial electroencephalographic (iEEG) recordings of over 500 seizures from 31 patients with focal epilepsy (mean 16.5 seizures per patient). In all patients, we found variability in seizure paths through the space of possible network dynamics. Seizures with similar pathways tended to occur closer together in time, and a simple model suggested that seizure pathways change on circadian and/or slower timescales in the majority of patients. These temporal relationships occurred independent of whether the patient underwent antiepileptic medication reduction. Our results suggest that various modulatory processes, operating at different timescales, shape within-patient seizure evolutions, leading to variable seizure pathways that may require tailored treatment approaches.

## Significance

Epilepsy is an episodic disease characterized by brief periods of abnormal brain activity, known as seizures, that often have clinical correlates. In many patients, seizures preferentially happen during certain stages of daily and multiday cycles. However, it is unclear whether and how seizures themselves change over time, even though such variability may have clinical implications. To address this knowledge gap, we quantitatively analyze the nature of within-patient variability in seizure network evolutions. Contrary to common expectations, we find seizure variability throughout our cohort. Moreover, we demonstrate that seizures do not change randomly; instead, they also appear to fluctuate over daily and slower timescales. Ultimately, we may improve treatments by tailoring interventions to the full repertoire of seizures in each patient.

Focal epilepsy is characterized by spontaneous, recurrent seizures that arise from localized cortical sites (1). An unresolved question is how much seizures themselves can vary in individual patients. Past studies suggest that seizures within a single patient share common features (2–6) and evolve through a similar sequence (7), or characteristic pathway (8), of spatiotemporal neural dynamics. However, there is also evidence that various aspects of seizures can differ within the same patient. Clinically, some patients have multiple seizure onset sites that each produce their own characteristic seizure dynamics (9), and long-term electroencephalographic (EEG) recordings suggest that a subset of patients have multiple types of seizure evolutions (8, 10–12). Ictal onset patterns (13, 14), the extent of seizure spread (15–17), and seizure recruitment patterns (18) can also differ in the same patient. This variability may arise from fluctuations in the underlying brain state (17, 19–23), suggesting that background neural activity affects not only seizure likelihood (20, 24) but also seizure evolution. Crucially, a given treatment may only address a subset of a patient’s seizures: for example, a single neurostimulation protocol may not control the complete repertoire of seizures (19), and a single prediction algorithm may fail to forecast all seizures (10, 12, 25). Consequently, seizure variability has important implications for clinical management in these patients.

To design optimal and comprehensive treatments, we therefore need to understand the prevalence and characteristics of within-patient seizure variability. Do seizure pathways vary in all patients? How are different seizure pathways distributed in time? To answer these questions, we must objectively quantify the similarity of seizure pathways. This task is challenging due to the complexity of seizure dynamics: a variety of spatiotemporal features change independently during seizure evolution. Although some studies have quantitatively compared within-patient seizures (26–31), the current gold standard remains visual inspection of ictal EEG by trained clinicians. This latter approach is time-consuming and subjective and can miss important features, including functional network interactions, that are difficult to detect visually.

Such functional network dynamics, also known as functional connectivity patterns, describe relationships between the activity recorded by different EEG channels. Temporal changes in network dynamics play important roles in seizure initiation, propagation, and termination (2, 23, 32–41), in part due to dynamic changes in the connectivity of the seizure onset zone (7, 42–44). Past work suggests that in some patients, the brain consistently progresses through a specific sequence of finite network states during seizures; however, other patients had unexplained variability in their seizure network evolutions (7). To fully understand how functional interactions support ictal processes, we must also understand if and how multiple seizure pathways, representing different ictal network evolutions, can coexist in an individual patient. Such diversity would reveal that the same neural regions can variably interact to produce a variety of pathological dynamics.

In this paper, we therefore focus on quantifying and characterizing within-patient variability in seizure network evolutions; however, our approach can be adapted to compare the temporal evolutions of any ictal feature of interest. We first visualized and compared the within-patient seizure network evolutions of human patients with focal epilepsy (recorded for 43 to 382 h). In each patient, we analyzed all seizures with clear electrographic correlates (i.e., both subclinical and clinical seizures). In total, we quantitatively analyzed 511 seizures (average 16.5 seizures per patient), allowing us to characterize the nature of within-patient variability in these dynamics. In each patient, we found variability in seizure network evolution, revealing that within-patient seizures are not well represented by a single characteristic pathway through network space. However, seizures can share parts or all of the same pathway, with recurring dynamical elements across seizures. Furthermore, we explored how seizure pathways change over different timescales, providing insights into the temporal changes of within-patient seizures. Our analysis revealed that seizures change on circadian and/or slower timescales in each patient, suggesting that different modulatory processes shape seizure pathways.

## Results

We analyzed seizure evolution in 31 human patients (511 seizures total, mean 16.5 seizures per patient) with focal epilepsy who underwent continuous intracranial electroencephalographic (iEEG) recordings as part of presurgical evaluation. Patient details are provided in *SI Appendix*, Text S1. In this section, we first demonstrate how we visualized seizure evolution through network space and quantified the dissimilarity of these seizure pathways. Importantly, our analysis captured differences in network interactions during seizures, which did not necessarily correspond to anatomical differences in the location and spread of seizure activity. We then describe the amount, form, and temporal patterns of within-patient seizure variability. Finally, we hypothesize how underlying processes occurring on different timescales could drive the observed changes in seizure pathways. We provide a visual guide to our approaches, along with the goals of each analysis, in *SI Appendix*, Text S2.

### Visualizing and Quantifying Variability in Within-Patient Seizure Pathways.

**Fig. 1.**
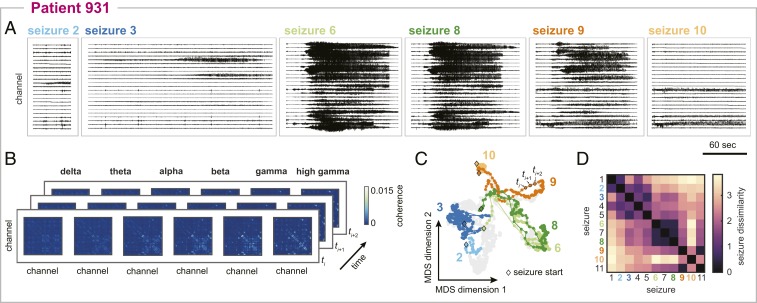
Visualizing and comparing seizure pathways through network space in an example patient, patient 931. (*A*) Intracranial EEG traces of a subset of the patient’s seizures. For visual clarity, only a representative subset of the recording channels are shown. (*B*) Functional connectivity of three example seizure time windows. Functional connectivity was defined as band-averaged coherence in each of six different frequency bands. Each matrix was normalized so that the upper triangular elements summed to 1. Self-connections are not shown. (*C*) Projection of all seizure time windows into a 2D space using MDS, allowing visualization of seizure pathways through network space. Each point corresponds to a seizure time window, and time windows with more similar network dynamics are placed closer together in the projection. Consecutive time windows in the same seizure are connected to visualize seizure pathways. The time windows and pathways of the six seizures shown in *A* have been highlighted using the corresponding colors, and the time windows of the remaining seizures are shown in gray for reference. The first time windows of the selected seizures are each marked with a black diamond. (*D*) Seizure dissimilarity matrix of all of the patient’s seizures, which quantifies the difference in the network evolutions of each pair of seizures. A low dissimilarity indicates that the two seizures have similar pathways through network space.

Our first goal was to objectively compare within-patient seizure network evolution. For each patient, we extracted the seizure iEEGs (Fig. 1*A*) and computed the sliding-window functional connectivity, defined as band-averaged coherence in six frequency bands (Fig. 1*B*). Thus, each seizure time window was described by a set of six connectivity matrices that captured interactions between iEEG channels in each frequency band. We additionally normalized the magnitude of each connectivity matrix to focus on the evolving patterns of network interactions, rather than gross changes in the global level of coherence. The set of all possible connectivity patterns created a high-dimensional space, in which each location corresponded to a specific network configuration. As such, each time window could be represented by a high-dimensional data point, and the evolution of a seizure’s network dynamics formed a pathway in this high-dimensional connectivity space. By transforming seizures in this manner, we framed our comparison of seizures as a comparison of seizure pathways (or trajectories) through the high-dimensional network space.

Due to the high dimensionality of this network space, it was infeasible to directly visualize seizure pathways. However, seizure pathways could be approximated in a two-dimensional (2D) projection using multidimensional scaling (MDS), a dimensionality reduction technique that attempts to maintain the distances between high-dimensional data points in the lower-dimensional space (Fig. 1*C*). This technique has been previously used to visualize ictal and interictal network dynamics (43). In our case, MDS placed seizure time windows in a 2D projection based on the similarity of their network configurations; each time window was represented by a single point, and points corresponding to time windows with more similar network dynamics were placed closer together. While imperfect, this approximation of the network space nonetheless provided an intuitive visualization for comparing seizure pathways in the same patient. For example, in patient 931, the projection demonstrated that two seizures may follow approximately the same pathway (seizures 6 and 8), part of the same pathway (seizures 8 and 9), or completely distinct pathways (seizures 2 and 10) through the network space, in agreement with visual impressions of the EEG.

To quantify these visual observations, we developed a seizure dissimilarity measure that provided a distance between two seizures based on their pathways through network space. Importantly, our approach recognized similarities in seizure pathways, even if the seizures evolved at different rates, by first applying dynamic time warping (45) to each pair of seizure functional connectivity time courses (*SI Appendix*, Text S3). Dynamic time warping nonlinearly stretches each time series such that similar points are aligned, thus minimizing the total distance between the two time series. We then defined the dissimilarity between two seizures as the average difference between the seizure pathways across all warped time points. The seizure dissimilarity matrix then summarized the dissimilarity between all pairs of seizure pathways in the same patient (Fig. 1*D*). In patient 931, seizures with similar pathways therefore had a low dissimilarity (e.g., seizures 6 and 8, dissimilarity 0.49); seizures with distinct, distant pathways had high dissimilarity (e.g., seizures 2 and 10, dissimilarity 3.21); and seizures with partially overlapping pathways had an intermediate level of dissimilarity (e.g., seizures 8 and 9, dissimilarity 1.75). Again, our measure of seizure dissimilarity agreed with intuitive comparisons of seizures based on visually assessing the iEEG (Fig. 1*A*) and MDS projections of the seizure pathways (Fig. 1*C*).

It is important to note that both seizure dissimilarity matrices and MDS projections were patient-specific: due to different electrode implantations, we could not compare seizures across patients using these network features. However, because we normalized the magnitude of the functional connectivity, we could compare seizure dissimilarity values across patients, even if the patients had different numbers of recording electrodes. In the remainder of the paper, we will focus on the across patients results, while using patient 931’s seizures as examples. The seizure variability analysis of all patients is available on Zenodo (46) and summarized in *SI Appendix*, Text S4.

### Seizure Variability Is a Common Feature in All Patients.

**Fig. 2.**
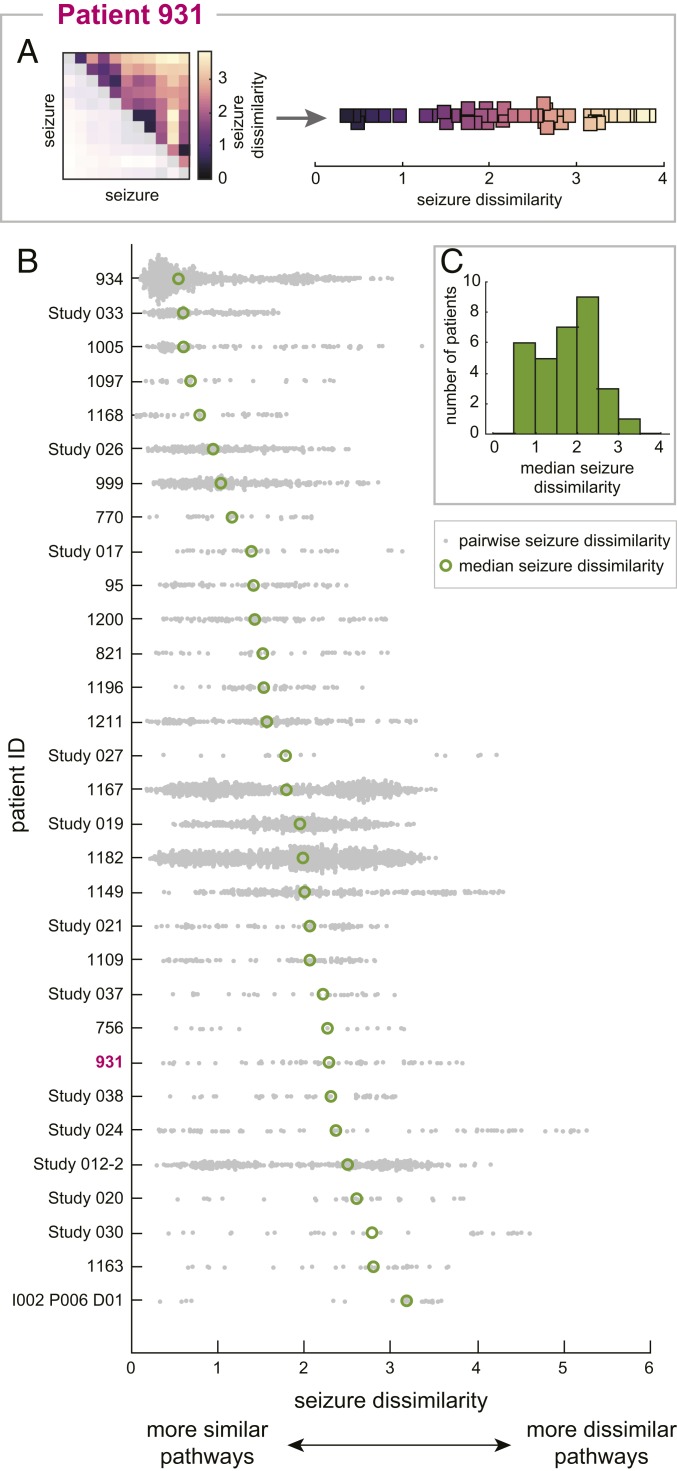
Variability in seizure pathways is common in all patients. (*A*) The seizure dissimilarity matrix of patient 931 (*Left*) was converted into a distribution of seizure dissimilarities (*Right*) to reveal the amount and form of seizure variability in this patient. Each point in the distribution corresponds to the dissimilarity of a pair of seizures (i.e., one of the squares in the seizure dissimilarity matrix). Because the matrix is symmetric, only the upper triangular entries are plotted in the distribution. (*B*) Distributions of seizure dissimilarities in each patient. Patients are sorted from lowest median seizure dissimilarity (patient 934) to highest median seizure dissimilarity (patient I002 P006 D01). Each gray point corresponds to the dissimilarity of a pair of seizures. The median dissimilarity of each distribution is marked by a green circle. (*C*) Histogram of the median seizure dissimilarities of all patients.

Using our measure of seizure dissimilarity, we compared seizure pathways through network space in each patient. We first determined if seizure variability was present in all patients by visualizing the seizure dissimilarity matrix of each patient as a distribution of seizure dissimilarities (see Fig. 2*A* for an example). Note that in these distributions, each point corresponds to the difference in network evolutions of a pair of seizures, rather than a feature of a single seizure. Fig. 2*B* shows the distribution of seizure dissimilarities in each patient, with patients sorted from lowest (patient 934) to highest (patient I002 P006 D01) median dissimilarity. Although the average level of variability differed between patients (Fig. 2*C*), it is apparent that all patients had variability in seizure network evolutions. Even in patients with more consistent seizures, such as patient 934, there were pairs of seizures with high dissimilarity, indicating dissimilar seizure pathways. Many patients, including patient 931, had varying levels of differences between pathways, with only a few pairs of similar seizures. In all patients, network differences across all frequency bands contributed to the observed seizure dissimilarities, revealing that variability in seizure network evolutions was not limited to a narrow frequency range within a given patient (*SI Appendix*, Text S5). Additionally, we found that in the majority of patients, the observed variability was best described as a spectrum of seizure pathways, rather than distinct groupings of different seizure pathways (*SI Appendix*, Text S6). Thus, in most patients, the full diversity of seizure pathways could not be captured by a few archetypal seizures.

We also explored if the observed seizure variability was related to the available clinical information for each patient. First, we found that within the same patient, seizures of the same clinical type (subclinical, focal, or secondarily generalized) tended to be more similar than seizures of different clinical types; however, there was still a large amount of variability within a given seizure type (*SI Appendix*, Text S7). Thus, seizure variability in our patients was not solely explained by the presence of different clinical seizure types. This finding was expected given that seizures of the same clinical type may have different features in the same patient (16, 47, 48). Additionally, we found no association between postsurgical seizure freedom and measures of seizure variability (*SI Appendix*, Text S8). Likewise, higher levels of seizure variability were not associated with a particular seizure onset site (*SI Appendix*, Text S8). These findings suggest that the level of seizure variability is not associated with certain patient pathologies or treatment outcomes; instead, other factors may be more crucial for determining the extent and form of the variability.

### Seizures with More Similar Pathways Tend to Occur Closer Together in Time.

**Fig. 3.**
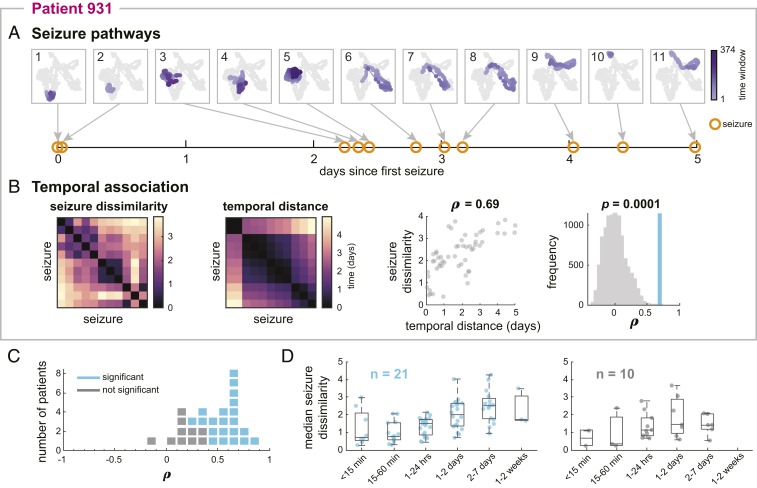
More similar seizures tend to occur closer together in time in most patients. (*A*) MDS projections of all of patient 931’s seizure pathways, numbered from first to last seizure. The pathway of each seizure is shown in purple, with earlier time windows in lighter purple. In each plot, the pathways of the remaining seizures are shown in gray for comparison. Below the pathways, the time of each seizure (orange circles) relative to the first seizure is shown. (*B*) From left to right, patient 931’s seizure dissimilarity matrix, temporal distance matrix, and comparison of seizure dissimilarities and temporal distances. The temporal distance matrix quantifies the amount of time between each pair of seizures, in days. Plotting the seizure dissimilarity vs. the corresponding temporal distance of each pair of seizures (scatterplot, third panel) reveals a positive Spearman’s correlation ρ between the two features. The significance (*P* value) of this correlation was determined using a permutation test (fourth panel); the observed correlation (blue) is shown against the distribution of correlations from permuted matrices (gray). (*C*) Dot plot showing the range of correlations between seizure dissimilarities and temporal distances across all patients. Each marker represents a patient (blue indicates significant correlation, and gray indicates not significant after false discovery rate correction). (*D*) Median seizure dissimilarities of pairs of seizures occurring within different time intervals (i.e., temporal distances) for patients with (*Left*; blue) and without (*Right*; gray) a significant correlation between seizure dissimilarities and temporal distances. Each point corresponds to the median dissimilarity of pairs of seizures occurring within the given time interval in a single patient. Some time intervals have fewer observations since some temporal distances were not observed in some patients. The boxplots indicate the minimum, lower quartile, median, upper quartile, and maximum of the distribution of median seizure dissimilarities, across the subset of patients, for that time interval.

Many time-varying factors, such as sleep (22, 24, 47, 49, 50) and hormones (51–54), are thought to influence seizure likelihood and dynamics. Additionally, during presurgical monitoring, antiepileptic medication is reduced in many patients, impacting brain dynamics (55). We therefore explored whether there is a temporal structure to how seizure pathways change over time in each patient. Fig. 3*A* shows the pathways of patient 931’s seizures, as well as the time that each seizure occurred relative to the patient’s first seizure. From this visualization, we see that the pathways gradually migrated through network space as the recording progressed, creating the observed spectrum of network evolutions. Moreover, looking at the seizure timings, we also see that seizures with similar pathways, such as seizures 6 to 8, tended to occur close together in time.

To quantify this temporal relationship, we defined a temporal distance matrix as the amount of time elapsed between each pair of the patient’s seizures (Fig. 3*B*). Patient 931’s seizure dissimilarity and temporal distance matrices have strikingly similar structures: groups of seizures with low dissimilarity tended to occur together in a relatively short time interval. In this patient, there was a strong and statistically significant positive correlation between these features (Spearman’s ρ = 0.69, *P* = 0.001, one-tailed Mantel test), indicating that seizures with more similar pathways tended to occur closer together in time.

Fig. 3*C* summarizes the relationship between seizure dissimilarities and temporal distances across all patients. In almost all patients, there was a positive Spearman’s correlation between seizure dissimilarities and temporal distances (range, −0.10 to 0.83; mean, 0.45). This association was significant in 21 patients (67.7%) after false discovery rate correction. In these patients, we also observed that the average level of dissimilarity tended to increase with the time between the two seizures (Fig. 3*D*). Interestingly, there was no association between whether antiepileptic medication was reduced and whether the correlation between seizure dissimilarities and temporal distances was significant (χ^2^ test, *P* = 0.96) (*SI Appendix*, Text S9). Therefore, although medication levels may affect seizure occurrence and dynamics (9, 16, 56, 57), medication changes alone could not explain the observed shifts in seizure pathways, suggesting that other factors also play a role in shaping seizure features.

### Seizure Pathways Change on Different Timescales.

The observed temporal associations of seizure dissimilarities reflected gradual changes in seizure network evolutions across the length of each recording. In other words, we observed relatively slow shifts in seizure pathways over the course of multiple days. However, we also hypothesized that seizure pathways may change on shorter timescales due to, for example, circadian rhythms. Such rhythms would create timescale-dependent relationships between seizures; in particular, there would be a positive correlation between seizure dissimilarities and temporal distances on shorter timescales, but this association would be destroyed over longer timescales.

**Fig. 4.**
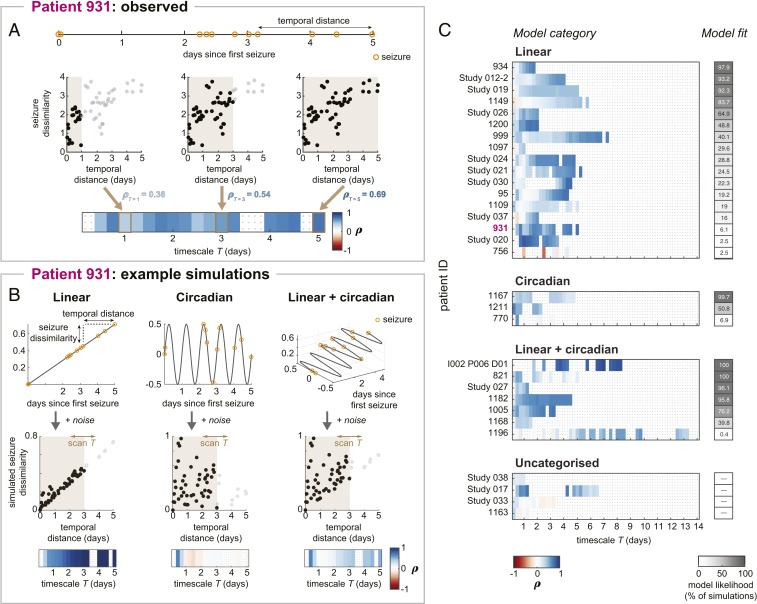
Temporal patterns of changes in seizure pathways. (*A*) Calculation of patient 931’s temporal correlation pattern. Temporal distances between seizures, which were derived from the patient’s seizure times (*Top*), were compared to seizure dissimilarities at different timescales (*Middle*). Example timescales *T* = 1 d, *T* = 3 d, and *T* = 5 d are shown (scatterplots; *Middle*). In each scatterplot, brown shading indicates the timescale, black points correspond to seizure pairs used to compute the correlation for that timescale, and gray points were pairs excluded from the correlation computation. At *T* = 5 d, all seizure pairs are included, producing the same temporal correlation as in Fig. 3*B*. Scanning the timescale produces a set of correlations, or temporal correlation pattern, shown in the heat map (*Bottom*). Gray dots in the heat map indicate insufficient information at that timescale, and these timescales are excluded from downstream analysis. (*B*) Seizure dissimilarities were modeled based on linear (*Left*), circadian (*Middle*), or a combination of linear + circadian (*Right*) changes in seizure pathways. The simulated changes in seizure pathways are shown as different functions of time (*Top*), with patient 931’s seizures marked in orange. Temporal distances and simulated seizure dissimilarities were compared across different timescales (scatterplots; *Middle*), yielding a temporal correlation pattern for each model (heat maps; *Bottom*). Example models and simulation results are shown here; the full set of tested models is provided in *SI Appendix*, Table S10. (*C*) Observed temporal correlation patterns of seizure pathways in each patient, categorized by the model that best reproduces these dynamics. The goodness of model fit was measured using model likelihood (gray heat map). The full details of each patient’s model are provided in *SI Appendix*, Fig. S10.2.

Therefore, to explore the possibility of different timescales of changes in seizure pathways, we scanned the correlation between seizure dissimilarities and temporal distances on different timescales *T* ranging from 6 h to the longest amount of time between a seizure pair (Fig. 4*A*). For example, for *T* = 3 d, we computed the correlation between seizure dissimilarities and temporal distances for all pairs of seizures that occurred within 3 d of each other. We refer to this set of correlations as a temporal correlation pattern of seizure pathways. Fig. 4*A* shows the temporal correlation pattern of patient 931’s seizures. As we determined earlier, there was a positive correlation between seizure dissimilarities and temporal distances when all seizures were included in the computation (*T* = 5 d) as a result of the observed gradual changes in seizure pathways. At shorter timescales, however, the temporal relationship fluctuated; for example, the correlation was relatively low at *T* = 1 and 2.5 d and higher at *T* = 0.75 and 2.5 d. These fluctuations were signs of additional, timescale-dependent changes in seizure pathways.

To investigate how these temporal correlation patterns arose, we modeled different patterns of seizure variability and the corresponding temporal correlation patterns (see *Materials and Methods* and *SI Appendix*, Text S10, for modeling details). For each patient, we then determined which pattern(s) of changes were most likely to reproduce the observed dynamics. In particular, we classified patients as having 1) linear changes in seizure pathways (Fig. 4*B*, *Left*), which corresponded to the slower, gradual shifts in seizure evolutions; 2) circadian changes (Fig. 4*B*, *Middle*), which represented dynamics modulated by daily rhythms; or 3) some combination of both the linear and circadian changes (example combination shown in Fig. 4*B*, *Right*).

Fig. 4*B* demonstrates how seizure dissimilarities were simulated using patient 931’s seizure times and example models from each of the above categories. In each model (Fig. 4*B*, *Top*), the *x* axis value of each seizure gives the seizure’s time, relative to the first seizure. These values are the same across all three models because they are the empirically observed seizure times of patient 931. Thus, the *x* axis distance between a pair of seizures measures the amount of time, or temporal distance, between them. Based on the seizure times, each model then predicted how seizure pathways would change from seizure to seizure; specifically, the distance between two seizures along the other dimension(s) corresponds to the simulated dissimilarity of each pair of seizures. Each model additionally included noisy dynamics that allowed for further, random fluctuations in seizure pathways and thus seizure dissimilarities (*SI Appendix*, Fig. S10.1).

From these temporal distances and simulated seizure dissimilarities (Fig. 4*B*, *Middle*), we then computed the corresponding temporal correlation patterns (Fig. 4*B*, *Bottom*) using the same process shown in Fig. 4*A*. A linear change in seizure pathways produced a positive temporal relationship that was stronger at longer timescales. Meanwhile, a circadian model only produced strong, positive temporal correlations at timescales shorter than 1 d. Finally, a combination of the linear and circadian factors created both the short-term temporal relationships and a positive temporal correlation at the longer timescales. Note that there were also some additional fluctuations in the temporal correlation patterns due to noisy changes in dynamics; these effects differed depending on the outcome of the noisy simulation. To fully explore these noisy effects, we therefore additionally varied the level of noise added to the models. The tested combinations of noisy, linear, and circadian contributions are provided in *SI Appendix*, Table S10. For each combination of these factors, we simulated temporal correlation patterns 1,000 times using different noise realizations to produce a series of possible temporal correlation patterns for each model.

Fig. 4*C* shows the type of model (linear, circadian, or linear + circadian) most likely to underlie the observed temporal correlation pattern of each patient. As a measure of model fit, we defined model likelihood as the percentage of model simulations that reproduced the patient’s observed temporal correlation pattern. Model likelihood ranges from 0 to 100%, and higher values reveal that the modeled changes in dynamics consistently produced temporal correlation patterns that were similar to the patient’s observed temporal correlation pattern. We additionally required the selected model to 1) outperform noisy simulations alone; 2) clearly distinguish between the linear and circadian models; and 3) in the case of the linear + circadian model, clearly outperform one of the simpler models. Using these criteria, 17 patients’ temporal correlation patterns were best explained by the linear model, 3 by the circadian model, and 7 by the linear + circadian model. Thus, most patients (77.4%) required a linear component to explain the observed changes in seizure pathways, while 32.3% of the patients were well matched by a model incorporating circadian changes in pathways. Notably, model likelihood tended to be higher for patients with higher number of seizures, reflecting greater model certainty in cases with larger sample sizes (*SI Appendix*, Fig. S10.4). These different classifications of seizure variability were not associated with surgical outcomes (*SI Appendix*, Text S8) or whether the patient’s medication was reduced during presurgical monitoring (*SI Appendix*, Text S9).

Four patients’ temporal correlation patterns could not be assigned to a model, either because the linear model and circadian model performed similarly (patient Study 038) or the best model did not outperform noise alone (patients Study 017, Study 033, and 1163). Additionally, in some patients (e.g., patients Study 020, 756, and 1196), only a small percentage of the simulations matched the observed temporal correlation patterns, indicating that reproducing the observed dynamics required specific patterns of noise. In these cases, other models may therefore provide a better explanation for the patient’s changes in seizure pathways. In particular, many of these patients had strong positive correlations at timescales longer than 1 d but less than the length of the recording, suggesting multiday fluctuations in seizure pathways.

## Discussion

We have quantitatively compared seizure network evolutions within individual human patients with focal epilepsy, revealing that seizure variability is a common feature across patients. We often observed pairs of seizures with relatively low dissimilarity due to their largely conserved pathways through the space of possible network dynamics, suggesting that seizure evolution is not purely random. However, we likewise found that a single dynamical pathway cannot comprehensively represent all of a patient’s seizure evolutions. Interestingly, seizure pathways changed over time in most patients, with more similar seizures tending to occur closer together in time. Our modeling results indicate that in most patients, a combination of circadian and/or slower changes in seizure pathways may underlie the observed variability, suggesting that factors operating on different timescales modulate within-patient seizure evolutions.

We investigated variability in seizure functional network evolution due to the importance of network interactions in ictal processes (2, 7, 23, 32–40, 42–44) and build on previous work by demonstrating within-patient variability in these pathological network dynamics. However, in future work, the framework we present could easily be adapted to compare other features that highlight different aspects of seizure dynamics. For example, a univariate feature that captures the amplitude and frequency of ictal discharges may be better suited for comparing the involvement of different channels, similar to how clinicians visually compare EEG traces. Data from other recording modalities, such as microelectrode arrays, could be analyzed to evaluate consistency in neuronal firing patterns between seizures (4, 5). Meanwhile, although we do not perform biophysical modeling of seizure dynamics in this work, other studies have used model inversion to hypothesize how the activities of different neuronal populations change during seizures (8, 58, 59). Comparing the parameter time courses of such models could reveal different patterns of changes in neural activity during a patient’s seizures. Finally, due to patient-specific recording layouts, we focused on comparing seizure pathways within individual patients. However, comparing seizures across patients, either using spatially independent features or common recording layouts, in future studies could uncover common classes of pathological dynamics (8, 60).

To quantify within-patient variability in seizure pathways, we developed a seizure dissimilarity measure that addresses the challenges of comparing diverse spatiotemporal patterns across seizures. A few previous studies have attempted to quantitatively compare seizure dynamics using either univariate (27, 28, 30, 31) or network (26, 29) features computed from scalp or intracranial EEG. These earlier dissimilarity measures were based on edit distance, which captures how many replacements, insertions, and deletions are required to transform one sequence into another. Importantly, unlike this previous method, our dynamic time warping approach recognizes that two seizures are equivalent if they follow the same pathway, even if they do so at different rates. Despite this difference, those past studies also reported both common and disparate dynamics across within-patient seizures; however, their analysis was limited to a small number of patients and/or seizures per patient. Our work provides insight into the prevalence and characteristics of seizure variability by analyzing over 500 seizures across 31 patients. Finally, we expand on previous work by using seizure dissimilarity to characterize temporal changes in seizure evolutions.

Previous work has found that within-patient seizures have similar dynamics (2–8), although variability may be introduced through different rates of evolution (4, 61) or early termination in the seizure pathway (6, 8). In our cohort, we observed that subsets of within-patient seizures follow approximately the same dynamical pathway through network space, and such similar groups of seizures likely underlie these past findings. However, we also found that the complete repertoire of within-patient seizure network evolutions was poorly characterized by a single, characteristic pathway. Notably, we also found that a patient with different seizure pathways did not necessarily have subsets of distinct pathways; instead, small variations between seizures often produced a spectrum of pathways. An intriguing possibility is that various decision points, existing on the framework of potential seizure pathways, produce a repertoire of seizure evolutions. Future studies are needed to map these potential seizure pathways and uncover the factors that determine how individual seizures evolve.

The crucial question is then how these different seizure pathways arise from the same neural substrate. In theory, a range of changes before or during the seizure can affect its network evolution. We hypothesize that spatiotemporal changes in the interictal neural state produce seizures with different characteristics. Past studies suggest that neural excitability (20, 57, 62), inhibition (61), and network interactions (23, 63) influence certain spatiotemporal seizure features, such as the rate and extent of seizure propagation. These changes in brain state may be driven by various factors, including sleep (22, 47, 49), hormones (51–54), and medication (55). If interictal dynamics indeed shape how seizures manifest, future research will need to determine how specific interictal features relate to seizure characteristics. One possibility is that elements of seizure networks are activated during interictal states (43); thus, seizures with different network features could be preceded by preictal periods with corresponding network structures. Researchers could also relate preictal/interictal networks to other seizure features, such as seizure propagation patterns, perhaps by investigating how the underlying structural connectome relates to functional networks (64) and mediates seizure spread (63). Importantly, the relationship between preictal network dynamics and seizure features could be limited to a specific frequency band (23), which could in turn suggest possible physiological mechanisms for the observed changes in seizure dynamics (65, 66). Additional aspects of interictal dynamics, such as the pattern of high-frequency oscillations (21) and band power changes (17), may also be linked to changes in seizure features. Overall, a better understanding of both functional and pathological fluctuations in interictal dynamics could suggest ways in which the background brain state alters seizure evolutions.

Toward this goal, prolonged recordings of patients with focal epilepsy may provide insight into how pathological brain dynamics change over time and influence seizure features. In particular, recent studies using such data have shown that the rates of epileptiform discharges and seizures fluctuate according to both circadian and patient-specific multidien (approximately weekly to monthly) cycles (50, 67). An intriguing possibility is that the same factors that rhythmically modulate seizure likelihood may also influence seizure evolution. Consistent with this hypothesis, we found that the majority of observed temporal patterns of seizure variability were well explained by models incorporating circadian and/or linear changes in seizure pathways. In particular, the linear component of the model may reflect gradual changes in pathways on slower timescales, ranging from weeks to months. These simple models provided an initial hypothesis for the observed patterns of changes in seizure evolutions. Some patients’ seizure patterns may be better explained by more complex models that capture different dynamics, such as multistability or multidien cycles. Ultimately, it is likely that various factors, with differential effects on seizure evolution, interact to produce the observed repertoire of seizure pathways. Analyzing within-patient seizure variability in long-term recordings could provide additional insight into such patterns of changes in seizure pathways.

Many of the patients in our study underwent antiepileptic medication reduction as part of presurgical monitoring, making it difficult to disentangle the effects of changing drug levels from other potential slow-varying modulators of seizure pathways. Changes in antiepileptic medication can impact neural excitability (68–70), and medication tapering increases seizure likelihood in most patients (16, 71); however, it is controversial whether it also affects seizure patterns (9, 16, 56, 71). In some cases, it appears that medication tapering reveals latent seizure pathways that are suppressed by medication (9) or allows existing pathways to further progress (e.g., the secondary generalization of typically focal seizures) (16). It is possible that the impact of medication reduction on seizure dynamics is drug-, patient-, and dose-dependent and may ultimately depend on how well the medication controls neuronal excitability (57). However, medication changes alone cannot account for the observed seizure variability in our cohort, as we observed temporal associations of seizure pathways in patients that did not undergo medication reduction. In future work, associating medication levels with differences in seizure pathways could help untangle the different factors shaping seizure dynamics.

Another confounding factor in our data is that the surgical implantation itself could artificially alter seizure dynamics. Using chronic recordings of epileptic canines, Ung et al. (72) found variability in seizure onset and interictal burst dynamics, with the most stable dynamics emerging approximately a few weeks after electrode implantation. In agreement with their work, we found that earlier seizure evolutions often recurred later in the recording, making it unlikely that gradual changes in the recording quality or an acute reaction to the surgery underlay the observed variability. Instead, Ung et al. (72) hypothesized that seizure variability results from transient, atypical dynamics as the brain recovers from surgery, with later dynamics representing a truer epileptic network. Other stressors, such as medication withdrawal, could similarly elicit abnormal dynamics. Nevertheless, a large number of our patients had good surgical outcomes, suggesting that their recorded seizures accurately represented their epileptic networks. Additionally, clinicians often note that patients have typical seizures during iEEG recordings, as compared to preimplantation reports, despite the effects of surgery and medication withdrawal (16). As such, the observed seizure dynamics in our cohort may be part of their usual repertoires of seizure evolutions, even if some dynamics are only elicited by strong stressors. Further analysis in chronic human recordings, such as the NeuroVista dataset (8, 12), is needed to determine whether and how seizure pathways vary in a more naturalistic setting.

Contrary to the expectation that high levels of seizure variability may worsen surgical outcomes, we found no association between these patient features. It may be that only some types of variability, such as multifocal (9) or secondarily generalized (73) seizures, impact the likelihood of seizure freedom following surgery. Importantly, variability in the seizure onset network state does not indicate that a patient has multifocal seizures, as different network configurations can be associated with the same apparent ictal onset zone. Additionally, variability in seizure pathways may not be inherently deleterious, as long as it is observed and accounted for when planning the surgical resection. Indeed, due to the short presurgical monitoring time and limited spatial coverage of the recording electrodes, some potential seizure pathways may not have been captured (11, 72), leading us to underestimate the level of variability in some patients.

Although the amount of seizure variability was not associated with postsurgical seizure freedom, it may have implications for clinical treatments. First, regardless of the source of the observed seizure variability, the different seizure dynamics observed during presurgical monitoring provide crucial information for guiding surgical resection. For example, recent studies suggest that seizure network properties can help identify epileptogenic tissue (7, 74, 75); however, we must determine if seizures with different network evolutions provide equivalent localization information. Seizure variability may also have implications for seizure prediction. In particular, in that same patient, seizures with different pathways may have distinct preictal signatures, making seizure prediction more difficult (10, 12). A successful seizure prediction algorithm would either need to recognize multiple signatures or find common features among the disparate preictal dynamics. Finally, neurostimulation offers a promising new approach for controlling seizures; however, in rodent models, the effectiveness of a given stimulation protocol depends on the preictal brain state (19). Thus, such interventions may need to recognize and adapt to the specific characteristics of each corresponding seizure evolution in order to control all seizures. Importantly, our cohort was limited to patients with medication refractory focal epilepsy who were candidates for surgical resection. The characteristics and clinical implications of seizure variability may be different in other patient cohorts.

More generally, our work adds to the growing literature on within-subject variability in brain dynamics and other physiological states (76) in both health and disease. In particular, there is increasing interest in developing improved, time-sensitive treatments that adapt to the patient’s changing state. Rather than delivering a continuous or regular therapy, such treatments would be modified and/or timed to improve their efficacy while also reducing treatment side effects. Treatment parameters may be tuned to biological rhythms (50, 67, 77–79) or respond directly to fluctuating conditions within each patient (80–82). To investigate temporal fluctuations within each patient and determine how treatments interact with these changes, researchers may draw inspiration from spatiotemporal analyses in other fields, such as ecology (83), genetics (84), and engineering (85, 86), as well as develop new techniques that address specific data-analytical challenges.

In summary, we have shown that there is within-patient variation in seizure network evolution in patients with focal epilepsy. Temporal changes in seizure evolution suggest that a combination of circadian and slow-varying factors shape these seizure pathways, perhaps by modulating the background brain state. Further research is needed to determine whether and how preictal dynamics influence seizure pathways. Uncovering these mechanisms could provide novel approaches for predicting and controlling seizures that are tailored to the complete repertoire of pathological neural dynamics in each patient.

## Materials and Methods

### Patient Selection and Data Acquisition.

This work was a retrospective study that analyzed seizures from 13 patients from the Mayo Clinic and the Hospital of the University of Pennsylvania [available on the IEEG Portal, https://www.ieeg.org/ (87, 88)] and 18 patients from the University College London Hospital (UCLH) who were diagnosed with refractory focal epilepsy and underwent presurgical monitoring. Patients were selected without reference to the cause or other characteristics of their pathology. All IEEG Portal patients gave consent to have their anonymized iEEG data publicly available on the International Epilepsy Electrophysiology Portal (https://www.ieeg.org/) (87, 88). For the UCLH patients, their iEEG was anonymized and exported, and the anonymized data were subsequently analyzed in this study under the approval of the Newcastle University Ethics Committee (reference number 6887/2018).

For each patient, the placement of the intracranial electrodes was determined by the clinical team, independent of this study. Ictal segments were identified and extracted for the analysis based on clinical seizure markings. To be included in the study, each patient was required to have had at least six seizures suitable for the analysis. This threshold was chosen to allow examination of seizure variability in a broad cohort of patients, while still ensuring that enough seizures were observed to draw conclusions about the characteristics of seizure variability in each patient. Seizures were excluded from the analysis if they did not have clear electrographic correlates (with clear onset and termination), if they were triggered by/occurred during cortical stimulation, if they had noisy segments, or if they had large missing segments. Periods of status epilepticus and continuous epileptiform discharges were also excluded. However, electrographic seizures without clinical correlates (i.e., subclinical seizures) were included in the analysis as they may have either similar or disparate dynamics (relative to clinical seizures) that convey clinically relevant information (89). Additional information about each patient and the analyzed seizures is shown in *SI Appendix*, Text S1.

### iEEG Preprocessing.

For each patient, if different seizures were recorded at multiple sampling frequencies, all of the recordings were first downsampled to the lowest sampling frequency. Noisy channels were then removed based on visual inspection. In the remaining channels, short sections of missing values were linearly interpolated. These sections of missing values were <0.05 s with the exception of one segment in seizure 2 of patient Study 020, which was 0.514 s. All channels were rereferenced to a common average reference. Each channel’s time series was then bandpass filtered from 1 to 150 Hz (fourth-order, zero-phase Butterworth filter). To remove line noise, the time series were additionally notch filtered (fourth-order, 2-Hz width, zero-phase Butterworth filter) at 60 and 120 Hz (IEEG Portal patients) or 50, 100, and 150 Hz (UCLH patients).

### Computing Functional Connectivity.

To compute the time-varying functional connectivity of each seizure, a 10-s sliding window, with 9-s overlap between consecutive windows, was applied to each preprocessed ictal time series. The same sliding window parameters have previously been used to estimate time-varying coherence in ictal iEEG data (90). For each window, the coherence between each pair of iEEG channels was computed in six different frequency bands (delta 1 to 4 Hz, theta 4 to 8 Hz, alpha 8 to 13 Hz, beta 13 to 30 Hz, gamma 30 to 80 Hz, and high gamma 80 to 150 Hz). The coherence in each frequency band was computed using band-averaged coherence, defined as

$$C_{i,j}\;=\;\frac{{\left|\sum_{f=f_{1}}^{f_{2}}P_{i,j}\left(f\right)\right|}^{2}}{\sum_{f=f_{1}}^{f_{2}}P_{i,i}\left(f\right)\sum_{f=f_{1}}^{f2}P_{j,j}\left(f\right)},$$

where *f*_1_ and *f*_2_ are the lower and upper bounds of the frequency band; *P*_*i,j*_(*f*) is the cross-spectrum density of channels *i* and *j*; and *P*_*i,i*_(*f*) and *P*_*j,j*_(*f*) are the autospectrum densities of channels *i* and *j*, respectively. In each window, channel autospectra and cross-spectra were calculated using Welch’s method (2-s sliding window with 1-s overlap). Note that band-averaged coherence is equivalent to coherence but filtered in the frequency domain to the frequency band of interest. As such, band-averaged coherence ranges from 0 to 1 and will be higher when the two signals have a consistent phase and amplitude relationship in the specified frequency band.

Thus, in a patient with *n* iEEG channels, the functional connectivity of each time window was described by six symmetric, nonnegative, *n* × *n* matrices, in which each entry (*i*,*j*) gives the coherence between channels *i* and *j* in the given frequency band. Each matrix was then written in vector form by rearranging the upper-triangular, off-diagonal elements into a single column vector of length (*n*^2^ – *n*)/2. Each vector was normalized so that the *L*1 norm (i.e., sum of all elements) was 1, thus ensuring that differences between connectivity vectors captured a change in connectivity pattern rather than gross changes in global levels of coherence. This normalization step also allowed the magnitude of seizure dissimilarities to be compared across patients with different numbers of electrodes. For each time window, the six connectivity vectors were then vertically concatenated together, forming a single column vector of length 6*(*n*^2^ – *n*)/2. Each patient’s ictal connectivity vectors were subsequently horizontally concatenated together to form a matrix *V* containing 6*(*n*^2^ – *n*)/2 features and *m* observations, where *m* is the total number of ictal windows across all seizures.

### Dimensionality Reduction and Visualization.

Small fluctuations in the functional connectivity due to noise would create a high baseline dissimilarity between seizures. Therefore, to reduce noise in the connectivity matrices, nonnegative matrix factorization (NMF) (91) was used to approximately factor each patient’s ictal time-varying connectivity matrix *V* into two nonnegative matrices, *W* and *H*, such that *V*∼*W* × *H* (details provided in *SI Appendix*, Text S11). The matrix *W* contained patient-specific basis vectors, each of which had 6*(*n*^2^ – *n*)/2 features that captured a pattern of connectivity across all channels and frequency bands. Each original ictal time window was summarized as an additive combination of these basis vectors, with the coefficients matrix *H* giving the contribution of each basis vector to each time window. These factorizations were patient-specific since the basis vector features depended on the iEEG electrode layout in each patient. The optimal number of basis vectors, *r*, was determined using stability NMF (84).

For each patient the selected factorization was then used to create V* = W × *H*, a lower-rank approximation of the original time-varying seizure functional connectivity (*SI Appendix*, Text S11). This return to the original feature space is necessary since NMF basis vectors are not orthogonal, and distances in NMF basis vector space are therefore not equivalent to distances in feature space. Each reconstructed connectivity vector was then renormalized to have an *L*1 norm of 1, ensuring that differences in reconstruction accuracy did not affect the distances between different ictal time points. To visualize the connectivity vectors of patient 931’s seizures in Fig. 1*C*, all time seizures windows were projected into a 2D embedding using MDS (specifically, Sammon mapping) based on their *L*1 (cityblock) distances in the high-dimensional reconstructed feature space.

### Computing Seizure Dissimilarities.

Following the NMF-based reconstruction of the seizure connectivity, the network evolution of each seizure was described by a multivariate time series with 6*(*n*^2^ – *n*)/2 features. To compare network evolutions across within-patient seizures, a seizure dissimilarity matrix was created for each patient. Each pair of seizure functional connectivity time series was first warped using dynamic time warping, which stretches each time series such that the total distance between the two time series is minimized (*SI Appendix*, Text S3). This step ensures that 1) similar network dynamics of the two seizures are aligned and 2) the warped seizures are the same length. We chose to minimize the *L*1 distance between each pair of seizures as this metric provides a better measure of distances in high-dimensional spaces (92).

Following dynamic time warping, the *L*1 distance between the pair of warped time series was computed, resulting in a vector of distances capturing the dissimilarity in the seizures’ network structures at each time point. The seizure dissimilarity between the two seizures was defined as the average distance across all warped time points. The seizure dissimilarity matrix contains the dissimilarities between all pairs of the patient’s seizures. Note that seizure dissimilarity is not a metric distance, because the triangle equality does not necessarily hold; however, it performs similarly to alternative metric distances of seizure dissimilarity (*SI Appendix*, Text S12).

### Comparison to Temporal Distances.

For each patient, we computed a temporal distance matrix containing the amount of time elapsed (measured in days) between the onset times of each pair of seizures. Spearman’s correlation was computed between the upper triangular elements of the seizure dissimilarity matrix and the temporal distance matrix of each patient. Since the distances in each matrix were not independent observations, the Mantel test (93) was used to determine the significance of each correlation. Briefly, the rows and columns of one matrix were randomly permuted 10,000 times. The correlation between the two sets of upper triangular elements was recomputed after each permutation, resulting in a distribution of correlation values that described the expected correlation if there were no relationship between seizure dissimilarities and temporal distances. The *P* value of the association was then defined as the proportion of permuted correlation that was greater than or equal to the observed correlation. To correct for multiple comparisons, the Benjamini–Hochberg false discovery rate (FDR) correction (94) was applied to the set of *P* values computed across all patients (31 total tests). The correlation was considered significant if the associated adjusted *P* value was less than 0.05.

### Computing Temporal Correlation Patterns.

To quantify how seizure dynamics change over different timescales in each patient, Spearman’s correlation between seizure dissimilarities and temporal distances was computed only for seizure pairs with temporal distances less than or equal to timescale *T*. *T* was scanned from 0.25 d up to the patient’s largest temporal distance in steps of 0.25 d. A timescale was excluded from the analysis if fewer than seven pairs of seizures occurred within the given timescale or if no new seizure pairs were added when the timescale was increased. The resulting set of correlations across various timescales was referred to as a temporal correlation pattern.

### Modeling Seizure Dissimilarities and Temporal Correlation Patterns.

To determine the underlying processes that could produce the observed temporal correlation patterns, changes in seizure dynamics were modeled using the functions

$f_{l}\left(t\right)=\;\frac{1}{7}t\;\left(a\;\mathrm{line}\;\mathrm{with}\;a\;\mathrm{slope}\;\mathrm{of}\;\mathrm{one}\;\mathrm{per}\;\mathrm{week}\right),$
$f_{c}\left(t\right)=\;\sin2\pi t\left(a\;\mathrm{sine}\;\mathrm{wave}\;\mathrm{with}\;a\;\mathrm{period}\;\mathrm{of}\;1\;\text{d}\right)\text{,}$
$f_{n}\left(t\right)\;∼\;N\left(0,1\right)\;\left(\mathrm{Gaussian}\;\mathrm{noise}\;\mathrm{with}\;a\;\mathrm{mean}\;\mathrm{of}\;\mathrm{zero}\;\mathrm{and}\;\mathrm{SD}\;\mathrm{of}\;1\right)\text{,}$

where *t* is time in days.

For each function, a simulated distance matrix *D* was then defined for the patients’ seizures, with

$$D\left(i,j\right)=\;\left|f\left(t_{i}\right)-f\left(t_{j}\right)\right|,$$

where *t*_*i*_ is the time of seizure *i*, *t*_*j*_ is the time of seizure *j*, and *f*(*t*) is the corresponding function. The dissimilarity of the two seizures was then defined as

$$Diss\left(i,j\right)=\;\sqrt{{\left[lD_{l}\left(i,j\right)\right]}^{2}+\;{\left[cD_{c}\left(i,j\right)\right]}^{2}+\;{\left[nD_{n}\left(i,j\right)\right]}^{2}},$$

where *l*, *c*, and *n* are scalar parameters controlling the relative contributions of the linear, circadian, and noise functions, respectively.

The relative contributions of the linear, circadian, and noise functions were scanned by varying the levels of *l*, *c*, and *n*. For each set of parameters, seizure dissimilarities were simulated 1,000 times using different noise realizations (and correspondingly changing the noise distance matrix, *D*_*n*_), and the resulting temporal correlation patterns were computed for each set of simulated dissimilarities. Note that because temporal correlation patterns only depend on the order of the dissimilarities, only the relative magnitudes of the parameters *l*, *c*, and *n* affected the modeling results. A model was termed a linear model if *c* = 0, a circadian model if *l* = 0, and a linear + circadian model if *l* > 0 and *c* > 0.

To determine if a patient’s seizure dynamics could be categorized as linear, circadian, or linear + circadian, the simulated temporal correlation patterns were compared to the patient’s observed temporal correlation pattern by computing the mean squared error (MSE) of each simulated pattern. Simulated temporal correlation patterns with MSE ≤ 0.02185 were defined as good matches to the observed dynamics. This threshold was chosen because it was the fifth percentile of the set of all MSEs, across all patients, and based on visual inspection of simulated temporal correlation patterns with different MSEs. The likelihood *L* of a given parameter set was then defined as the percentage of good matches produced by the 1,000 noisy simulations of seizure dissimilarities at those parameter values. For each class of model (linear, circadian, or linear + circadian), the model’s likelihood (*L*_*l*_, *L*_*c*_, or *L*_*l+c*_, respectively) was the highest likelihood among the model type’s parameter sets, and the best model was the model with the highest likelihood. *L*_*n*_ was also defined as the highest likelihood of the parameter sets without any linear or circadian contributions (*l* = 0, *c* = 0, *n* > 0).

This best model with likelihood *L*_*max*_ was then used to categorize the patient’s dynamics if it outperformed all competing models. Specifically, we required that 1) the best model clearly outperform noise alone (*L*_*max*_ ≥ 2*L*_*n*_); otherwise, the patient’s dynamics were classified as other/indeterminate; 2) the performance of the linear model and circadian model were clearly distinguishable (*L*_*l*_ ≥ 2*L*_*c*_ if the linear model was best; *L*_*c*_ ≥ 2*L*_*l*_ if the circadian model was best); otherwise, the patient’s dynamics were classified as other/indeterminate; and 3) if the best model was linear + circadian, it clearly outperform the two simpler models (*L*_*l+c*_ ≥ 2*L*_*l*_ and *L*_*l+c*_ ≥ 2*L*_*c*_); otherwise, the patient’s dynamics were classified as the simpler model (if one simpler model performed comparably by this criterion) or as other/indeterminate (if both simpler models performed comparably).

See *SI Appendix*, Text S10, for additional modeling details and the selected models for each patient.

### Code and Data Availability.

All data were analyzed using MATLAB version R2018b. To perform NMF, we used the Nonnegative Matrix Factorization Algorithms Toolbox, available at https://github.com/kimjingu/nonnegfac-matlab/, which implements the alternating nonnegative least squares with block principal pivoting algorithm (95, 96). For the remainder of the analysis, we used MATLAB implementations of standard algorithms (multidimensional scaling [Sammon mapping]: mdscale, criterion “Sammon”; dynamic time warping: dtw; hierarchical clustering: linkage; Torgerson’s multidimensional scaling: cmdscale; gap statistic: evalclusters; FDR correction: mafdr) or custom code. The iEEG time series of all IEEG Portal patients is available at https://www.ieeg.org/. The NMF factorizations of all analyzed seizure network evolutions, along with the code for producing the primary downstream results (seizure dissimilarity matrices, clustering, and temporal analysis) and figures, are published on Zenodo (46).

## Supplementary Material

Supplementary File

## References

[1] Rosenow F., Lüders H., Presurgical evaluation of epilepsy. Brain 124, 1683–1700 (2001). 11522572 10.1093/brain/124.9.1683

[2] Kramer M. A., Coalescence and fragmentation of cortical networks during focal seizures. J. Neurosci. 30, 10076–10085 (2010). 20668192 10.1523/JNEUROSCI.6309-09.2010 PMC2927849

[3] Schindler K., Forbidden ordinal patterns of periictal intracranial EEG indicate deterministic dynamics in human epileptic seizures. Epilepsia 52, 1771–1780 (2011). 21838792 10.1111/j.1528-1167.2011.03202.x

[4] Truccolo W., Single-neuron dynamics in human focal epilepsy. Nat. Neurosci. 14, 635–641 (2011). 21441925 10.1038/nn.2782 PMC3134302

[5] Schevon C. A., Evidence of an inhibitory restraint of seizure activity in humans. Nat. Commun. 3, 1060 (2012). 22968706 10.1038/ncomms2056 PMC3658011

[6] Wagner F. B., Microscale spatiotemporal dynamics during neocortical propagation of human focal seizures. Neuroimage 122, 114–130 (2015). 26279211 10.1016/j.neuroimage.2015.08.019 PMC4618174

[7] Burns S. P., Network dynamics of the brain and influence of the epileptic seizure onset zone. Proc. Natl. Acad. Sci. U.S.A. 111, E5321–E5330 (2014). 25404339 10.1073/pnas.1401752111 PMC4267355

[8] Karoly P. J., Seizure pathways: A model-based investigation. PLoS Comput. Biol. 14, e1006403 (2018). 30307937 10.1371/journal.pcbi.1006403 PMC6199000

[9] Spencer S. S., Spencer D. D., Williamson P. D., Mattson R. H., Ictal effects of anticonvulsant medication withdrawal in epileptic patients. Epilepsia 22, 297–307 (1981). 7238434 10.1111/j.1528-1157.1981.tb04113.x

[10] Freestone D. R., Karoly P. J., Cook M. J., A forward-looking review of seizure prediction. Curr. Opin. Neurol. 30, 167–173 (2017). 28118302 10.1097/WCO.0000000000000429

[11] King-Stephens D., Lateralization of mesial temporal lobe epilepsy with chronic ambulatory electrocorticography. Epilepsia 56, 959–967 (2015). 25988840 10.1111/epi.13010 PMC4676303

[12] Cook M. J., Human focal seizures are characterized by populations of fixed duration and interval. Epilepsia 57, 359–368 (2016). 26717880 10.1111/epi.13291

[13] Alarcon G., Binnie C. D., Elwes R. D. C., Polkey C. E., Power spectrum and intracranial EEG patterns at seizure onset in partial epilepsy. Electroencephalogr. Clin. Neurophysiol. 94, 326–337 (1995). 7774519 10.1016/0013-4694(94)00286-t

[14] Jiménez-Jiménez D., Prognostic value of intracranial seizure onset patterns for surgical outcome of the treatment of epilepsy. Clin. Neurophysiol. 126, 257–267 (2015). 25065302 10.1016/j.clinph.2014.06.005

[15] Karthick P. A., Tanaka H., Khoo H. M., Gotman J., Prediction of secondary generalization from a focal onset seizure in intracerebral EEG. Clin. Neurophysiol. 129, 1030–1040 (2018). 29571121 10.1016/j.clinph.2018.02.122

[16] Marciani M. G., Gotman J., Effects of drug withdrawal on location of seizure onset. Epilepsia 27, 423–431 (1986). 3720701 10.1111/j.1528-1157.1986.tb03562.x

[17] Naftulin J. S., Ictal and preictal power changes outside of the seizure focus correlate with seizure generalization. Epilepsia 59, 1398–1409 (2018). 29897628 10.1111/epi.14449 PMC6031475

[18] Martinet L. E., Ahmed O. J., Lepage K. Q., Cash S. S., Kramer M. A., Slow spatial recruitment of neocortex during secondarily generalized seizures and its relation to surgical outcome. J. Neurosci. 35, 9477–9490 (2015). 26109670 10.1523/JNEUROSCI.0049-15.2015 PMC4478258

[19] Ewell L. A., Brain state is a major factor in preseizure hippocampal network activity and influences success of seizure intervention. J. Neurosci. 35, 15635–15648 (2015). 26609157 10.1523/JNEUROSCI.5112-14.2015 PMC4659826

[20] Badawy R., Macdonell R., Jackson G., Berkovic S., The peri-ictal state: Cortical excitability changes within 24 h of a seizure. Brain 132, 1013–1021 (2009). 19251759 10.1093/brain/awp017

[21] Gliske S. V., Variability in the location of high frequency oscillations during prolonged intracranial EEG recordings. Nat. Commun. 9, 2155 (2018). 29858570 10.1038/s41467-018-04549-2 PMC5984620

[22] Bazil C. W., Walczak T. S., Effects of sleep and sleep stage on epileptic and nonepileptic seizures. Epilepsia 38, 56–62 (1997). 9024184 10.1111/j.1528-1157.1997.tb01077.x

[23] Khambhati A. N., Davis K. A., Lucas T. H., Litt B., Bassett D. S., Virtual cortical resection reveals push-pull network control preceding seizure evolution. Neuron 91, 1170–1182 (2016). 27568515 10.1016/j.neuron.2016.07.039 PMC5017915

[24] Karoly P. J., The circadian profile of epilepsy improves seizure forecasting. Brain 140, 2169–2182 (2017). 28899023 10.1093/brain/awx173

[25] Takahashi H., Takahashi S., Kanzaki R., Kawai K., State-dependent precursors of seizures in correlation-based functional networks of electrocorticograms of patients with temporal lobe epilepsy. Neurol. Sci. 33, 1355–1364 (2012). 22271261 10.1007/s10072-012-0949-5

[26] Louis Door V., Caparos M., Wendling F., Vignal J.-P., Wolf D., Extraction of reproducible seizure patterns based on EEG scalp correlations. Biomed. Signal Process. Control 2, 154–162 (2007).

[27] Wendling F., Bellanger J.-J., Badier J.-M., Coatrieux J.-L., Extraction of spatio-temporal signatures from depth EEG seizure signals based on objective matching in warped vectorial observations. IEEE Trans. Biomed. Eng. 43, 990–1000 (1996). 9214816 10.1109/10.536900

[28] Wu L., Gotman J., Segmentation and classification of EEG during epileptic seizures. Electroencephalogr. Clin. Neurophysiol. 106, 344–356 (1998). 9741763 10.1016/s0013-4694(97)00156-9

[29] Le Bouquin-Jeannès R., Wendling F., Faucon G., Bartolomei F., Mise en correspondance de relations inter-structures lors de crises d’épilepsie. ITBM-RBM 23, 4–13 (2002).

[30] Wendling F., Shamsollahi M. B., Badier J. M., Bellanger J. J., Time-frequency matching of warped depth-EEG seizure observations. IEEE Trans. Biomed. Eng. 46, 601–605 (1999). 10230138 10.1109/10.759060

[31] Wendling F., Badier J. M., Chauvel P., Coatrieux J. L., A method to quantify invariant information in depth-recorded epileptic seizures. Electroencephalogr. Clin. Neurophysiol. 102, 472–485 (1997). 9216480 10.1016/s0013-4694(96)96633-3

[32] Rummel C., A systems-level approach to human epileptic seizures. Neuroinformatics 11, 159–173 (2013). 22961601 10.1007/s12021-012-9161-2

[33] Schindler K., Leung H., Elger C. E., Lehnertz K., Assessing seizure dynamics by analysing the correlation structure of multichannel intracranial EEG. Brain 130, 65–77 (2007). 17082199 10.1093/brain/awl304

[34] Wendling F., Bartolomei F., Bellanger J. J., Bourien J., Chauvel P., Epileptic fast intracerebral EEG activity: Evidence for spatial decorrelation at seizure onset. Brain 126, 1449–1459 (2003). 12764064 10.1093/brain/awg144 PMC2040489

[35] Schindler K. A., Bialonski S., Horstmann M.-T., Elger C. E., Lehnertz K., Evolving functional network properties and synchronizability during human epileptic seizures. Chaos 18, 033119 (2008). 19045457 10.1063/1.2966112

[36] Schindler K., Elger C. E., Lehnertz K., Increasing synchronization may promote seizure termination: Evidence from status epilepticus. Clin. Neurophysiol. 118, 1955–1968 (2007). 17644031 10.1016/j.clinph.2007.06.006

[37] Kramer M. A., Cash S. S., Epilepsy as a disorder of cortical network organization. Neuroscientist 18, 360–372 (2012). 22235060 10.1177/1073858411422754 PMC3736575

[38] Kramer M. A., Kolaczyk E. D., Kirsch H. E., Emergent network topology at seizure onset in humans. Epilepsy Res. 79, 173–186 (2008). 18359200 10.1016/j.eplepsyres.2008.02.002

[39] Guye M., The role of corticothalamic coupling in human temporal lobe epilepsy. Brain 129, 1917–1928 (2006). 16760199 10.1093/brain/awl151

[40] Bartolomei F., Pre-ictal synchronicity in limbic networks of mesial temporal lobe epilepsy. Epilepsy Res. 61, 89–104 (2004). 15451011 10.1016/j.eplepsyres.2004.06.006

[41] Spencer S. S., Neural networks in human epilepsy: Evidence of and implications for treatment. Epilepsia 43, 219–227 (2002). 11906505 10.1046/j.1528-1157.2002.26901.x

[42] Khambhati A. N., Dynamic network drivers of seizure generation, propagation and termination in human neocortical epilepsy. PLoS Comput. Biol. 11, e1004608 (2015). 26680762 10.1371/journal.pcbi.1004608 PMC4682976

[43] Khambhati A. N., Recurring functional interactions predict network architecture of interictal and ictal states in neocortical epilepsy. eNeuro, 4, ENEURO.0091-16.2017 (2017). 10.1523/ENEURO.0091-16.2017 PMC5343278 28303256

[44] Bettus G., Enhanced EEG functional connectivity in mesial temporal lobe epilepsy. Epilepsy Res. 81, 58–68 (2008). 18547787 10.1016/j.eplepsyres.2008.04.020

[45] Sakoe H., Seibi C., Dynamic programming algorithm optimization for spoken word recognition. IEEE Trans. Acoustics Speech. Signal Process. 26, 43–49 (1978).

[46] Schroeder G. M., Supplementary code and visualisations for "Seizure pathways change on circadian and slower timescales in individual patients with focal epilepsy." Zenodo. 10.5281/zenodo.3692923. Deposited 1 March 2020. PMC7245106 32366665

[47] Sinha S., Brady M., Scott C. A., Walker M. C., Do seizures in patients with refractory epilepsy vary between wakefulness and sleep? J. Neurol. Neurosurg. Psychiatry 77, 1076–1078 (2006). 16914756 10.1136/jnnp.2006.088385 PMC2077754

[48] Fisher R. S., Operational classification of seizure types by the International League Against Epilepsy: Position paper of the ILAE Commission for Classification and Terminology. Epilepsia 58, 522–530 (2017). 28276060 10.1111/epi.13670

[49] Bazil C. W., Seizure modulation by sleep and sleep state. Brain Res. 1703, 13–17 (2019). 29782849 10.1016/j.brainres.2018.05.003

[50] Baud M. O., Multi-day rhythms modulate seizure risk in epilepsy. Nat. Commun. 9, 88 (2018). 29311566 10.1038/s41467-017-02577-y PMC5758806

[51] Harden C. L., Pennell P. B., Neuroendocrine considerations in the treatment of men and women with epilepsy. Lancet Neurol. 12, 72–83 (2013). 23237902 10.1016/S1474-4422(12)70239-9 PMC4928713

[52] Reddy D. S., Rogawski M. A., “Neurosteroids—Endogenous regulators of seizure susceptibility and role in the treatment of epilepsy” in Jasper’s Basic Mechanisms of the Epilepsies, Noebels J. L., Avoli M., Rogawski M. A., Olsen R. W., Delgado-Escueta A. V., Eds. (National Center for Biotechnology Information, Bethesda, MD, ed. 4, 2013), pp. 984–1002.

[53] Taubøll E., Sveberg L., Svalheim S., Interactions between hormones and epilepsy. Seizure 28, 3–11 (2015). 25765693 10.1016/j.seizure.2015.02.012

[54] den Heijer J. M., The relation between cortisol and functional connectivity in people with and without stress-sensitive epilepsy. Epilepsia 59, 179–189 (2018). 29124726 10.1111/epi.13947

[55] Meisel C., Intrinsic excitability measures track antiepileptic drug action and uncover increasing/decreasing excitability over the wake/sleep cycle. Proc. Natl. Acad. Sci. U.S.A. 112, 14694–14699 (2015). 26554021 10.1073/pnas.1513716112 PMC4664307

[56] Engel J. Jr, Crandall P. H., Falsely localizing ictal onsets with depth EEG telemetry during anticonvulsant withdrawal. Epilepsia 24, 344–355 (1983). 6851966 10.1111/j.1528-1157.1983.tb04898.x

[57] Napolitano C. E., Orriols M. A., Changing patterns of propagation in a super-refractory status of the temporal lobe. Over 900 seizures recorded over nearly one year. Epilepsy Behav. Case Rep. 1, 126–131 (2013). 25667845 10.1016/j.ebcr.2013.07.001 PMC4150637

[58] Nevado-Holgado A. J., Marten F., Richardson M. P., Terry J. R., Characterising the dynamics of EEG waveforms as the path through parameter space of a neural mass model: Application to epilepsy seizure evolution. Neuroimage 59, 2374–2392 (2012). 21945471 10.1016/j.neuroimage.2011.08.111

[59] Freestone D. R., Estimation of effective connectivity via data-driven neural modeling. Front. Neurosci. 8, 383 (2014). 25506315 10.3389/fnins.2014.00383 PMC4246673

[60] Jirsa V. K., Stacey W. C., Quilichini P. P., Ivanov A. I., Bernard C., On the nature of seizure dynamics. Brain 137, 2210–2230 (2014). 24919973 10.1093/brain/awu133 PMC4107736

[61] Wenzel M., Hamm J. P., Peterka D. S., Yuste R., Reliable and elastic propagation of cortical seizures in vivo. Cell Rep. 19, 2681–2693 (2017). 28658617 10.1016/j.celrep.2017.05.090 PMC5551439

[62] Wang Y., Mechanisms underlying different onset patterns of focal seizures. PLoS Comput. Biol. 13, e1005475 (2017). 28472032 10.1371/journal.pcbi.1005475 PMC5417416

[63] Proix T., Jirsa V. K., Bartolomei F., Guye M., Truccolo W., Predicting the spatiotemporal diversity of seizure propagation and termination in human focal epilepsy. Nat. Commun. 9, 1088 (2018). 29540685 10.1038/s41467-018-02973-y PMC5852068

[64] Shah P., Characterizing the role of the structural connectome in seizure dynamics. Brain 142, 1955–1972 (2019). 31099821 10.1093/brain/awz125 PMC6598625

[65] Kopell N., Ermentrout G. B., Whittington M. A., Traub R. D., Gamma rhythms and beta rhythms have different synchronization properties. Proc. Natl. Acad. Sci. U.S.A. 97, 1867–1872 (2000). 10677548 10.1073/pnas.97.4.1867 PMC26528

[66] Chapeton J. I., Haque R., Wittig J. H. Jr, Inati S. K., Zaghloul K. A., Large-Scale communication in the human brain Is rhythmically modulated through alpha coherence. Curr. Biol. 29, 2801–2811.e5 (2019). 31422882 10.1016/j.cub.2019.07.014 PMC6736747

[67] Karoly P. J., Circadian and circaseptan rhythms in human epilepsy: A retrospective cohort study. Lancet Neurol. 17, 977–985 (2018). 30219655 10.1016/S1474-4422(18)30274-6

[68] Meisel C., Plenz D., Schulze-Bonhage A., Reichmann H., Quantifying antiepileptic drug effects using intrinsic excitability measures. Epilepsia 57, e210–e215 (2016). 27562603 10.1111/epi.13517

[69] Badawy R. A. B., Macdonell R. A. L., Berkovic S. F., Newton M. R., Jackson G. D., Predicting seizure control: Cortical excitability and antiepileptic medication. Ann. Neurol. 67, 64–73 (2010). 20186859 10.1002/ana.21806

[70] Badawy R. A. B., Jackson G. D., Berkovic S. F., Macdonell R. A. L., Cortical excitability and refractory epilepsy: A three-year longitudinal transcranial magnetic stimulation study. Int. J. Neural Syst. 23, 1250030 (2013). 23273126 10.1142/S012906571250030X

[71] Bardy A. H., Reduction of antiepileptic drug dosage for monitoring epileptic seizures. Acta Neurol. Scand. 86, 466–469 (1992). 1481628 10.1111/j.1600-0404.1992.tb05125.x

[72] Ung H., Temporal behavior of seizures and interictal bursts in prolonged intracranial recordings from epileptic canines. Epilepsia 57, 1949–1957 (2016). 27807850 10.1111/epi.13591 PMC5241889

[73] Baud M. O., Vulliemoz S., Seeck M., Recurrent secondary generalization in frontal lobe epilepsy: Predictors and a potential link to surgical outcome? Epilepsia 56, 1454–1462 (2015). 26212707 10.1111/epi.13086

[74] Goodfellow M., Estimation of brain network ictogenicity predicts outcome from epilepsy surgery. Sci. Rep. 6, 29215 (2016). 27384316 10.1038/srep29215 PMC4935897

[75] Kini L. G., Virtual resection predicts surgical outcome for drug-resistant epilepsy. Brain 142, 3892–3905 (2019). 31599323 10.1093/brain/awz303 PMC6885672

[76] Poldrack R. A., Long-term neural and physiological phenotyping of a single human. Nat. Commun. 6, 8885 (2015). 26648521 10.1038/ncomms9885 PMC4682164

[77] Navis A., Harden C., A treatment approach to catamenial epilepsy. Curr. Treat. Options Neurol. 18, 30 (2016). 27188699 10.1007/s11940-016-0413-6

[78] Scheiermann C., Gibbs J., Ince L., Loudon A., Clocking in to immunity. Nat. Rev. Immunol. 18, 423–437 (2018). 29662121 10.1038/s41577-018-0008-4

[79] Ramgopal S., Thome-Souza S., Loddenkemper T., Chronopharmacology of anti-convulsive therapy. Curr. Neurol. Neurosci. Rep. 13, 339 (2013). 23456771 10.1007/s11910-013-0339-2 PMC3607723

[80] Sanchez-Morillo D., Olaby O., Fernandez-Granero M. A., Leon-Jimenez A., Physiological closed-loop control in intelligent oxygen therapy: A review. Comput. Methods Programs Biomed. 146, 101–108 (2017). 28688479 10.1016/j.cmpb.2017.05.013

[81] Vettoretti M., Facchinetti A., Combining continuous glucose monitoring and insulin pumps to automatically tune the basal insulin infusion in diabetes therapy: A review. Biomed. Eng. Online 18, 37 (2019). 30922295 10.1186/s12938-019-0658-x PMC6440103

[82] Fava G. A., Ruini C., Sonino N., Treatment of recurrent depression: A sequential psychotherapeutic and psychopharmacological approach. CNS Drugs 17, 1109–1117 (2003). 14661988 10.2165/00023210-200317150-00005

[83] Legendre P., Gauthier O., Statistical methods for temporal and space-time analysis of community composition data. Proc. R Soc. B Biol. Sci. 281, 20132728 (2014). 10.1098/rspb.2013.2728 PMC3906937 24430848

[84] Wu S., Stability-driven nonnegative matrix factorization to interpret spatial gene expression and build local gene networks. Proc. Natl. Acad. Sci. U.S.A. 113, 4290–4295 (2016). 27071099 10.1073/pnas.1521171113 PMC4843452

[85] Thomas P. J., Control theory in biology and medicine: Introduction to the special issue. Biol. Cybern. 113, 1–6 (2019). 30701314 10.1007/s00422-018-00791-5

[86] Wang Y., Hutchings F., Kaiser M., Computational Modeling of Neurostimulation in Brain Diseases (Progress in Brain Research, Elsevier B.V., ed. 1, 2015), pp. 191–228. 10.1016/bs.pbr.2015.06.012 26541382

[87] Wagenaar J. B., Brinkmann B. H., Ives Z., Worrell G. A., Litt B., A multimodal platform for cloud-based collaborative research. Int. IEEE EMBS Conf. Neural Eng., 1386–1389 (2013).

[88] Kini L. G., Davis K. A., Wagenaar J. B., Data integration: Combined imaging and electrophysiology data in the cloud. Neuroimage 124, 1175–1181 (2016). 26044858 10.1016/j.neuroimage.2015.05.075 PMC4651831

[89] Farooque P., Duckrow R., Subclinical seizures during intracranial EEG recording: Are they clinically significant? Epilepsy Res. 108, 1790–1796 (2014). 25306063 10.1016/j.eplepsyres.2014.09.020

[90] Martinet L.-E., Human seizures couple across spatial scales through travelling wave dynamics. Nat. Commun. 8, 14896 (2017). 28374740 10.1038/ncomms14896 PMC5382286

[91] Lee D. D., Seung H. S., Learning the parts of objects by non-negative matrix factorization. Nature 401, 788–791 (1999). 10548103 10.1038/44565

[92] Aggarwal C. C., Hinneburg A., Keim D. A., “On the surprising behavior of distance metrics in high dimensional space” in Database Theory — ICDT 2001, Van den Bussche J., Vianu V., Eds. (Lecture Notes in Computer Science, Springer, Berlin, 2001), Vol. 1973, pp. 420–434.

[93] Mantel N., The detection of disease clustering and a generalized regression approach. Cancer Res. 27, 209–220 (1967). 6018555

[94] Benjamini Y., Hochberg Y., Controlling the false discovery rate: A practical and powerful approach to multiple testing. J. R. Stat. Soc. B 57, 289–300 (1995).

[95] Kim J., He Y., Park H., Algorithms for nonnegative matrix and tensor factorizations: A unified view based on block coordinate descent framework. J. Glob. Optim. 58, 285–319 (2014).

[96] Kim J., Park H., Fast nonnegative matrix factorization: An active-set-like method and comparisons. SIAM J. Sci. Comput. 33, 3261–3281 (2011).

