# Supplementary material for: Seizure pathways change on circadian and slower timescales in individual patients with focal epilepsy
Source: Proc Natl Acad Sci U S A. 2020 May 4;117(20):11048–58. doi: 10.1073/pnas.1922084117 (PMC7245106; doi:10.1073/pnas.1922084117)
Supplement: Supplementary File [file pnas.1922084117.sapp.pdf]

PNAS
www.pnas.org

Supplementary Information for
Seizure pathways change on circadian and slower timescales in individual patients with focal epilepsy

Gabrielle M. Schroeder[a], Beate Diehl[b], Fahmida A. Chowdhury[b], John S. Duncan[b], Jane de Tisi[b], Andrew J. Trevelyan[c], Rob Forsyth[c], Andrew Jackson[c], Peter N. Taylor[a,b,c], Yujiang Wang*[a,b,c]

a. Interdisciplinary Computing and Complex BioSystems Group, School of Computing Science, Newcastle University, Newcastle upon Tyne, NE4 5TG, United Kingdom
b. UCL Queen Square Institute of Neurology, University College London, London, WC1N 3BG, United Kingdom
c. Faculty of Medical Sciences, Newcastle University, Newcastle upon Tyne, NE2 4HH, United Kingdom

***Corresponding author**
Yujiang Wang
School of Computing, Urban Sciences Building, Newcastle University, 1 Science Square, Newcastle Helix, Newcastle upon Tyne, NE4 5TG, United Kingdom
yujiang.wang@ncl.ac.uk

This PDF file includes

- Text S1: Patient metadata
- Text S2: Overview of main analyses
- Text S3: Dynamic time warping of example seizures
- Text S4: Summary of main analysis results of all patients
- Text S5: Analysis of seizure dissimilarities within frequency bands
- Text S6: Clustering seizures based on seizure dissimilarities
- Text S7: Seizure variability is not driven by differences in seizure clinical type
- Text S8: No relationship between features of seizure variability and clinical measures
- Text S9: No relationship between patterns of seizure dissimilarities and AED reduction
- Text S10: Supplementary modelling details and results
- Text S11: Dimensionality reduction using non-negative matrix factorization
- Text S12: Comparison of seizure dissimilarity to metric distances

www.pnas.org/cgi/doi/10.1073/pnas.1922084117

Text S1: Patient metadata

Table S1: Patient metadata

patient	hospital	age (yrs)	sex	hemisphere	lobe	pathology	ILAE surgical outcome	total recording time	# seizures analysed	# electrodes analysed	sampling frequencies	AED reduction performed?
**Study 012-2**	MC	37	M	B	T	Other	-	13d 16h	28	81	499.907 Hz	-
**Study 017**	MC	39	M	R	FT	Other	4	7d 17h	9	16	499.907 Hz	-
**Study 019**	MC	33	M	L	T	-	5	5d 16h	33	96	499.907 Hz	-
**Study 020**	MC	10	M	R	F	-	4	5d	8	55	499.907 Hz	-
**Study 021**	MC	16	M	R	FT	Other	1	6d 11h	13	108	500 Hz	-
**Study 024**	MC	23	F	B	TP, IH	-	-	8d 10h	12	83	500 Hz	-
**Study 026**	MC	9	M	L	F	FCD	1	3d 3h	21	81	499.907 Hz	-
**Study 027**	MC	34	F	L	T	HS	-	3d 21h	6	47	500 Hz	-
**Study 030**	MC	18	F	L	FP	FCD	3	5d 23h	8	63	500 Hz	-
**Study 033**	MC	3	M	L	F	TS	5	6d 17h	17	127	500 Hz	-
**Study 037**	MC	62	F	R	F	-	-	8d 23h	8	78	499.907 Hz	-
**Study 038**	MC	58	M	L	FT	-	1	3d	10	86	500 Hz	-
**I002_P006_D01**	HUP	26	F	R	T	-	-	12d 22h	7	83	512 Hz	yes
**95**	UCLH	35	M	L	OP	Other	4	7d 1h	13	56	512 Hz, 1024 Hz	no
**756**	UCLH	38	F	B	T	Other	3	6d 19h	6	20	1024 Hz	yes
**770**	UCLH	25	F	L	P	FCD	3	4d 3.6h	8	71	512 Hz	no
**821**	UCLH	25	F	L	T	HS, BDI	1	6d 4h	9	46	512 Hz	yes
**931**	UCLH	28	M	L	T	HS	4	7d	11	58	512 Hz	yes
**934**	UCLH	28	F	R	OP	TS	1	1d 19h	40	76	512 Hz	no
**999**	UCLH	28	M	L	F	FCD	1	12d 5h	26	73	512 Hz	yes
**1005**	UCLH	21	F	R	T	HS	2	8d 21h	15	85	512 Hz	yes
**1097**	UCLH	28	M	L	F	GL	1	1d 20h	8	84	512 Hz	no
**1109**	UCLH	31	F	R	T	CAV	1	6d 1h	13	53	1024 Hz	yes
**1149**	UCLH	43	F	R	TOP	DNT	1	7d 22h	24	62	512 Hz, 1024 Hz	no
**1163**	UCLH	27	F	L	F	FCD	1	8d	8	111	512 Hz	yes
**1167**	UCLH	39	M	L	P	CAV	4	5d 22h	43	51	1024 Hz	no
**1168**	UCLH	60	F	L	F	FCD	2	2d	10	94	512 Hz	no
**1182**	UCLH	28	M	R	P	FCD	3	5d 5h	52	75	512 Hz	no
**1196**	UCLH	41	M	R	T	HS	3	15d 22h	11	34	1024 Hz	yes
**1200**	UCLH	24	F	R	T	HS	1	2d 19h	14	71	512 Hz	yes
**1211**	UCLH	26	M	R	T	Other	3	5d 7h	20	77	512 Hz	yes

Table S1 lists the metadata for the patients whose seizures were analysed in this study. Patient identifiers are listed under "patient." For IEEG Portal patients (MC and HUP hospitals), their identifier is the same as the one used by the database. Metadata was extracted from the reports provided on the IEEG Portal (MC and HUP patients) or the patient clinical reports (UCLH patients). For each patient, the following information is provided:

- hospital: hospital at which the patient underwent presurgical monitoring (MC = Mayo Clinic, HUP = Hospital of the University of Pennsylvania, UCLH = University College London Hospital).
- age: age, in years, at the time of the presurgical monitoring.
- sex: patient sex (M = male, F = female).
- hemisphere: purported hemisphere of onset of the patient's seizures (L = left, R = right, B = bilateral), based on clinical findings.
- lobe: purported lobe of onset of the patient's seizures (T = temporal, F = frontal, P = parietal, O = occipital, IH = interhemispheric), based on clinical findings. Note that some patients had seizures arising from multiple lobes/at the boundary of two lobes (e.g., OP = occipital/parietal onset).
- pathology: postoperative tissue pathology findings (FCD = Focal cortical dysplasia, BDI = Brain damage - inflammatory, HS = Hippocampal sclerosis, TS= Tuberous sclerosis, GL = Glioma, CAV = Cavernoma, DNT = Dysembryoplastic neuroepithelial tumour, Other = other type of pathology that is not one of the other categories). A dash indicates that this information is unavailable.
- ILAE surgical outcome: patient surgical outcome according to the International League Against Epilepsy classification (1 = seizure free, 2 = only auras, 3+ = not seizure free). A dash indicates that the patient did not undergo surgery or their surgical outcome is unavailable. For IEEG Portal patients (MC and HUP hospitals), the surgical outcome provided by the database is given. For UCLH patients, the 12 months post-surgical outcome is provided.

- total recording time: total duration of the presurgical intracranial recording time.
- # seizures analysed: number of the patient's seizures analysed in this work.
- # electrodes analysed: number of recording electrodes included in the analysis, after removing noisy electrodes.
- sampling frequencies: sampling frequencies at which intracranial data was acquired and stored.
- AED reduction performed: whether patient antiepileptic drugs (AEDs) were systematically reduced during the presurgical recording. A dash indicates that this information is unavailable.

Text S2: Overview of main analyses

The main analyses in the paper, along with their purposes and the approaches used, are included in Fig. S2. The figure references indicate which figures in the main text provide an overview of the method and/or results. Arrows indicate where output from one analysis is used as input for another analysis. See main text Methods and Results for detailed descriptions of the analyses, outputs, and visualisations.

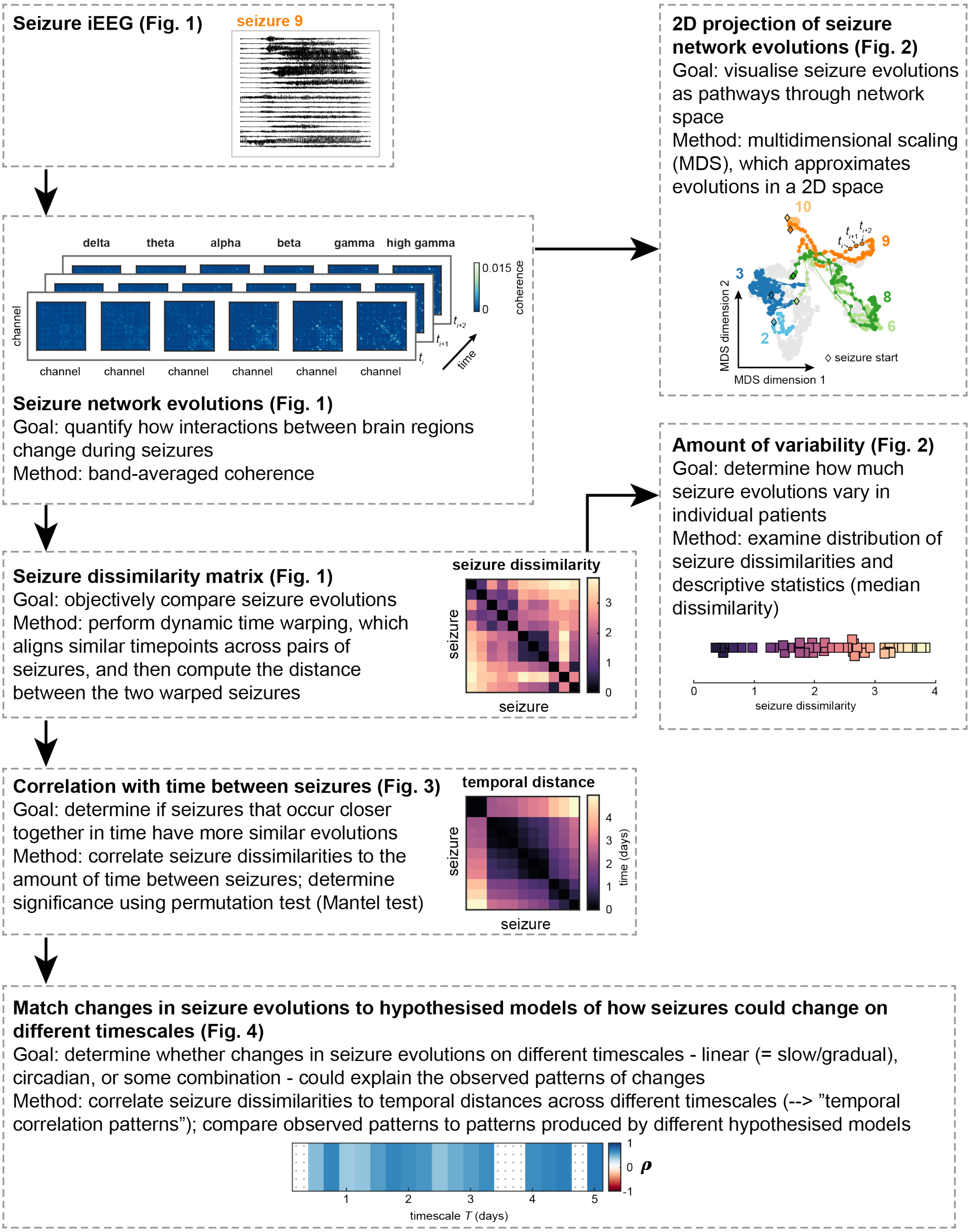

Fig. S2: Overview of the main analyses in "Seizure pathways change on circadian and slower timescales in individual patients with focal epilepsy."

Text S3: Dynamic time warping of example seizures

In this section, we demonstrate how dynamic time warping aligns similar dynamics across seizures. In our application, this warping allows us to identify seizures with similar network evolutions, even if the rates of the evolutions differ.

Dynamic time warping selectively "stretches" two time series in order to minimise the total distance between them. In our application, the distance between two time series reflects the difference between the network evolutions of two seizures. Importantly, dynamic time warping can *only* stretch each time series in order to align similar dynamics, which means that the algorithm cannot

1) skip time points; instead, all time points of both time series, including beginning points, must be included in the warp path.
2) repeat earlier time points; i.e., the warp path cannot double back on itself in order to repeatedly include a section of one of the time series.

The warping process is also repeated individually for each pair of seizures, and the warp length and path will therefore differ between different pairs of seizures. In order words, this algorithm provides a *pairwise* alignment of time series, rather than a multiple time series alignment. We focus on pairwise alignments because a global warping solution would likely sacrifice the optimal alignment of some pairs of time series.

To understand how dynamic time warping aligns pairs of time series, consider two seizures, seizure A with M windows, and seizure B with N windows. From these two seizures, we compute the time-time distance matrix D (Fig. S3a). This MxN matrix contains the pairwise distance between the functional connectivity of each pair of time windows across the two seizures: D(m,n) is the distance between the functional connectivity of the m-th time window of seizure A and the n-th time window of seizure B. Thus, the row of D corresponds to the time window index of seizure A, while the column of D corresponds to the time window index of seizure B.

Dynamic time warping finds a path through this distance matrix that minimises the total distance (here, the L1 norm distance) between the two seizures. The algorithm only allows three categories of moves through the matrix (Fig. S3a):

1) Horizontal moves, D(m,n) → D(m,n+1), stretch seizure A (blue arrow)
2) Vertical moves, D(m,n) → D(m+1,n), stretch seizure B (red arrow)
3) Diagonal moves, D(m,n) → D(m+1,n+1), do not stretch either seizure (purple arrow)

We demonstrate this process by visualising the time-time distance matrices and warp paths of three pairs of patient 1109's seizures (Fig. S3b-d). Each time-time distance matrix is computed by calculating the L1 norm distance between the functional connectivity vectors (reconstructed following NMF – see Supplementary Fig. S11.2) of each pair of time windows in the two seizures. Low distances reveal pairs of time windows with similar functional network dynamics. Points in the warp path (red squares), laid over the time-time distance matrix, indicate which pairs of indices were aligned. Finally, to calculate the seizure dissimilarity between two seizures, we average over the pointwise distances along the warp path. To visually demonstrate how DTW aligns similar time windows, we additionally assigned each seizure time window to a network state (see Supplementary Fig. S11.2); each seizure can therefore be described as a progression of different network states. Time windows with the same state have similar functional network connectivity and should therefore, when possible, be aligned across different seizures.

To provide a simple example of the warping process, we first examine the warp path of seizures 7 and 12, which have a relatively low seizure dissimilarity (0.62) (Fig. S3b). Both seizures begin with network state 3 (orange) and then transition to network state 1 (aqua). While the duration of state 3 is the same in both seizures, state 1 lasts longer in seizure 12 than seizure 7. As such, window 7 of seizure 7 is repeated in order to align the two seizures, as shown by the horizontal line in the warp path.

Fig. S3c shows the warp path of seizures 3 and 4, which have similar state progressions but differ in the rate of their state progressions. However, because dynamic time warping aligns the similar parts of each seizure, there is also a relatively low seizure dissimilarity between these two seizures (0.52). These two seizures exemplify how certain windows are stretched to accommodate longer state durations in the other seizure: horizontal lines in the path correspond to places where seizure 3 is stretched, while vertical lines correspond to time points in seizure 4 that are repeated. For example, in seizure 3, a window of the last state (state 5, green) is stretched so that it matches the dynamics of state 4. Notably, the brief transition of seizure 4 to state 6 (dark orange) cannot be matched by any time point in seizure 3 because the warp path cannot skip this window or align it to earlier windows. In seizure 4, the longest warpings occur during state 3 (orange) and state 2 (wine red), which are both longer in seizure 3. Thus, by warping the seizure time series, our method recognises parts of the seizures that have similar network dynamics, despite differences in the rates of the seizure evolutions.

Finally, Fig. S3d demonstrates how dynamic time warping aligns two seizures, seizures 3 and 6, that have somewhat different state progressions. Compared to seizure 3, seizure 6 spends more time in state 2 (wine red) and never progresses to seizure 3's final state, state 5 (green). As such, in seizure 3, a window of state 2 is stretched. However, because the warp path must include all time points, the final part of seizure 6 must be matched to the final dynamics of seizure 3, even though there are high distances between these time points. This difference between the final seizure dynamics raises the dissimilarity between seizures 3 and 6: it is 1.03, which is almost double the previously examined dissimilarity between seizures 3 and 4.

Note that seizures 3 and 6 provide an example of when seizure pathways only partially match: the dynamics are initially very similar, and seizure 3 diverges by progressing to an additional state. In these cases, dynamic time warping cannot find a close alignment between the entire seizure progressions, and our seizure dissimilarity measure depends on the durational proportion of the mismatch. In other words, had the green state lasted longer in seizure 3, the seizure dissimilarity measure would be higher in this example. In some cases, this dependence on state duration also means that the seizure dissimilarity measure is not a metric distance. In Supplementary S12 we compare seizure dissimilarities, computed using dynamic time warping, to two alternative metric distance measures for comparing seizure pathways.

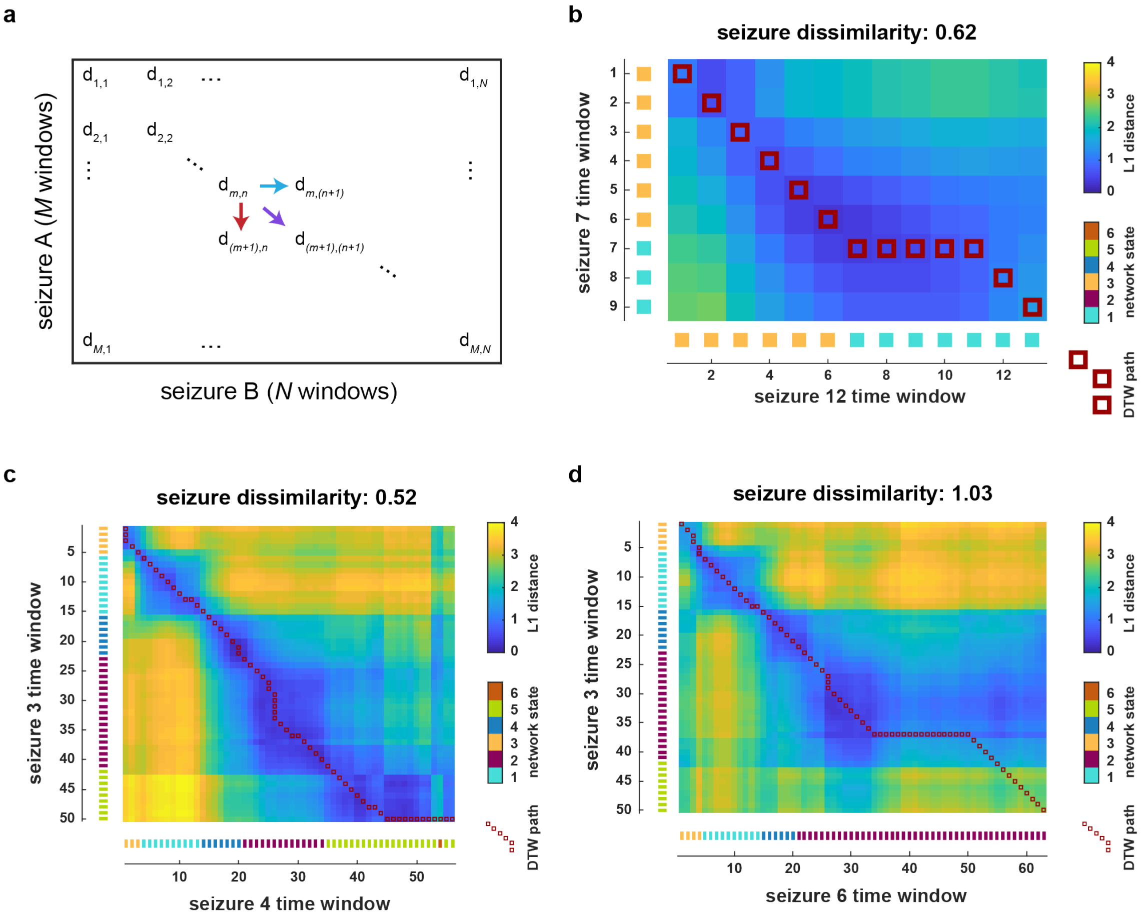

Fig. S3: Visualising the optimal path found by dynamic time warping. (a) The time-time distance matrix, D_{MxN}, contains the distance between each pair of time windows across two seizures. A warp path must start at D(1,1) and move horizontally, vertically, or diagonally until it reaches D(M,N). (b-d) The warp path and time-time distance matrices of patient 1109's (b) seizures 7 and 12, (c) seizures 3 and 4, and (d) seizures 3 and 6. Each heatmap shows the L1 distance between the functional network evolutions of the pair of seizures. Low distances, in blue, indicate time points with similar network dynamics. Red squares indicate the warp path chosen by the dynamic time warping algorithm; if entry (i,j) is part of the warp path, then the corresponding time windows of the two seizures were aligned. For example, in (b), seizure 7's time window 7 is aligned to seizure 12's time windows 7-11 . Along each side of the time-time distance matrix, the network state of each time point is shown. Note that since the time-time distance matrix is calculated from the functional connectivity time courses, and not the simplified state descriptions, there are non-zero distances between points assigned to the same state. However, the network state progressions provide a useful visualisation for how the warp path aligns comparable network states.

Text S4: Summary of main analysis results of all patients

Table S4: Summary of main analysis results of all patients

patient	number of seizures	number of clusters	number of states	correlation with temporal distance			Model matching temporal correlation pattern
				rho	*p-value*	*q-value*	
**Study 012-2**	28	2	8	0.57	<0.0001	<0.0002	Linear
**Study 017**	9	1	4	0.28	0.0990	0.1228	Other/indeterminate
**Study 019**	33	1	9	0.43	<0.0001	<0.0002	Linear
**Study 020**	8	1	7	0.48	0.0118	0.0205	Linear
**Study 021**	13	1	5	0.67	0.0001	0.0002	Linear
**Study 024**	12	2	6	0.67	<0.0001	<0.0002	Linear
**Study 026**	21	1	4	0.53	<0.0001	<0.0002	Linear
**Study 027**	6	1	7	0.56	0.0119	0.0205	Linear + circadian
**Study 030**	8	2	8	0.72	0.0216	0.0319	Linear
**Study 033**	17	1	4	-0.10	0.8567	0.8567	Other/indeterminate
**Study 037**	8	1	10	0.58	0.0161	0.0263	Linear
**Study 038**	10	1	8	0.19	0.1572	0.1805	Other/indeterminate
**I002_P006_D01**	7	1	7	0.83	0.0035	0.0072	Linear + circadian
**95**	13	2	3	0.71	<0.0001	<0.0002	Linear
**756**	6	2	6	0.18	0.1724	0.1909	Linear
**770**	8	1	5	0.09	0.2598	0.2685	Circadian
**821**	9	2	4	0.17	0.1304	0.1555	Linear + circadian
**931**	11	1	5	0.69	0.0001	0.0002	Linear
**934**	40	2	3	0.63	<0.0001	<0.0002	Linear
**999**	26	1	5	0.64	<0.0001	<0.0002	Linear
**1005**	15	1	4	0.69	<0.0001	<0.0002	Linear + circadian
**1097**	8	1	3	0.40	0.0494	0.0666	Linear
**1109**	13	3	6	0.24	0.0527	0.0681	Linear
**1149**	24	1	8	0.42	0.0001	0.0002	Linear
**1163**	8	1	8	0.12	0.2559	0.2685	Other/indeterminate
**1167**	43	2	5	0.22	0.0115	0.0205	Circadian
**1168**	10	2	2	0.31	0.0363	0.0512	Linear + circadian
**1182**	52	2	6	0.65	<0.0001	<0.0002	Linear + circadian
**1196**	11	1	5	0.32	0.0208	0.0319	Linear + circadian
**1200**	14	1	4	0.69	<0.0001	<0.0002	Linear
**1211**	20	1	4	0.38	0.0003	0.0007	Circadian

Table S4 summarises the main analysis results of all patients. See main text Methods for a detailed description of the analysis. Patient identifiers are listed under "patient." The next columns of the table provide the following information for each patient:

- number of seizures: the number of seizures analysed.
- number of clusters: the number of non-hierarchical seizure clusters, based on the seizure dissimilarity matrix.
- number of states: the optimal number of network states (i.e., NMF basis vectors), which was used to reduce noise in the dataset. See Supplementary Fig. S11.1 for details on finding the optimal number of states.

The table then provides the following information about the correlation between seizure dissimilarities and temporal distances (amount of time elapsed between seizure start times) in each patient:

- rho: the Spearman correlation between seizure dissimilarities and temporal distances
- *p*-value: the *p*-value of the correlation, based on permutation tests (10,000 permutations). If a value greater than or equal to the observed correlation was not observed, then the *p*-value is listed as < 0.0001.

- *q*-value: the *q*-value, or adjusted *p*-value, of the correlation after global false discovery rate correction on all *p*-values in the table (31 total statistical tests).

Significant correlations (defined as $q < 0.05$) are indicated with blue text for the correlation and the corresponding *p*- and *q*-values. We note that additional statistical tests were performed in the SI Appendix, and none of these tests yielded significant results. If a global false discovery rate correction is instead performed using the sixteen *p*-values from the supplementary sections (one from the SI Appendix, Text S6, fourteen from the SI Appendix, Text S8, and one from the SI Appendix, Text S9) in addition to the above 31 *p*-values, the same *p*-values remain significant.

The final column in the table ("Model matching temporal correlation pattern") indicates the type of model consistent with each patient's temporal correlation pattern (see "Seizure pathways change on different timescales" in main text). Detailed modelling results are provided in Supplementary S10.

Visualisations of the analysis results of each patient are available on Zenodo at http://dx.doi.org/10.5281/zenodo.3692923.

Text S5: Analysis of seizure dissimilarities within frequency bands

In this section, we determine if variability in seizure network evolutions were driven by network differences in a particular frequency band. In each patient, we computed the contribution of each frequency band's functional connectivity to the observed dissimilarity of each pair of seizures. This analysis yielded a set of six additional matrices (one for each frequency band) that gave the dissimilarity of each pair of seizures within the given frequency band. Note that this analysis is *not* equivalent to recomputing seizure dissimilarities using only the given frequency band, as information across *all* frequency bands was used to determine the optimal warp path between the two seizures. However, this analysis reveals 1) whether network differences in specific frequency bands are consistent with the information found across frequency bands, and 2) the proportion of the observed dissimilarities that are due to differences within each frequency band.

Fig. S5A shows the contribution of each frequency band to the dissimilarity of each pair of seizures in Patient 931. Since these contributions are computed for each pair, they can be visualised as a set of six (one per frequency band) additional seizure by seizure matrices. The magnitude of the values in each band matrix is lower compared to the patient's seizure dissimilarity matrix (far left) because only a proportion of the original features were used to compute the frequency band contributions. However, notably, each frequency band contribution matrix has a similar structure to the patient's seizure dissimilarity matrix. This observation is quantified by the high correlation between each band matrix and the original seizure dissimilarity matrix; seizure pairs are ranked similarly regardless of whether differences in just one frequency band or across all frequency bands are taken into account. Fig. S5B further visualises this relationship using scatter plots of each of the frequency band contributions vs. the total seizure dissimilarities. Across all patients, the correlations between the patient's seizure dissimilarities and the contributions of each frequency band are also high (Fig. S5C). These results indicate that the relative contributions of each frequency band are approximately consistent across seizures.

Fig. S5D and Fig. S5E present the average and percentage contribution, respectively, of each frequency band to the seizure dissimilarities of each patient. Although there is variability between patients in the average overall seizure dissimilarity, each frequency band contributes a similar proportion of the observed dissimilarities across patients.

Although this analysis suggests that similar seizure dissimilarity matrices might be obtained by limiting our analysis to one frequency band or performing the broadband analysis, we computed seizure dissimilarities across frequency bands for a few reasons. First, although on average, each frequency band contributes similarly to the observed dissimilarities, the contribution of each frequency band will vary somewhat from seizure to seizure. This variability is evident in the less-than-one (albeit high) correlations between total seizure dissimilarities and frequency band contributions (Fig. S5C); certain frequency bands are slightly more important for a subset of the seizure comparisons in certain patients. The importance of certain frequency bands may depend on the frequency contribution of seizures; for example, network activity in lower frequency bands may be more important in comparisons with secondarily generalised seizures. Second, although the percentage contribution of each frequency band is similar across patients (Fig. S5E), there are some discrepancies between patients. As such, it is fairer to compare the magnitude of seizure dissimilarities across patients if we incorporate information from all frequency bands. Finally, using network information across all frequency bands allows us to warp pairs of seizures and align their dynamics based on changes in any frequency band.

Additionally, we emphasise that these results do *not* mean that the network evolution of a seizure is the same across different frequency bands. Indeed, we observed that seizure network structure

differed across frequency bands in a given time window (see Fig. S11.2 for an example; the provided Zenodo scripts can also generate these visualisations for each patient). Further work is needed to determine 1) how network interactions differ across frequency bands within the same seizure, 2) how changes in one frequency band affect network interactions in another frequency band, and 3) whether seizure network evolutions in a certain frequency band is more closely related to interictal/preictal network activity.

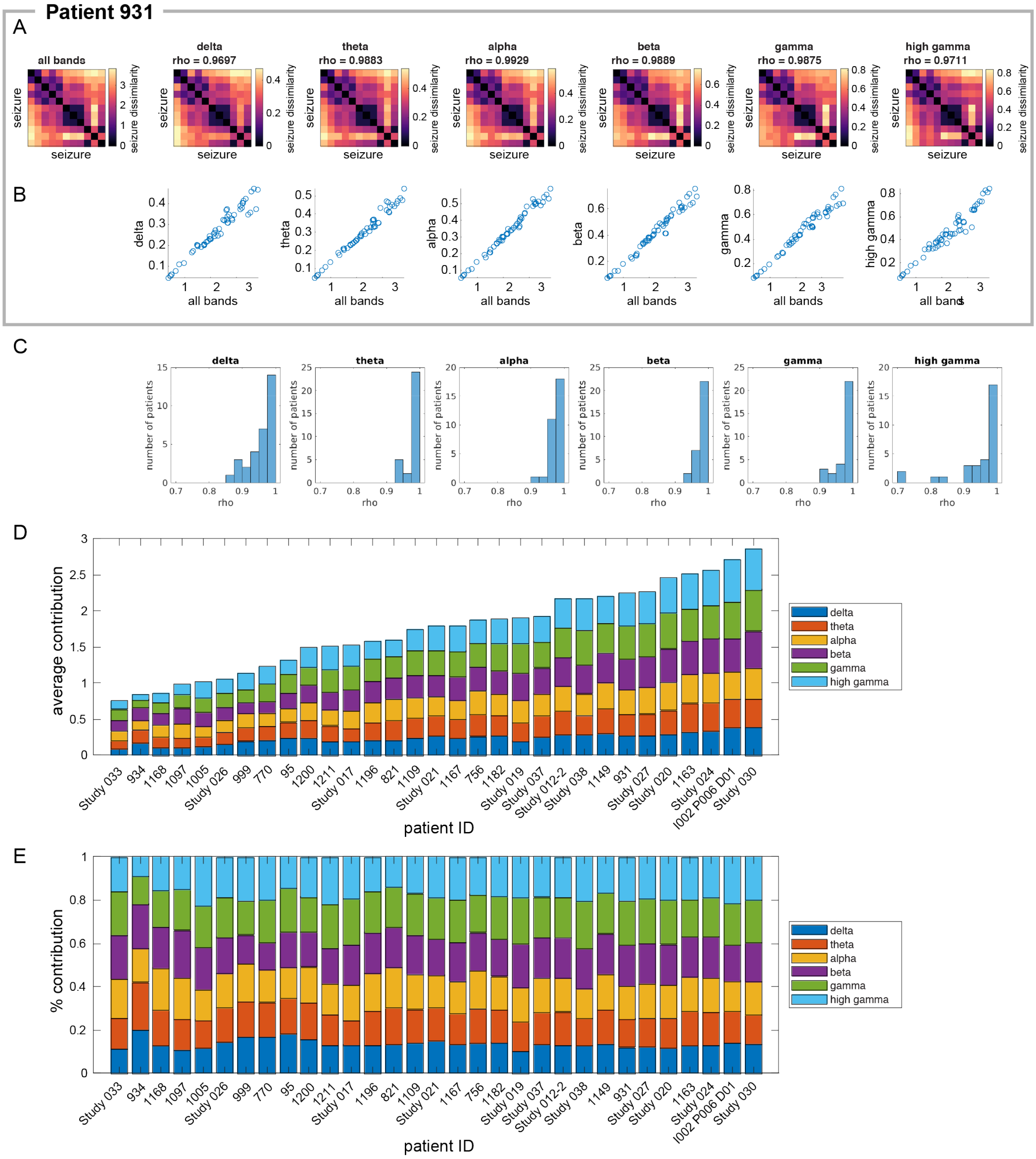

Fig. S5: Contribution of each frequency band to seizure dissimilarities. A) The amount that network differences within each frequency band contribute to the dissimilarity of each pair of seizures in Patient 931. Because these contributions are computed for each pair of seizures, they can be visualised as matrices. For reference, the patient's seizure dissimilarity matrix (computed across all frequency bands) is shown on the left. Spearman's correlation between this matrix and each of the frequency band contribution matrices is provided above each band matrix. B) Scatter plots of the frequency band contributions vs. seizure dissimilarities for Patient 931. Each point corresponds to one seizure pair. C) Histograms of Spearman's correlations between seizure dissimilarities and the frequency band contribution matrices in all patients. D) The amount each frequency band contributes to seizure dissimilarities, on average (i.e., the mean amount across seizure pairs), in each patient. Total bar height is equal to the mean seizure dissimilarity of each patient, and patients are sorted from lowest to highest mean seizure dissimilarity. E) The percentage each frequency band contributes, on average, to the seizure dissimilarity of each seizure pair. Patients are sorted the same as in Fig. S5D.

Text S6: Clustering seizures based on seizure dissimilarities

Seizure clustering and cluster evaluation

Past studies have noted that some patients have multiple populations of seizures with distinct features such as different onset sites (1, 2) or durations (3, 4). As such, we would expect the variability described in these studies to result from different, discrete seizure pathways coexisting in the same patient. We therefore tested if each of the patients in our cohort had separable classes of seizure pathways by clustering their seizures based on seizure dissimilarities.

Specifically, to identify groups of similar seizures in each patient, each patient's seizures were hierarchically clustered by using the seizure dissimilarity matrix as input for an agglomerative hierarchical clustering algorithm, UPGMA (unweighted pair group method with arithmetic mean). The hierarchical clustering resulted in a dendrogram that summarised the similarity between the patient's seizures. Note that the hierarchical clustering representation was an approximation of the seizure dissimilarities that forced all dissimilarities into a metric space.

The gap statistic (5), which compares the within-cluster dispersion of a given clustering relative to a reference (null) distribution, was then used to determine if optimal flat (i.e., non-hierarchical) clusters of seizures existed in each patient. In order to generate reference datasets, the patient's seizures were first projected into Euclidean space using classical (Torgerson's) multidimensional scaling (MDS). Note that this step differs from the earlier visualisation of seizure pathways, which projected seizure time points, rather than seizures themselves. Given the seizure dissimilarity matrix, MDS assigned a coordinate point to each seizure while attempting to preserve the specified dissimilarities between seizures. In order to most closely approximate the dissimilarities matrix, the seizures were projected onto the maximum possible number of dimensions; note, however, that like the hierarchical clustering, MDS also provided a metric approximation of the nonmetric dissimilarities. One thousand reference datasets were then generated by drawing coordinates from a uniform distribution placed over a box aligned with the principal components of the projected seizure data. Each reference dataset was hierarchically clustered by computing the distances between the coordinate points and applying the UPGMA algorithm. To test for flat clusters in the seizure data and reference datasets, the dendrograms were cut at different levels to generate 1, 2, …. s clusters, where s is the number of seizures. At each number of clusters k, the gap statistic $G(k)$ was computed by comparing the within-cluster dispersion of the observed seizures and the reference datasets. The multiple reference datasets also allowed calculation of the standard error of the gap statistic at each k, $SE(k)$. The optimal number of clusters was defined as the smallest number of clusters where $G(k) \geq G(k+1) - SE(k+1)$, which identifies the point at which increasing the number of clusters provides little improvement in the clustering of the data (5).

Note that if the gap statistic identifies a single (i.e., one) cluster in the data, this result does *not* mean that the data is uniform and all belongs to a single, homogenous group. Cluster assignment only indicates whether a subset of the data contains observations that are more similar to themselves than to the other observations; it does not provide any information about whether there is still variability within the cluster. Thus, a finding of one cluster indicates that the data is not separable into different groups, but does not mean that all of the data is uniform. In our case, we found that patients with a single cluster still had variability in seizure pathways, and we therefore refer to seizures in these patients as having a "spectrum" (rather than "one cluster") of seizures to avoid confusion. Likewise, even if multiple clusters are found, there can still be high levels of variability within each group (see next section, "Amount of variability

within and between seizure clusters"); the within-cluster differences must just be less than the between-cluster differences in that particular clustering solution.

Using this approach, we clustered seizures within each patient (Fig. S6.1). Contrary to our expectation, the majority of patients (21 patients), including patient 931, did not have distinct groupings of seizures pathways (Fig. S6.1B). However, there is still variability in seizure pathways in these patients, with the different seizures forming a spectrum of pathways. Without a clear way to split their seizures into different categories, the full diversity of each patient's seizure pathways could not be described by a few example seizures. Ten patients had two or more seizure clusters, although there was still variability in pathways within most clusters (see next section, "Amount of variability within and between seizure clusters"). The average amount of seizure variability was the same in patients with or without multiple seizure clusters (Fig. S6.1C) (two sample t-test, $p = 0.68$). Thus, the presence or absence of different groups of seizure pathways does not indicate the average amount of seizure variability in each patient.

Amount of variability within and between seizure clusters

As described above, each patient either had a spectrum of seizures (one seizure cluster, with variability in seizure pathways within that cluster) or two or more seizure clusters (i.e., their seizure pathways could be grouped into different classes of dynamics, with more similarity within a group than between groups). Fig. S6.2 shows the median level of seizure dissimilarity of seizures within the same cluster and the median level of seizure dissimilarity of seizures from different clusters in each patient. Overall, in patients with multiple seizure clusters (purple histograms), the average seizure dissimilarity *between* clusters is higher than the average seizure dissimilarity *within* clusters, as expected. However, there is overlap in the distributions of within- and between-cluster seizure dissimilarity, demonstrating that seizures in the same cluster can be relatively different, while seizures in different clusters can be relatively similar. In other words, a given cluster can represent *different* pathways that are, despite their diversity, still more similar to each other than to the patient's other seizure pathways. Meanwhile, in some patients, the different groups of seizure pathways, represented by different clusters, are still relatively similar, indicating overall lower variability in seizure evolutions in that patient.

Clustering using alternative algorithm (k-means)

To provide additional confidence in our clustering results, we repeated the seizure clustering using k-means clustering instead of hierarchical, clustering. Because the k-means algorithm requires the observations to be in a metric distance space, we performed this clustering on the MDS embedding of the seizures (see "Seizure clustering and cluster evaluation"), rather than using the seizure dissimilarity matrix. As above, the gap statistic was then used to determine the optimal number of seizure clusters. Of the 31 patients, 28 patients had both the same optimal number of seizure clusters and the same seizure partitioning (i.e., assignment of seizures to clusters) regardless of the clustering algorithm (hierarchical clustering or k-means clustering). Thus, in the majority of patients, we obtained equivalent clustering results using a different clustering algorithm

We next looked at the three patients whose clustering solutions differed. The two patients whose optimal number of clusters changed were patients 1167 and 1182, who had two clusters based on hierarchical clustering, but four and eight clusters, respectively, using k-means clustering. We compared these finer partitions to the hierarchical clustering of each patient by cutting each patient's dendrogram at the level required to make four and eight clusters, respectively. The different clustering solutions can then be compared using the Rand Index, which is the proportion of pairs of elements (here, seizures) that have the same relative

clustering assignment in both solutions; i.e., the two elements are in the same cluster in both solutions or are in different clusters in both solutions. The Rand Index will be one if the two clustering solutions are exactly the same. Although these clustering solutions differed from the k-means clusters, there was a high level of overlap (Rand index of 0.78 for patient 1167 and 0.9 for patient 1182), indicating that both clustering solutions nonetheless capture similar information. Finally, Patient Study 012-2 had two optimal clusters in both cases, but the partitions slightly differed; namely, the cluster assignment of two of the patient's 28 seizures changed, resulting in a Rand index of 0.86.

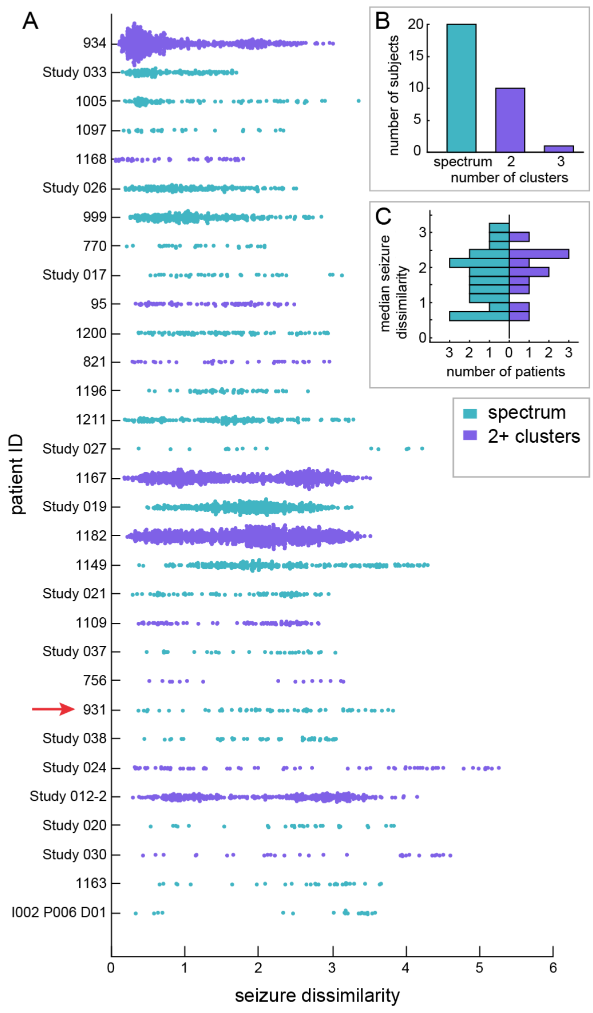

Fig. S6.1: Variability in seizure pathways is common in all patients, but may take the form of either a spectrum or clusters of seizure pathways. A) Distributions of seizure dissimilarities in each patient. This figure is a modified version of Fig. 2B in the main text. Patients are sorted from lowest median seizure dissimilarity (patient 934) to highest median seizure dissimilarity (I002 P006 D01). The red arrow indicates patient 931, the example patient from the main text. Each point corresponds to the dissimilarity of a pair of seizures; see Fig. 2A of the main text for an additional visualisation of how dissimilarity matrices were visualised as distributions. Each patient's distribution is coloured based on whether the seizures are best described as a spectrum of pathways (teal) or multiple clusters (i.e., separable groupings) of seizure pathways (purple). Note that in patients with multiple clusters, the distribution of seizure dissimilarities tends to be bimodal since most pairs of seizures are either fairly similar (with low dissimilarity) because they are in the same cluster or fairly different (with high dissimilarity) because they are in different clusters. Meanwhile, patients without multiple seizure clusters tend to have more intermediate seizure dissimilarity values. This difference in distributions is apparent for patients Study 019 (spectrum of seizures) and 1167 (two seizure clusters), although it is less clear for some patients with weaker clusters and/or fewer seizures. B) The number of patients with different numbers of seizure clusters based on seizure dissimilarities. The "spectrum" case corresponds to one seizure cluster (which still contains variability in seizure pathways). The majority of patients had a spectrum of seizure pathways. C) Distribution of median seizure dissimilarities in patients with a spectrum of seizures (left, teal) or multiple seizure clusters (right, purple).

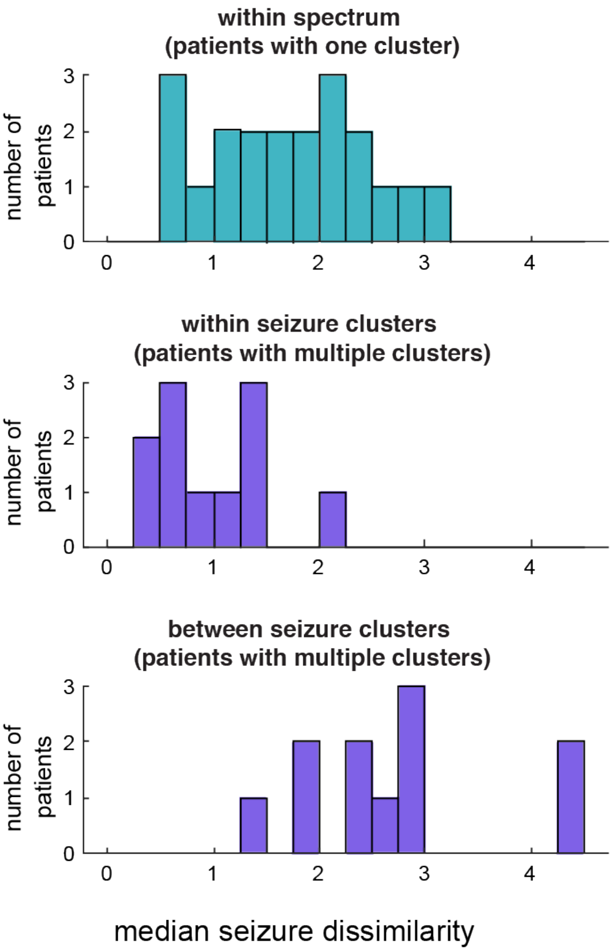

Fig. S6.2: Amount of variability within and between seizure clusters. Teal correspond to data from patients with a spectrum of seizures (i.e., single seizure cluster), while purple corresponds to data from patients with two or more seizure clusters. Top histogram: median within-cluster seizure dissimilarity in patients with a spectrum of seizures (one seizure cluster). Note that since all seizures are in the same cluster in these patients, these values are the same as the overall median seizure dissimilarity of these patients. Thus, this histogram reproduces the teal histogram shown in Fig. S6.1C and again demonstrates that there is variability in seizure pathways in these patients, even though seizures cannot be grouped based on differences in their pathways. Middle histogram: median within-cluster seizure dissimilarity in patients with multiple seizures clusters. Bottom histogram: median between-cluster seizure dissimilarity in patients with multiple seizure clusters.

Text S7: Seizure variability is not driven by differences in seizure clinical type

In this section, we examine of the relationship between our measure of seizure dissimilarity (which captures differences in within-patient seizure network evolutions) and the International League Against Epilepsy (ILAE) clinical seizure classification (6–8). Importantly, unlike our seizure dissimilarity measure, the ILAE clinical seizure classification was not designed to group seizures based on their iEEG dynamics; rather, a type is defined as "a useful grouping of seizure characteristics for purposes of communication in clinical care, teaching, and research (8)." Nonetheless, comparing the seizure groupings based on dynamics and clinical classification reveals if the observed variability is solely explained by differences in seizure clinical classification; for example, it is possible that the observed variability could be solely attributed to focal seizures that all have similar initial dynamics, but sometimes secondarily generalise.

Based on the clinical reports for each patient, seizures were labelled as

- subclinical: seizures that are visible electrographically, but do not cause any apparent symptoms
- focal: seizures that originate in networks within one hemisphere; here, we limit this label to seizures that also remain focal (i.e., do not secondarily generalise)
- secondarily generalised: seizures that begin focally and subsequently engage bilateral networks, resulting in convulsions that have tonic and/or clonic components

Note that secondarily generalised seizures now correspond to "focal to bilateral tonic clonic seizures" in the more recent ILAE clinical classification(8); however, to be consistent with the terminology in the patients' reports and the previous literature, we use the older classification term here. Additionally, focal seizures can be further subdivided into different categories based on, for example, whether awareness is preserved during the seizure. However, we do not make those designations here due to the absence of this information or uncertainty in the classification.

Comparison of clinical types to seizure clustering based on network evolutions

We first qualitatively examined whether groupings of seizures based on their network evolutions were associated with their relative clinical type assignments. The dendrograms of Fig. S7.1 show the hierarchical clustering of seizures, based on seizure network evolutions (i.e., based on our seizure dissimilarity measure) in example patients. More similar seizures, represented by leaves on the dendrogram, are joined by nodes. The height of the node linking two seizures (or groups of seizures) represents the dissimilarity between them, with higher nodes indicating less similar seizures. The seizures are labelled by their ILAE clinical type (subclinical, focal, or secondarily generalised) to examine the relationships between seizures of the same and different clinical type(s).

In some patients, only a single clinical seizure type was available/suitable for analysis. Fig. S7.1a provides examples of three patients in which only focal seizures were analysed. Although all seizures shared the same clinical type, there was variability in the seizure network evolutions in each patient. For example, based on the dendrogram of patient 1005, seizures 13, 14, and 15 appeared to have similar evolutions, but overall this group was different from the remaining seizures. **These patients alone demonstrate that the observed variability is not solely due to differences in clinical seizure type, as there is variability within a single clinical type.** The variability within focal seizures is unsurprising, as focal seizures can have varying levels of spread and symptoms, indicating diversity in their spatial spreads and severity. Our hierarchical clustering based on seizure evolution also suggests that focal seizures can also be associated with different patterns of brain network interactions.

In other patients, we saw that, based on their network evolutions, seizures could be divided into groups that correspond to their clinical types (Fig. S7.1b). In patients 821 and 1163, the dendrograms can be cut such that the resulting clusters match the ILAE clinical classification: each cluster only contains one clinical type, and each cluster also contains all examples of the clinical type. However, note that this grouping of the seizures is not necessarily the *optimal* non-hierarchical clustering of the seizures (see SI Appendix, Text S6 for how optimal clusters were determined using the gap statistic). Indeed, even in these patients, we observed that high levels of variability within some clinical types. For example, in patient 1163, there were large amounts of variability within the focal seizures. **Thus, even in the patients with close agreement between clinical types and clusters based on seizure evolution, there was additional variability in seizure network evolutions that was not explained by the coarse clinical classification alone.**

Finally, in other patients, we observed that the seizures' clinical classifications do not perfectly align with the hierarchical clustering based on the seizures' network evolutions (Fig. S7.1c). In these patients, cutting the dendrograms at different levels produced clusters that contain multiple clinical types and/or more than one cluster containing a given clinical type. For example, partitioning patient I002_P006_D01's seizures to produce four clusters would segregate the secondarily generalised seizures and subclinical seizures, as these types have fairly homogeneous evolutions in the observed seizures. However, the two focal seizures, 3 and 7, would form separate groups due to their disparate dynamics. Meanwhile, in other patients, we saw that seizures can be more similar to seizures of different clinical types than seizures of the same clinical type. For example, in patient 1200, seizure 4 (focal) was grouped with subclinical seizures, seizures 5, 6, and 7, while seizure 9 (also focal) appeared to be very similar to a different subclinical seizure, seizure 11. Similarities across clinical types is also unsurprising, as seizures of different clinical types can share similar properties, such as ictal rhythms and propagation pathways. **Our analysis suggests that similar brain network interactions can also occur in seizures of different clinical types.** Notably, the similarities between focal and subclinical seizures also suggests that seizures can share common network features even when their symptoms differ.

In summary, although seizures of the same clinical type often shared similar network evolutions, we also observed that, in a given patient, 1) there was variability in seizure network evolutions within seizures of the same clinical type, and 2) seizures of different clinical types could share aspects of their network dynamics. These results are unsurprising given that the coarse clinical classification used here is not designed to group seizures based on their network dynamics. Indeed, more generally, seizures of the same clinical type are known to have different features, while seizures of different clinical types can have similar dynamics.

Distributions of seizure dissimilarities

To quantitatively examine the relationship between seizure clinical type and seizure dissimilarities, we also examined the distributions of seizure dissimilarities between seizures of the same or different clinical type(s) (Fig. S7.2). To create these distributions, we sorted the seizure dissimilarities of seizures with known clinical types based on the clinical type(s) of each compared pair of seizures. Note that these distributions contain seizure dissimilarities from multiple patients; however, each individual seizure dissimilarity value corresponds to a pair of seizures from within the same patient.

As expected, the median seizure dissimilarities of seizures within the same clinical type (e.g., focal vs. focal seizures) were lower than the median seizure dissimilarities of seizures from different clinical types (e.g., focal vs. secondarily generalised seizures). However, within each of the within-

type distributions, there was a wide range of dissimilarities: although seizures of the same type often had a low dissimilarity (indicating similar network evolutions), many pairs also had a relatively high dissimilarity (indicating different network evolutions). Meanwhile, across seizure types, there were also be pairs of seizures with relatively low dissimilarities, especially when comparing focal and subclinical seizures. Secondarily generalised seizures, on the other hand, tended to differ from seizures of different clinical types, although they sometimes shared some similarity with focal seizures (dissimilarity values ~2). As such, our measure of seizure dissimilarity was somewhat consistent with seizure clinical types in that seizures of the same type tended to be more similar that seizures of different types. However, as expected, there were also large amounts of variability within types that was unexplained by the clinical classification alone, and seizures across clinical types could have more similar network evolutions than seizures of the same clinical type.

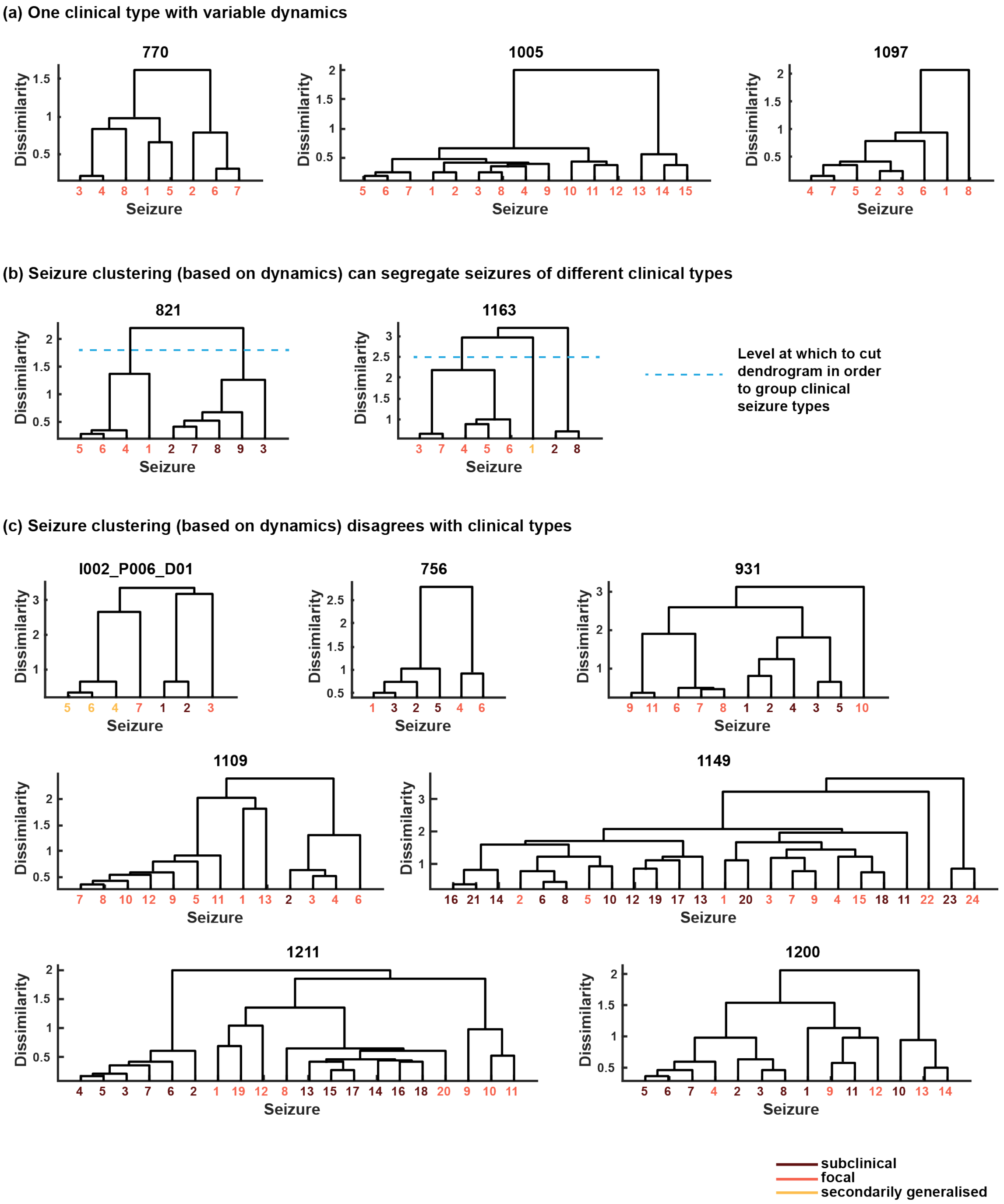

Fig. S7.1: Comparison of ILAE clinical seizure types classification and variability in seizure network evolutions. (a-c) Dendrograms of example patients with known clinical classifications for all seizures. Each dendrogram describes the hierarchical clustering of the patient's seizures, based on the seizure network evolutions (see SI Appendix, Text S6). More similar seizures, represented by leaves on the dendrogram, are joined by nodes. The height of the node linking two seizures (or groups of seizures) represents the dissimilarity between them, with higher nodes indicating less similar seizures. Seizure labels are coloured by their ILAE clinical type (dark red = subclinical, orange = focal, yellow = secondarily generalised). (a) Example patients whose analysed seizures consisted of a single clinical type (focal). Note that there was variability in seizure network evolutions in each patient. (b) Example patients whose analysed seizures consisted

of at least two clinical types, and whose hierarchical clustering of seizures agreed with the clinical seizure classification; i.e., the dendrogram can be cut at a specific level (blue dotted line) to perfectly segregate seizures of different clinical types. (c) Example patients whose hierarchical clustering did not agree with the seizure clinical classification; i.e., there is no way to cut the dendrogram to perfectly segregate seizures of different clinical types. The resulting clusters will contain multiple clinical types and/or multiple clusters will contain the same clinical type.

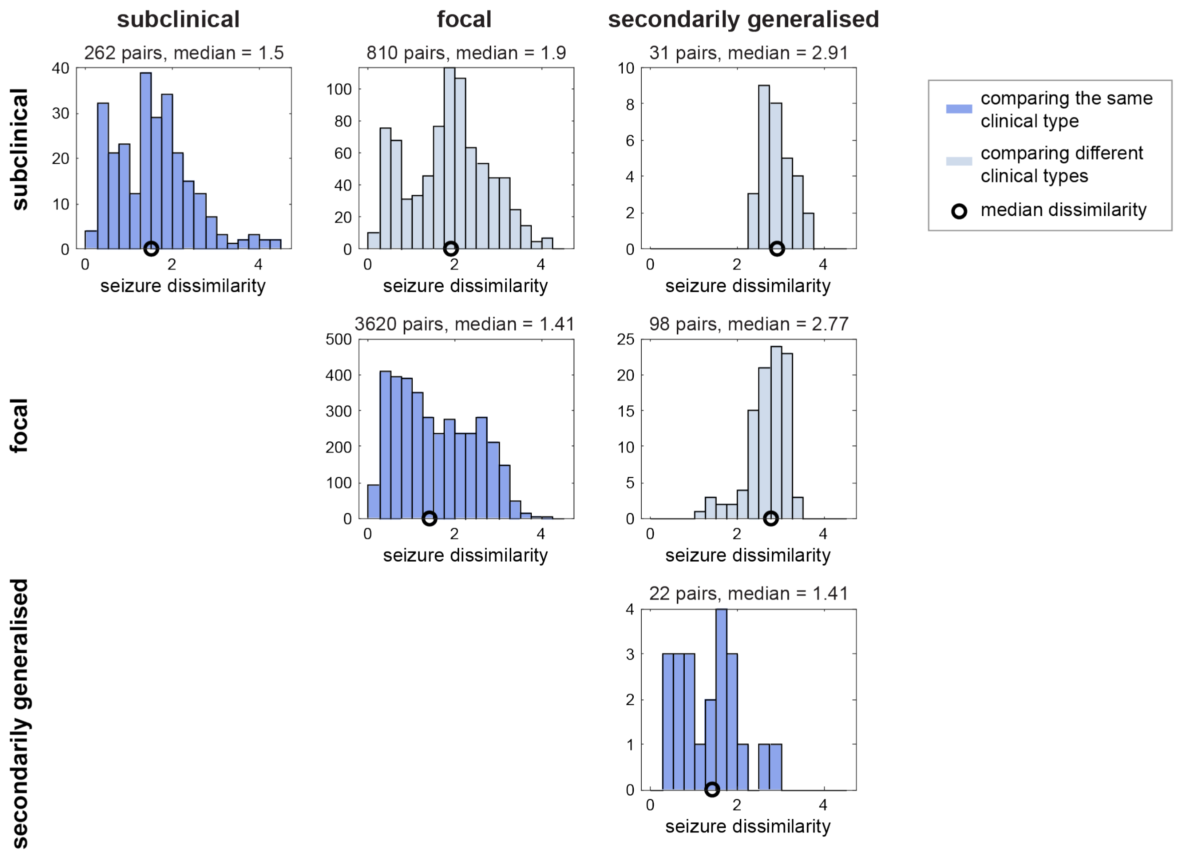

Fig. S7.2: Distributions of seizure dissimilarities between seizures of the same and different clinical types. Each distribution contains seizure dissimilarities (computed between pairs of seizures from the same patient) of pairs of seizures that have the specified clinical type(s). Row and column labels indicate the clinical types compared in each distribution; for example, the row 1, column 1 distribution contains comparisons of subclinical seizures to other subclinical seizures, while the row 1, column 2 distribution contains comparisons of subclinical seizures to focal seizures. The number of pairs of seizures in each distribution is noted above each plot, along with the distribution's median dissimilarity (also marked with a black circle on each plot). The median dissimilarities of comparisons within seizure types (diagonal plots, in blue) are lower than the median dissimilarities of comparisons across seizure types (off-diagonal plots, in light grey); however, there is also variability in each category of comparisons.

Text S8: No relationship between features of seizure variability and clinical measures

In this section, we explore if certain features of seizure variability are associated with clinical features, such as seizure onset location or surgical outcome.

Comparison of clinical features to temporal distance correlation, number of clusters, and median seizure dissimilarity

For each patient, we first computed three different features that describe aspects of seizure variability:

1. Temporal distance correlation: the correlation between seizure dissimilarities and the amount of time elapsed between seizures
2. Number of clusters: the optimal number of seizures clusters, based on seizure network evolutions (i.e., based on the seizure dissimilarity matrix)
3. Median seizure dissimilarity: the average seizure dissimilarity across all pairs of seizures (computed by taking the median of the upper triangular elements of the seizure dissimilarity matrix)

See Methods of the main text and SI Appendix, Text S6 for details on computing these measures.

For each measure, we compared

- Patients labelled as having strictly temporal lobe onset seizures (n = 12) vs. strictly frontal lobe onset seizures (n = 8)
- Patients labelled as having left hemisphere onset seizures (n = 15) vs. right hemisphere onset seizures (n = 13)
- Male patients (n = 16) vs female patients (n = 15)

We were unable to compare seizure variability features in bilateral onset patients or in patients with certain pathological features (e.g., hippocampal sclerosis) due to small sample sizes.

For each group and feature, we first used the Kolmogorov Smirnov test to determine if the group's feature distribution was consistent with a normal distribution. If so, we also used a two-sample F-test to test the null hypothesis that the feature distributions of two groups being compared (e.g., temporal and frontal patients) had equal variances. For these pairs of distributions, we failed to reject the null hypothesis in all cases, allowing us to assume equal variances and compare the group means using a two-tailed Student's t-test. If, however, the normality assumption was not satisfied, a Wilcoxon rank sum test was instead used to compare the group medians.

Additionally, we used Spearman's correlation to evaluate the relationship between the surgical outcome (scored according to ILAE criteria) and each of the three features in the 26 patients who underwent surgical resection and had a known surgical outcome (see Table S1 for the ILAE surgical outcome of each patient). An ILAE score of 1 indicates complete seizure freedom after surgery, with successively higher scores indicating worse outcomes.

For all features, we found no significant differences (defined as a p-value < 0.05) between temporal and frontal lobe patients, left and right hemisphere onset patients, or male and female patients (Fig. S8A-C). Additionally, there was no significant relationship between ILAE surgical outcome and any of the seizure variability features (Fig. S8D). Our results suggest that these clinical features do not impact the amount or form of seizure variability, or that any effect is smaller than detectable

with our sample sizes. Further research is needed to determine the factors that shape features of seizure variability.

Comparison to model timescales (see main text, "Seizure pathways change on different timescales")

We additionally investigated whether ILAE surgical outcome differed between patients with different categories of seizure variability (linear, circadian, or linear + circadian). For the 22 patients with both an assigned model category (i.e., their model category was not "other/indeterminate") and a known ILAE surgical outcome, we used ordinal regression to test for a significant relationship between ILAE surgical outcome and model type. The two independent variables were whether the patient's model had a linear component (i.e., if the patient belonged to either the linear or linear+circadian category) and whether the patient's model had a circadian component (i.e., if the patient belonged to either the circadian or linear+circadian category). There was no significant relationship between these variables and ILAE surgical outcome ($p = 0.35$ and $p = 0.84$ for the linear component and circadian component predictors, respectively).

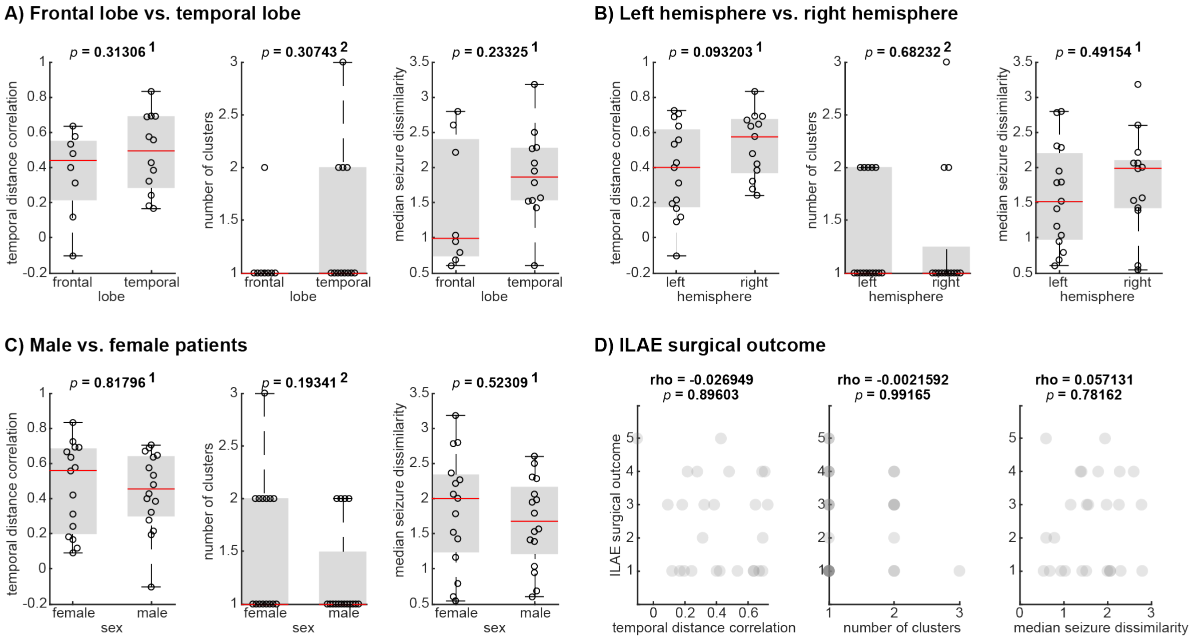

Fig. S8: Comparison of seizure variability features in different groups of focal epilepsy patients. A) Comparison of features in patients with frontal lobe onset vs. patients with temporal lobe onset. B) Comparison of features in patients with left hemisphere onset vs. right hemisphere onset. C) Comparison of features in male vs. female patients. D) Features vs. patient ILAE surgical outcome. In A, B, and C, each subplot shows the distribution of a seizure variability feature in the corresponding patient subgroups. See SI Appendix, Text S8 for a description of each feature. Red horizontal lines mark the median feature value for each population, while the lower and upper bounds of each grey box indicate the first and third quartiles, respectively, of each distribution. For each feature, an appropriate test (superscript 1 = two-tailed Student's t-test, 2 = Wilcoxon rank sum test; see Text S8 for details) was used to compare group means or medians, yielding the given *p*-values. In D, each scatterplot shows the relationship between a seizure variability feature and patient ILAE surgical outcome. Note that in some plots, points overlap, especially when the seizure variability feature only takes on discrete values (e.g., the number of seizure clusters); in these cases, the darkness of the point indicates the number of patients it represents. The association between surgical outcome and each feature was quantified using Spearman's correlation, and the resulting correlation rho and associated *p*-value is given above each scatter plot.

S9: No relationship between temporal patterns of seizure dissimilarities and AED reduction

Comparison to overall correlation with temporal distances (see main text, "Seizures with more similar pathways tend to occur closer together in time")

During presurgical recordings, the antiepileptic drug (AED) dosages of patients are often gradually reduced to provoke more seizures, and thus improve localization of the epileptogenic zone(2, 9–11). Because such medication alters neural excitability(12, 13), it is possible that it also affects seizure dynamics. Thus, gradual changes in AED dosages could potentially produce gradual changes in seizure evolution, leading to the observed temporal changes in seizure pathways in many patients. We therefore investigated if differences in the correlation between seizure dissimilarities and temporal distances across patients could be attributed to whether antiepileptic medications were reduced in each patient.

Information about medication dosages was available from the clinical reports of nineteen patients. This information was extracted and used to label each patient as "AED reduction performed" or "no AED reduction performed." Medication changes due to stat doses of medication (i.e., medication given in addition to the planned dose in order to control seizures) were not considered in this assessment; rather, we sought to identify whether gradual changes in medication were intentionally made, as those could underlie the observed gradual temporal changes in seizure pathways. We then also labelled whether each of the 19 patients had a significant or not significant correlation between seizure dissimilarities and temporal distances.

Table S9.1 shows the cross-tabulation table of these variables. If AED reduction reliably altered seizure evolution, we would expect significant correlations between seizure dissimilarities and temporal distances in approximately all patients who underwent medication reduction. However, of the eleven patients that underwent AED reduction, four did *not* have a significant association between these distances. Additionally, if the temporal changes in seizure pathways were *solely* due to medication reduction, we would not expect a significant relationship between seizure dissimilarities and temporal distances in patients who did *not* undergo AED reduction. Instead, we observed a significant correlation in five of the eight patients who did not undergo AED reduction. A χ^2 test found no association between whether AED reduction was performed and whether the correlation between seizure dissimilarities and temporal distances was significant ($\chi^2 = 0.0026$, $p = 0.96$). As such, the temporal relationship between similar seizure pathways cannot solely be attributed to AED reduction in our cohort.

Table S9.1: Cross-tabulation table describing the frequency of antiepileptic drug (AED) reduction (columns, "AED reduction performed") and the frequency of a significant correlation between temporal distances and seizure dissimilarities (rows, "Significant correlation") in our cohort of patients. For example, three patients did not undergo AED reduction and did not have a significant correlation between temporal distance and seizure dissimilarities (AED reduction performed: no, significant correlation: no). At the end of each row and column, the total count of patients in the respective category is also given (e.g., a total of 7 patients did not have a significant correlation between temporal distance and seizure dissimilarities).

		AED reduction performed		
		**no**	**yes**	**total**
**Significant correlation**	**no**	3	4	7
	**yes**	5	7	12
	**total**	8	11	**19**

Comparison to model timescales (see main text, "Seizure pathways change on different timescales")

We additionally explored if patients who underwent AED reduction had seizure variability that was best described by a particular model (linear, circadian, or linear + circadian changes in dynamics). As a reminder, the linear model describes more gradual changes in seizure pathways over the course of the recording, which could be attributed to AED reduction.

Table S9.2 shows the number of patients with and without AED reduction who were assigned to each model category. A χ^2 test cannot be performed here due to small sample sizes and assignment to multiple categories (patients with linear + circadian variability could be considered members of both the linear and circadian groups). However, from the cross-tabulation table alone, it is apparent that similar proportions of patients with and without AED reduction were assigned to each model category. In particular, AED reduction alone cannot explain the linear pattern of seizure variability: six of the eight patients without AED reduction had variability that was categorised as either linear or linear + circadian. Thus, factors beyond AED reduction influence the temporal patterns of changes in seizure pathways in this cohort.

Table S9.2: Cross-tabulation table describing the frequency (number of patients) of each model category (linear, circadian, linear + circadian, and other/indeterminate) among patients with and without AED reduction.

	**Linear**	**Circadian**	**Linear + circadian**	**Other/indeterminate**
**AED reduction**	5	1	4	1
**No AED reduction**	4	2	2	0

Text S10: Supplementary modelling details and results

Model parameter scan

Table S10: Scan values for model parameters

Parameter	Description	Scan range	Scan step size
l	Scales linear contribution (i.e., slow/gradual changes)	0 to 1	1
c	Scales circadian (sinusoidal) contribution	0 to 5	0.1
n	Scales noise contribution	0.025 to 0.5	0.025

Table S10 shows the values used to scan the parameters l, c, and n in the model of seizure temporal correlation patterns (see main text Results, "Seizure pathways change on different timescales"). As discussed in the main text Methods (see "Modelling seizure dissimilarities and temporal correlation patterns"), the parameters l, c, and n control the relative contributions of the linear, circadian, and noise functions to the simulated temporal changes in seizure pathways. To reduce the model complexity, we do not change the phase of the circadian cycle; however, in future work, the circadian phase could also be included as an additional parameter. Alternatively, the parameter could be tuned based on the patient's known or likely sleep/wake cycle (data that was unavailable for our patients).

Note that temporal correlation patterns (see main text Methods, "Computing temporal correlation patterns") only depend on the relative magnitude of seizure dissimilarities, and not their absolute magnitude. This situation arises because only the order of dissimilarities affects Spearman's correlation between the seizure dissimilarities and temporal distances. Thus, since model parameters were selected based on how well they reproduced each patient's observed temporal correlation patterns, only the relative values of the model parameters matter for model selection. For example, given the same noise realisation, the temporal correlation patterns of the parameter sets (l = 0.5, c = 1, n = 0.05) and (l = 1, c = 2, n = 0.1) would be equivalent. These properties allowed us to limit the parameter scan, removing redundant parameter sets, in the following ways:

- First, we limited the values of l to 0 (no linear contribution) or 1 (linear contribution), and only scanned the values of c and n, relative to these fixed linear contributions. Therefore, for all model including a linear component, l = 1, and the values of the other parameters indicate the relative contribution of the linear component.
- Further, for parameter sets where l = 0, we likewise fixed the value of c to 1 and only scanned n to determine the relative contributions of the circadian and noise processes. Thus, for any patient whose pattern of dynamics was categorised as "circadian" (with no linear contribution), l = 0 and c = 1 for the model parameters.

Effect of level of noise on temporal correlation patterns

For the example models shown in the main text, Fig. 4B, we visualise how increasing the level of noise alters the temporal correlation pattern (Fig. S10.1). In general, increasing the level of noise attenuates the correlations produced by the linear and/or circadian dynamics. Additionally, noisy dynamics can produce random fluctuations in the correlation pattern (i.e.,

correlations that are much higher or lower than expected based on linear and/or circadian dynamics).

Modelling results

As a reminder, at each set of parameters, seizure dissimilarities and the corresponding temporal correlation patterns were simulated 1000 times. Each simulated temporal correlation pattern was then compared to the patient's observed temporal correlation pattern by computing the mean squared error (MSE) between the simulated and observed patterns. The likelihood L of a given parameter set was defined as the percentage of "good matches" (simulations with MSE ≤ 0.02185) produced by the 1000 noisy simulations at those parameter values. A model was termed a "linear model" if $c = 0$, a "circadian model" if $l = 0$, and a "linear + circadian model" if $l > 0$ and $c > 0$. For each class of model (linear, circadian, or linear + circadian), the model's likelihood (L_l, L_c, or L_{l+c}, respectively) was the highest likelihood among the set of qualifying parameter sets, and the "best model" was the model with the highest likelihood, L_{max}. L_n was also defined as the highest likelihood of the parameter sets without any linear or circadian contributions ($l = 0$, $c = 0$, $n > 0$).

However, this "best model" was only the "selected" model for the patient if

1) The best model clearly outperformed noise alone ($L_{max} \geq 2L_n$); otherwise, the patient's dynamics were classified as other/indeterminate.
2) The performance of the linear model and circadian model were clearly distinguishable ($L_l \geq 2L_c$ if the linear model was best; $L_c \geq 2L_l$ if the circadian model was best); otherwise, the patient's dynamics were classified as other/indeterminate.
3) If the best model was linear + circadian, it clearly outperformed the two simpler models ($L_{l+c} \geq 2L_l$ and $L_{l+c} \geq 2L_c$); otherwise, the patient's dynamics were classified as the simpler model (if one simpler model performed comparably by this criterion) or as other/indeterminate (if both simpler models performed comparably).

Fig. S10.2 shows the final modelling results for each patient after these model selection criteria were applied. From the simulated temporal correlation patterns (Fig. S10.2B), it is apparent that our simple model can approximately reproduce the observed temporal correlation patterns (Fig. S10.2A). Fig. S10.2C provides the selected model parameters for each patient, which were chosen using the above criteria. Fig. S10.2D provides the likelihood of the selected model, and Fig. S10.2E shows the relative performances of the "best model," with L_{max}, compared to each of the model categories. The relative performance was computed as the likelihood of the best-performing model, L_{max}, divided by the likelihood of the given model category of model (see Fig. S10.2 caption for an example). Lower values indicate better performances compared to the best model; indeed, the relative performance of the best model is 1 because $L_{max}/L_{max} = 1$. These relative performances were used to select the final model parameters (shown in Fig. S10.2C) and corresponding model category. For example, in some patients (e.g., Study 012-2, second row from top), the linear + circadian model performed best ($L_{max} = L_{l+c}$), but the linear model was selected because it performed comparably: in this case, $L_{l+c}/L_l = 1.058$, indicating that almost as many simulations from the linear model provided a good match to the observed temporal correlation patterns.

To illustrate how the model likelihood was computed, Fig. S10.3 shows the 1000 simulations arising from the selected parameter sets of three patients, 931, 1005, and 1211, which were modelled using the linear, linear + circadian, and circadian models, respectively. The simulations are ordered from lowest to highest MSE and are divided by whether they fall under the MSE threshold for a "good match" to the observed temporal correlation pattern (shown in

Fig. S10.2A). Note the similarity of the "good matches" to the observed temporal correlation pattern of each patient. Note that the average MSE of the set of simulations would not necessarily be an appropriate evaluation for model selection because noisy fluctuations can dramatically alter the simulated temporal correlation pattern, especially if the number of analysed seizures was small. For example, for patient 931 (Fig. S10.3A), although some simulations provide a good match to the observed temporal correlation pattern, other simulations have a very high MSE. Our approach determines the "likelihood" of observing such good matches to the observed dynamics, without penalising the parameter set for also producing drastically different dynamics under different noise realisations.

Relationship between model likelihood and sample size

Ideally, a measure of model fit reflects the amount of confidence in the model, and this confidence should increase with a larger sample size (here, the number of seizures). The model fit may also reflect how consistently the observed dynamics match the model dynamics. To evaluate our measure of model fit (model likelihood), we therefore compared the model likelihood of each patient to the number of seizures analysed in each patient (Fig. S10.4). We additionally visualised how the amount of noise in the selected model (the model parameter n) was related to model likelihood.

From Fig. S10.4, it is apparent that model likelihood tended to increase as the number of seizures analysed also increased. Spearman's correlation between these two measures was 0.438. Thus, our measure of model fit was sensitive to the number of seizures analysed, and we had more confidence in our model when the sample size was larger.

Notably, three patients with a relatively low number of seizures (patients Study 027, I002 P006 D01, and 821) had high model likelihood despite their small sample sizes. In these cases, the model likelihood was still high because their pattern of seizure variability was very consistent with the linear and/or circadian changes predicted by the model; i.e., the noise contribution (controlled by the model parameter n) was low for all of these patients. The low n in turn meant that the selected model parameters created consistent simulated temporal correlation patterns that were all a good match for the observed dynamics. Therefore, even though the sample sizes for these patients was low, the model likelihood was high because the observed variability was highly consistent with the linear and/or circadian pattern of changes predicted by the model.

This relationship between model likelihood and the noise parameter n was only present for patients with low numbers of seizures. Patients with high numbers of seizures had high model likelihood regardless of the selected value of n. This situation arose because we compared observed and simulated *correlations*, rather than trying to predict specific fluctuations in seizure pathways:

- When a patient had a *low* number of seizures, noisy fluctuations in seizure pathways could produce spuriously high or low correlations between seizure dissimilarities and temporal distances. Because these high correlations were unexplained by the linear or circadian trends in seizure dynamics, the model instead reproduced them by introducing higher levels of noisy dynamics. However, only a small number of these noisy simulations would reproduce the observed dynamics, resulting an overall low model likelihood (for e.g., see simulations of patient 931, with 11 seizures and $n = 0.2$, in Fig. S10.3A).
- In patients with a *high* number of seizures, spuriously high or low correlations become less likely to occur by chance. The same sample size effect would occur in the model, as well; due to the larger sample size, noisy fluctuations predictably attenuate the correlations created by other modelled changes. Thus, a high percentage of the noisy

model simulations would match the observed dynamics, as long as those dynamics were also consistent with other modelled trends (i.e., the linear and/or circadian dynamics) (for e.g., see simulations of patient 1211, with 20 seizures and $n = 0.275$, in Fig. S10.3C).

As such, model likelihood does not necessarily reflect whether the observed dynamics are consistent with the modelled linear/circadian changes in seizure pathways. Instead, such model uncertainty is directly incorporated into the model via the addition of noise. A higher n reflects that additional changes in seizure pathways occurred due to other factors or random fluctuations.

Note that the relationship between model likelihood and the number of seizures only holds if the model is able to reproduce the observed pattern of seizure variability. Fig. S10.2E compares the performances of the different types of models, and it is apparent that model likelihood will be low, regardless of the sample size, if the model cannot reproduce the observed temporal correlation pattern. Therefore, model likelihood does not purely reflect the number of seizures, but also whether the model is a good fit for the observed dynamics.

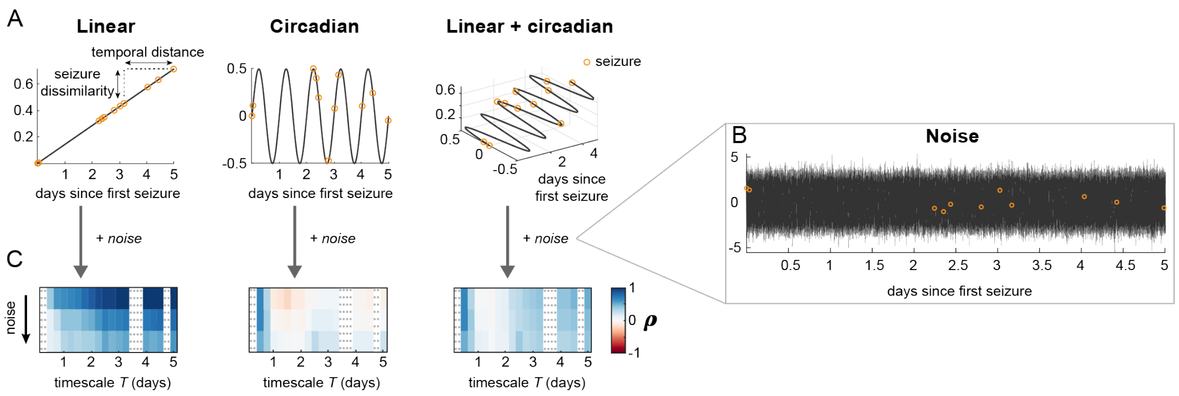

Fig. S10.1: Effect of noise level on example temporal correlation patterns. Using example models of patient 931's seizure variability (reproduced from Fig. 4B in the main text), Fig. S10.1 provides examples of how increasing the level of noise impacts temporal correlation patterns. A) As in Fig. 4B, seizure dissimilarities were modelled based on linear, circadian, or varying combinations of linear + circadian changes in seizure pathways. The simulated changes in seizure pathways are shown as different functions of time (left, top row), with patient 931's seizures marked in orange. In each model, distances between seizures reflected the observed temporal distances and the simulated seizure dissimilarities (see annotations on "Linear" model). B) In the same way, noisy changes in seizure dynamics were also incorporated into the models. The right panel shows the noisy dynamics (sampled from a standard Gaussian distribution) used in these example simulations; distances between seizures along the y-axis correspond to differences in seizure evolutions due to random fluctuations. C) The simulated seizure dissimilarities and temporal distances were compared across different timescales, yielding a temporal correlation pattern for each model (heatmaps, bottom row). For each model, the effects of three different levels of the additional, noisy dynamics are shown; specifically, from top to bottom of the heatmaps, the contribution of the noisy changes from Fig. S10.1B were multiplied by 0.1, 0.25, or 0.5 to vary their effect on the temporal correlation patterns. The top row of each heatmap is the temporal correlation pattern shown in Fig. 4B. Increasing the noisy contribution (second and third rows of the heatmaps) attenuates these correlations and/or produces additional fluctuations in the correlations.

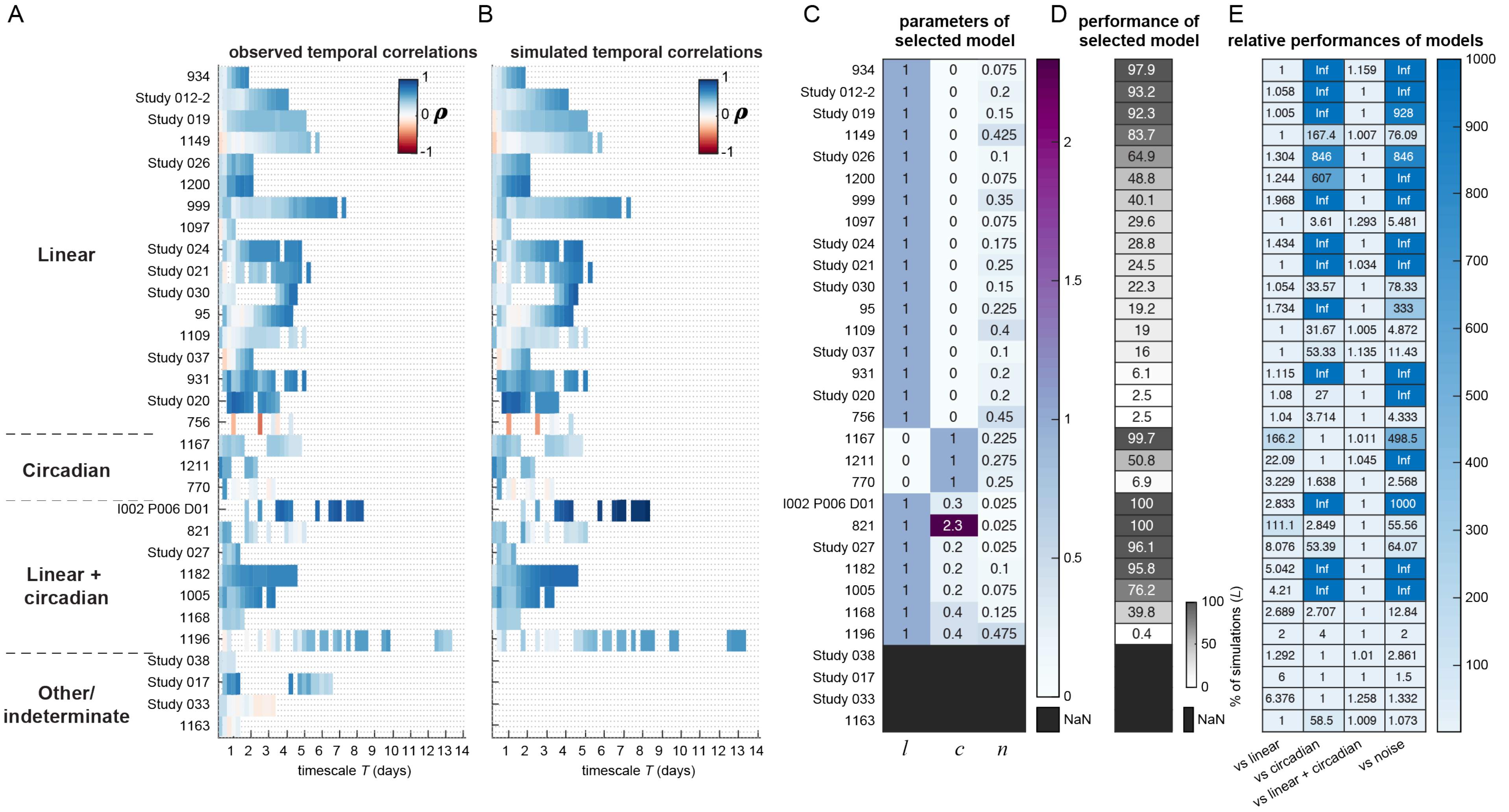
A
B
C
D
E
observed temporal correlations
simulated temporal correlations
parameters of selected model
performance of selected model
relative performances of models
Linear
Circadian
Linear + circadian
Other/ indeterminate
934
Study 012-2
Study 019
1149
Study 026
1200
999
1097
Study 024
Study 021
Study 030
95
1109
Study 037
931
Study 020
756
1167
1211
770
I002 P006 D01
821
Study 027
1182
1005
1168
1196
Study 038
Study 017
Study 033
1163
timescale T (days)
1 2 3 4 5 6 7 8 9 10 11 12 13 14
ρ
1
0
-1
l
c
n
1 0 0.075
1 0 0.2
1 0 0.15
1 0 0.425
1 0 0.1
1 0 0.075
1 0 0.35
1 0 0.075
1 0 0.175
1 0 0.25
1 0 0.15
1 0 0.225
1 0 0.4
1 0 0.1
1 0 0.2
1 0 0.2
1 0 0.45
0 1 0.225
0 1 0.275
0 1 0.25
1 0.3 0.025
1 2.3 0.025
1 0.2 0.025
1 0.2 0.1
1 0.2 0.075
1 0.4 0.125
1 0.4 0.475
2
1.5
1
0.5
0
NaN
97.9
93.2
92.3
83.7
64.9
48.8
40.1
29.6
28.8
24.5
22.3
19.2
19
16
6.1
2.5
2.5
99.7
50.8
6.9
100
100
96.1
95.8
76.2
39.8
0.4
100
50
0
% of simulations (L)
NaN
1 Inf 1.159 Inf
1.058 Inf 1 Inf
1.005 Inf 1 928
1 167.4 1.007 76.09
1.304 846 1 846
1.244 607 1 Inf
1.968 Inf 1 Inf
1 3.61 1.293 5.481
1.434 Inf 1 Inf
1 Inf 1.034 Inf
1.054 33.57 1 78.33
1.734 Inf 1 333
1 31.67 1.005 4.872
1 53.33 1.135 11.43
1.115 Inf 1 Inf
1.08 27 1 Inf
1.04 3.714 1 4.333
166.2 1 1.011 498.5
22.09 1 1.045 Inf
3.229 1.638 1 2.568
2.833 Inf 1 1000
111.1 2.849 1 55.56
8.076 53.39 1 64.07
5.042 Inf 1 Inf
4.21 Inf 1 Inf
2.689 2.707 1 12.84
2 4 1 2
1.292 1 1.01 2.861
6 1 1 1.5
6.376 1 1.258 1.332
1 58.5 1.009 1.073
vs linear
vs circadian
vs linear + circadian
vs noise
1000
900
800
700
600
500
400
300
200
100

Fig. S10.2: Modelling results for each patient. A) Observed temporal correlation patterns of all patients, sorted by the selected model (linear, circadian, linear + circadian, or other/indeterminate). Note that these results are also included in Fig. 4C of the main text. B) For each patient, the simulated temporal correlation pattern, arising from each patient's selected model parameters (see Fig. S10.2C), that best matched the observed temporal correlation pattern (i.e., the pattern that had the lowest MSE across the 1000 simulations produced by the selected parameter values). No simulated pattern is shown for the last four patients because none of the models (linear, circadian, or linear + circadian) met the model selection criteria. C) Parameter values of the selected model for each patient, with *l* controlling the linear contribution, *c* controlling the circadian contribution, and *n* controlling the amount of noise added to the simulated changes in seizure pathways. D) The performance of the selected model, as defined by the likelihood *L* of the selected model. The likelihood L is the percentage of simulations, created from different noise realisations using the selected model parameter values, that have a MSE ≤ 0.02185 to the patient's temporal correlation pattern. A higher likelihood indicates that the model is more likely to produce the patient's observed temporal correlation pattern. E) The relative performances of the different model categories (linear, circadian, and linear + circadian, as well as the noise-only model) compared to the best-performing model. The relative performance is defined as the likelihood of the best-performing model, L_{max}, divided by the likelihood of the given type of model. Lower values indicate better performances compared to the best model. For example, for patient 934 (top row), the linear model performed best ($L_{max}/L_l = 1$ because $L_{max} = L_l$). None of the simulations arising from the circadian or noise models provided good matches to the observed temporal correlation pattern, so the relative performance of these models is positive infinity ($L_{max}/0 = +\infty$). Finally, the linear model performed 1.159 times better than the linear + circadian model. Although the likelihood of these two models was therefore similar, the simpler model (with $c = 0$) provided a more parsimonious explanation of the dynamics. For each patient, the relative model performances were used to determine the model that clearly outperformed the other categories of models, while also providing the most parsimonious explanation of the observed dynamics.

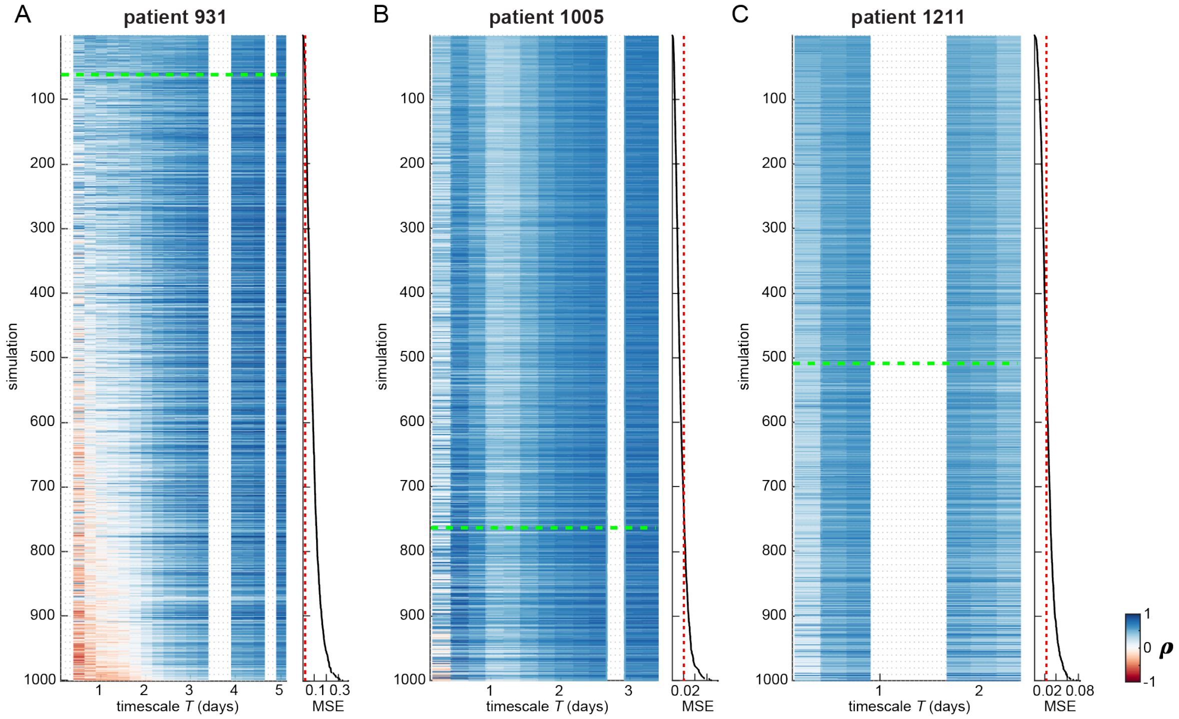

Fig. S10.3: All simulated temporal correlation patterns arising from the selected model parameters of three example patients: A) patient 931, B) patient 1005, C) patient 1211. See Fig. S10.2C for the parameters used to generate the simulated temporal correlation patterns. From left to right, the example patients were categorised as linear, linear + circadian, and circadian. For each patient, the heatmap shows the temporal correlation patterns, with the MSE of each temporal correlation pattern (compared to the patient's observed temporal correlation pattern) to the right of the heatmap. Simulations are ordered from lowest MSE (top) to highest MSE (bottom). The red dotted line shows the MSE threshold for a "good match" to the observed temporal correlation pattern, and the dotted green line marks the boundary of the simulations that fall under this threshold.

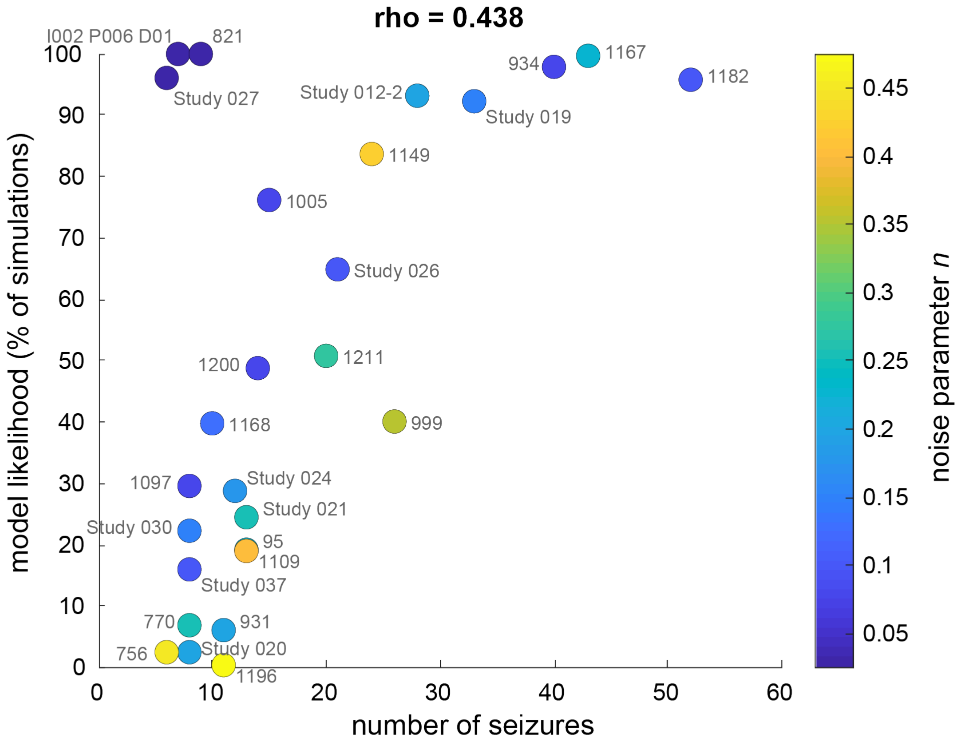

Fig. S10.4: Relationship between model likelihood, number of seizures, and the model noise parameter *n*. Scatter plot of model likelihood (the percentage of model simulations, arising from the selected model parameters, that matched the patient's observed temporal correlation pattern) vs. the number of seizures analysed in each patient. Spearman's correlation between these two measures is 0.438. Points are labelled by the corresponding patient ID and coloured by the value of the noise parameter, *n*, in the selected model. See Fig. S10.2 for precise values of model likelihood and *n* for each patient. Patients who were not assigned model parameters (patients Study 038, Study 017, Study 033, and 1163) are excluded from this figure.

Text S11: Dimensionality reduction using non-negative matrix factorization

Non-negative matrix factorisation (NMF) was used to reduce noise in each patient's connectivity matrix, *V*, in which each column corresponded to the functional connectivity of a seizure time window. NMF factored *V* into two non-negative matrices, *W* and *H*, such that $V \approx W \times H$. The matrix *W* contained patient-specific basis vectors, each of which had $6*(n^2 - n)/2$ features that captured a pattern of connectivity across all channels and frequency bands. Each original ictal time window was summarised as an additive combination of these basis vectors, with the coefficients matrix *H* giving the contribution of each basis vector to each time window.

To determine the optimal number of basis vectors, *r*, for each patient, the highest *r* that produced consistent sets of basis vectors was found (Fig. S10.1). This approach, known as stability NMF (14), exploits the non-deterministic nature of NMF to identify the *r* at which *W* consistently converges to a similar set of basis vectors. Since the resulting stable NMF basis vectors can be reliably found, they are thought to provide a meaningful representation of the data. To perform stability NMF for each patient, the value of *r* was scanned from 1 to 20. This scan range was chosen based on the observation that the stability of the factorisation greatly decreases at approximately $r>10$ in our data, and is consistent with the number of connectivity patterns typically found in ictal iEEG data in other studies (15–17). At each *r*, NMF of *V* was performed 25 times using the alternating nonnegative least squares with block principal pivoting method (18, 19). Each iteration used different random initializations of *W* and *H*, thus yielding 25 different factorizations of *V* at each value of *r*. Using the method established by Wu *et al.* (14), for each *r*, the instability *I* of two sets of basis vectors *W* and *W'* was defined as

$$I(r)_{W,W'} = \frac{1}{2r}\left(2r - \sum_{j=1}^{r} \max_{1\le i\le r} P_{ij} - \sum_{i=1}^{r} \max_{1\le j\le r} P_{ij}\right)$$

where *P* is the Pearson's cross-correlation matrix of the sets of basis vectors. Low values of *I* indicate that similar sets of basis vectors were found in the separate iterations; indeed, if the two sets of basis vectors are the same (minus reordering), then $I = 0$. The instability of all 25*(25-1)/2 pairs of basis vector sets was then averaged to produce $I_{avg}(r)$. The highest *r* with $I_{avg}(r) \le 0.005$ was selected for each patient, thus allowing small deviations between the observed basis vector sets, while still enforcing consistent factorisations across iterations. At this *r*, the factorisation yielding the lowest reconstruction error was used to construct $V^* = W \times H$, a lower-rank approximation of the original time-varying seizure functional connectivity. This noise-reduced version of the connectivity was used in the downstream analysis.

Note that the NMF factorisation can also be used to cluster seizure time windows into states (20, 21) (Fig. S11.2), and we use this approach to provide an alternative visualisation of seizure pathways on Zenodo (http://dx.doi.org/10.5281/zenodo.3692923).

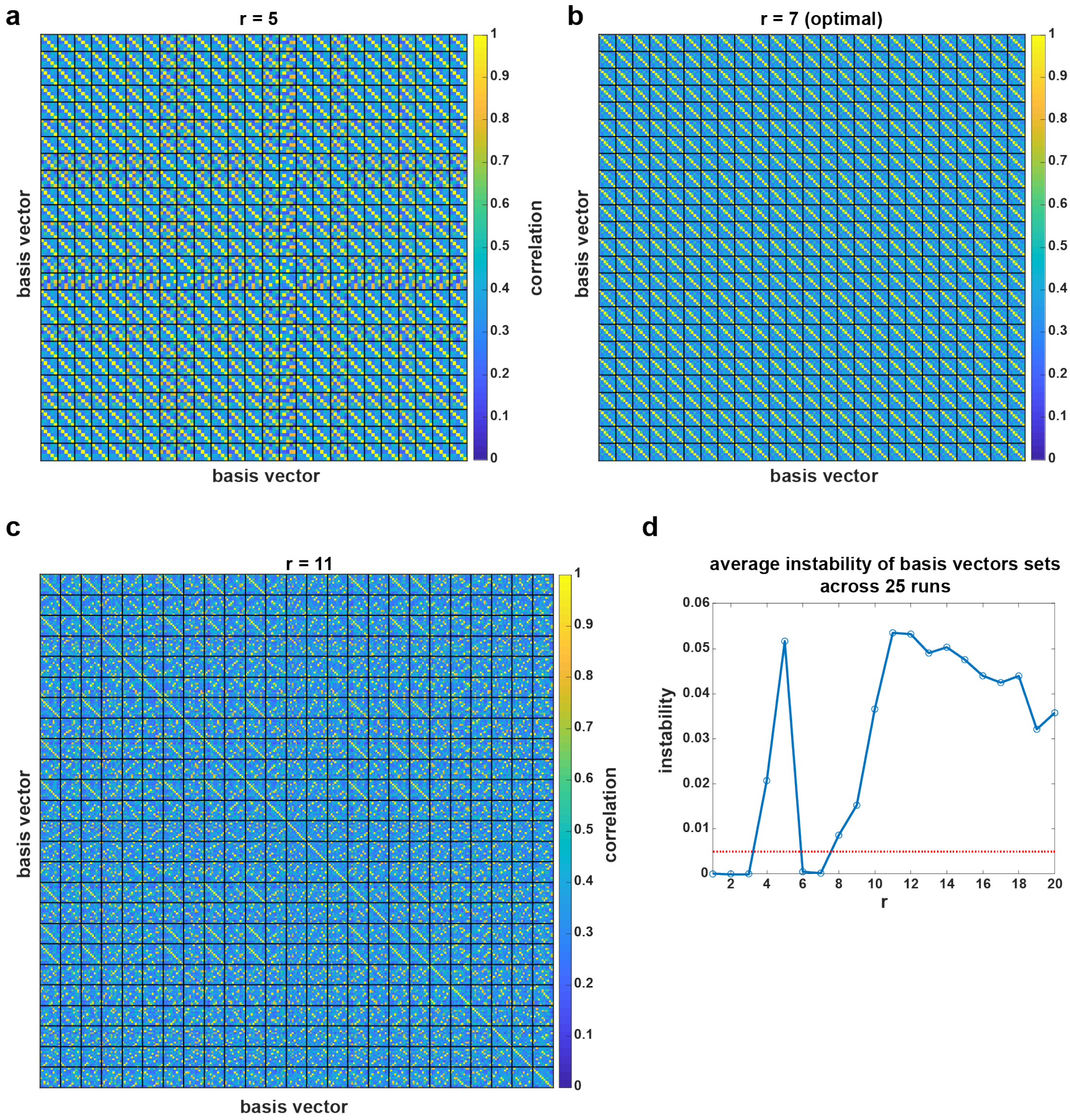

Fig. S11.1: Finding the optimal number of NMF basis vectors using stability NMF in an example patient, I002_P006_D01. The optimal number of states was 7 in this patient. At each number of basis vectors, NMF was repeated 25 times using different initialisations, yielding 25 sets of basis vectors. (a-c) Correlation matrices showing Pearson's correlation between all pairs of basis vectors found within and between different initialisations of the NMF algorithm. In each matrix, the same number of basis vectors were optimised for in each run: 5 (a), 7 (b), or 11 (c) basis vectors. Black lines mark the divisions between different runs of the algorithm. When possible, the basis vectors were re-ordered to emphasise similarity between different runs. Specifically, within run *i*, if there was a unique closest match to each of the basis vectors of run 1, the basis vectors of run *i* were re-ordered so that they were in the same order as the corresponding basis vectors in run 1. When r = 7 (b), note that similar sets of basis vectors were found across each run: in a given run, each basis vector had a high (close to 1) correlation to a basis vector of another run. Meanwhile, when r = 5 (a) or r = 11 (c), there was variability in the sets of basis vectors found across runs. (d) Plot of the instability, I, of the basis vector sets, averaged across all pairs of runs, vs. the number of basis vectors, r. For a given r, the average instability quantifies the dissimilarity in the basis vectors found across runs. A low average instability indicates that similar sets of basis

vectors were found regardless of the initialization of NMF algorithm; i.e., NMF converged to similar sets of basis vectors from different initial points in the search space. We defined the optimal number of basis vectors, r, as the highest r at which $I(r) < 0.005$. For this patient, the optimal number of basis vectors was $r = 7$.

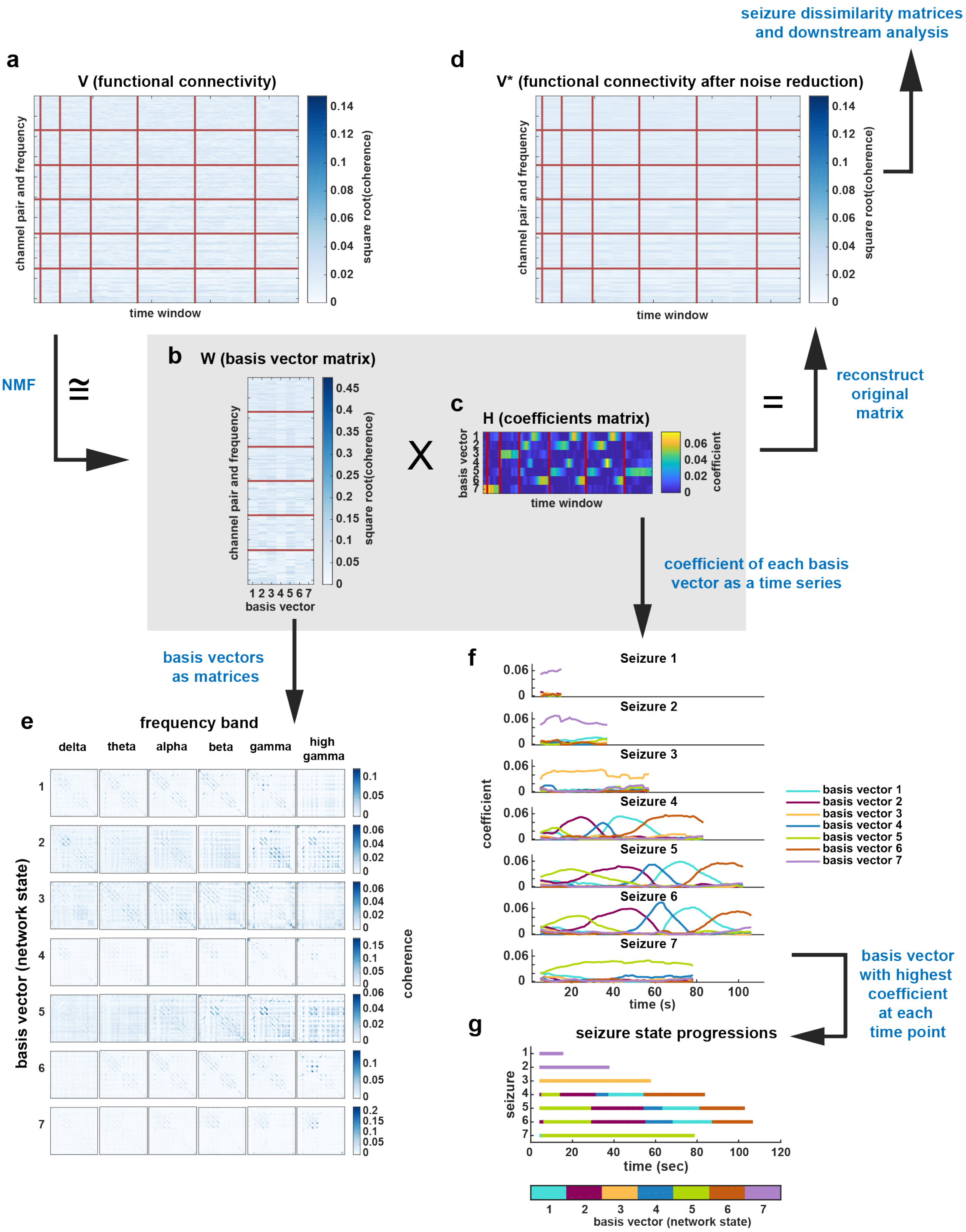

Fig. S11.2: Workflow for using NMF to reconstruct seizure functional connectivity and assign network states to each time point (alternative visualisation of seizure pathways). The factorisation of patient I002_P006_D01, which was chosen using stability NMF (see Fig. S11.1), is used as an example. From the matrix V (a), which contains the functional connectivity evolution of all seizures, NMF found a set of basis vectors, each of which forms a column in the matrix W (b), and a coefficients matrix, H (c), that describes the contribution of each basis vector to the observed connectivity at each time point. W and H were used to construct a noise-reduced version of the original matrix, which we call V* (d). In V, V*, and W, rows correspond to features

(here, the coherence between a given pair of channels at a given frequency band). The boundaries between features corresponding to different frequency bands are delineated using horizontal red lines (e.g., the top rows correspond to coherence in the delta frequency band). Note that the heatmap colouring corresponds to the square root of the coherence between pairs of channels to aid in visualising the structure of the data; however, the original coherence values were used for NMF and the downstream analysis. Meanwhile, the columns of V, V*, and H correspond to a seizure time window, with vertical red lines marking the boundaries between seizures. (e) Each NMF basis vector is a single column vector in W; however, each of the component frequency band matrices can be re-written in matrix form to emphasise the network pattern. Each basis vector therefore corresponds to a set of six connectivity matrices that describe the network configuration of all pairs of channels across the six frequency bands. Note the different colourbar scale for each basis vector. (f) The coefficients matrix H can be visualised as a time series of the coefficients for each basis vector. Each time point corresponds to the functional connectivity of a 10 s window of ictal iEEG and, at each time point, the coefficients indicate how much each basis vector contributes to the observed seizure connectivity. Note that at a given time, a single NMF basis vector usually has a much higher coefficient relative to the other basis vectors (i.e., there is sparsity in each column of the coefficients matrix, H). Thus, the dominant basis vector provides a simplified description of the network dynamics at that time point. (g) As an alternative visualisation of seizure pathways, each time point was assigned to a network state corresponding to the dominant NMF basis vector, resulting in a series of state progressions for each seizure. These visualisations are provided for each patient in the Zenodo submission.

S12: Comparison of seizure dissimilarity to metric distances

Seizure dissimilarity is a nonmetric measure

Our seizure dissimilarity measure is computed by using dynamic time warping (DTW) to align similar sections of seizure dynamics in a pair of seizures, and then taking the average distance between the warped time courses (see main text Methods and SI Appendix, Text S3). We wish to point out as a technical note that due to the warping step, the seizure dissimilarity measure is not a metric distance. Like a metric distance, all dissimilarities are non-negative, the dissimilarity of a seizure to itself is zero, and the dissimilarity between pairs of seizures is symmetric; however, the triangle inequality does not necessarily hold. In particular, any two seizures that follow approximately the same pathway will have a near-zero dissimilarity, regardless of their rates of progression along the pathway. However, their relationship to other seizures that share *part* of the same pathway will depend on how long (temporally) the seizures share the same pathway. Thus, although pairs of seizures may have a low dissimilarity, their relationships to other seizures may differ due to their different rates of progression. These situations can, in turn, lead to violations of the triangle inequality.

To illustrate this point, consider two seizures, A and B, that follow the same dynamical pathway, but at different rates; they will be considered virtually equivalent, and thus have a low seizure dissimilarity, due to the dynamic time warping step. However, their dissimilarity to a third seizure, C, that shares only *part* of the same pathway will differ, as their different rates of progression result in different relative durations of disparate dynamics in these comparisons. In such situations, violations of the triangle inequality may arise. These points are illustrated in the hypothetical dissimilarity matrix, D:

	A	B	C
A	0	0.5	3
B	0.5	0	5
C	3	5	0

Seizures A and B are very similar ($d_{AB} = 0.5$), but seizure A is less dissimilar (i.e., more similar) to C than seizure B is to C, a situation that can arise if seizures A and B progress at different rates. As such, $d_{AB} + d_{AC} = 0.5 + 3 = 3.5$, which is less than $d_{BC} = 5$, violating the triangle inequality.

Comparison to metric distances

To evaluate whether the nonmetric nature of seizure dissimilarities affects our analysis, we also compared the within-patient seizures of our cohort using two distance measures of curves/trajectories that are independent of the time series duration as well as the distance traversed by the trajectory in the feature space: the Fréchet distance and the Hausdorff distance. For clarity, we will refer to our original seizure dissimilarity measure as "DTW dissimilarities" in the remainder of this section.

The first alternative dissimilarity measure is the "Hausdorff distance". To compute this measure, the distances between all pairs of time points in the two time series are first calculated. Then, for every time point, the smallest distance to the other trajectory is found. The Hausdorff measure is then the maximum of all of these smallest distances.

Formally, the Hausdorff distance d_H is defined for two curves X and Y in a metric space (M,d) as:

$$d_{\mathrm{H}}(X,Y) = \max\{\,\sup_{x\in X}\inf_{y\in Y} d(x,y),\ \sup_{y\in Y}\inf_{x\in X} d(x,y)\,\}$$

Note that the Hausdorff distance is a metric distance and is independent of the trajectory length. It is also often applied generally to compare sets (i.e. not just trajectories).

Our second alternative dissimilarity measure is the Fréchet distance. The Fréchet distance is the minimum distance required to link a point travelling along one trajectory to a point travelling along another trajectory. This distance can be conceptualised as the smallest leash length required to walk a dog, given that the dog walker and dog respectively follow the two trajectories of interest. Unlike the Hausdorff distance, but like our DTW-based dissimilarity measure, the Fréchet distance respects the temporal order of progression along the trajectories. However, warping between the two trajectories is allowed in order to minimise the distance between them; i.e., the rate of travel along the trajectories is controlled in order to minimise the distance required to link them. In other words, part of the trajectory can be stretched, but entire sections cannot be repeated; in the mapping from the original trajectory to the warped trajectory, the trajectory indices must monotonically increase.

Formally, the discrete Fréchet distance(22) d_F is defined for two curves X and Y in a metric space (M,d) as:

$$d_F(X,Y) = \inf_{\alpha,\beta}\max_{t\in[0,1]}\{d(X(\alpha(t)),Y(\beta(t)))\}$$

Here, α and β are monotonic functions that map $t \in [0\ 1]$ to the start and end of the trajectories X and Y, respectively. The Fréchet distance, again, is a metric distance independent of the temporal durations of and distance traversed by the two trajectories.

Fig. S12.1A-C compares the three measures of seizure dissimilarity (DTW dissimilarity, Hausdorff distance, and Frechét distance) in our example patient from the main text, patient 931. The agreement between the different measures is visually apparent: all three dissimilarity/distance matrices have similar structures (Fig. S12.1A-B). There is also a high Spearman's correlation between DTW dissimilarities and each of the other measures (Fig. S12.1C), indicating that the relative orders of the seizure dissimilarities is mostly retained using the alternative measures. Notably, DTW dissimilarities tend to be smaller than Hausdorff and Frechét distances. This difference is unsurprising given that the Hausdorff and Frechét distances are both highly dependent on the largest distances between the two trajectories, while the DTW measure averages the distances across all warped timepoints. Thus, the DTW measure mitigates the effect of brief, large distances if the trajectories are otherwise similar. Across all patients, DTW dissimilarities also tend to have a high correlation with Hausdorff and Frechét distances (Fig. S12.1D), indicating that it captures similar information to these metric distances.

To explore the effects of the alternative measures on our main analysis, in each patient we repeated the comparison of temporal distances and seizure dissimilarities with the Hausdorff and Frechét distances (see main text Methods, "Comparison to temporal distances"). For each patient, this analysis resulted in a "temporal correlation" (correlation between temporal distances and seizure dissimilarities) for each measure. Fig. S12.1E shows scatter plots of the original temporal correlations vs. the temporal correlations computed using the alternative measures. Notably, the temporal correlations are relatively similar regardless of the dissimilarity measure; patients who originally had a positive temporal correlation still show the same relationship using the new measures. Thus, the observed temporal relationships between within-patient seizures are still

evident if a metric distance is used to compute seizure dissimilarities. As such, although our nonmetric DTW seizure dissimilarity measure must be used carefully as a substitute for a distance measure, we still find qualitatively similar results if we replace it with a metric distance.

Comparison to a metric approximation of seizure dissimilarities

Although results based on the Hausdorff and Frechét distances are similar to those using the DTW seizures dissimilarities, there will be some differences between these measures since they compare different aspects of the trajectories. In particular, compared to the DTW dissimilarities, Hausdorff and Frechét distances are more influenced by the largest distances between the two trajectories. It is therefore difficult to determine whether any differences in the measures arise because DTW dissimilarities are nonmetric or because DTW dissimilarities inherently compare different features.

Therefore, to evaluate the effect of the nonmetric measure on the analysis, we also compared DTW dissimilarities to a metric measure based on the DTW dissimilarities themselves. Specifically, we used classical (Torgerson's) multidimensional scaling (MDS) to embed seizures in Euclidean space based on the DTW dissimilarities. Note that we used this same approach to allow cluster evaluation using the gap statistic (see Methods, "Seizure clustering and cluster evaluation"). As before, to most closely approximate the DTW dissimilarities matrix, the seizures were projected onto the maximum possible number of dimensions. New, metric distances can then be computed between the embedded seizures; we will refer to this metric approximation of DTW dissimilarities as "Euclidean dissimilarities." The MDS embedding attempts to retain the "distances" (here, DTW dissimilarities) between seizures; however, the Euclidean dissimilarities will not exactly match the DTW dissimilarities because we are forcing a metric space to approximate nonmetric distances. Crucially, the amount of difference between the DTW dissimilarities and Euclidean dissimilarities therefore reveals how much the DTW dissimilarities differ from a metric distance.

Fig. S12.2 compares DTW dissimilarities to Euclidean dissimilarities. In patient 931, the DTW dissimilarity matrix (Fig. S12.2A) and Euclidean dissimilarity matrix (Fig. S12.2B) look very similar, although some of the lower DTW dissimilarities are increased in the Euclidean dissimilarity matrix (e.g., between seizures 1 and 2). Directly comparing the two measures across all seizure pairs in patient 931 (Fig. S12.2C) demonstrates that they are very similar: their Spearman correlation is 0.9991. As we observed when comparing the matrices, more similar seizures (i.e., seizure pairs with lower dissimilarities) have slightly higher Euclidean dissimilarities than DTW dissimilarities. This increase likely arises when a pair of seizures following a similar pathway (i.e., a pair of seizures with low dissimilarity) have different dissimilarities to partially similar seizures (e.g., see example above in, "Seizure dissimilarity is a nonmetric measure"). In the Euclidean embedding, two seizures cannot occupy the same space (and thus have a low distance) if they have different distances to other seizures. Thus, the dissimilarity between such seizures slightly increases in the embedding to maintain their relative relationships to other seizures.

In all patients, DTW dissimilarities and Euclidean dissimilarities have a high correlation (Fig. S12.2D); indeed, in the majority of patients, the correlation is above 0.95, indicating that the Euclidean approximation is very similar to the nonmetric measure. Further, the temporal correlation results are close regardless of which measure is used (Fig. S12.2E). The similarity between these results indicates that DTW dissimilarities are close to a metric measure, and its nonmetric features do not appear to affect downstream results.

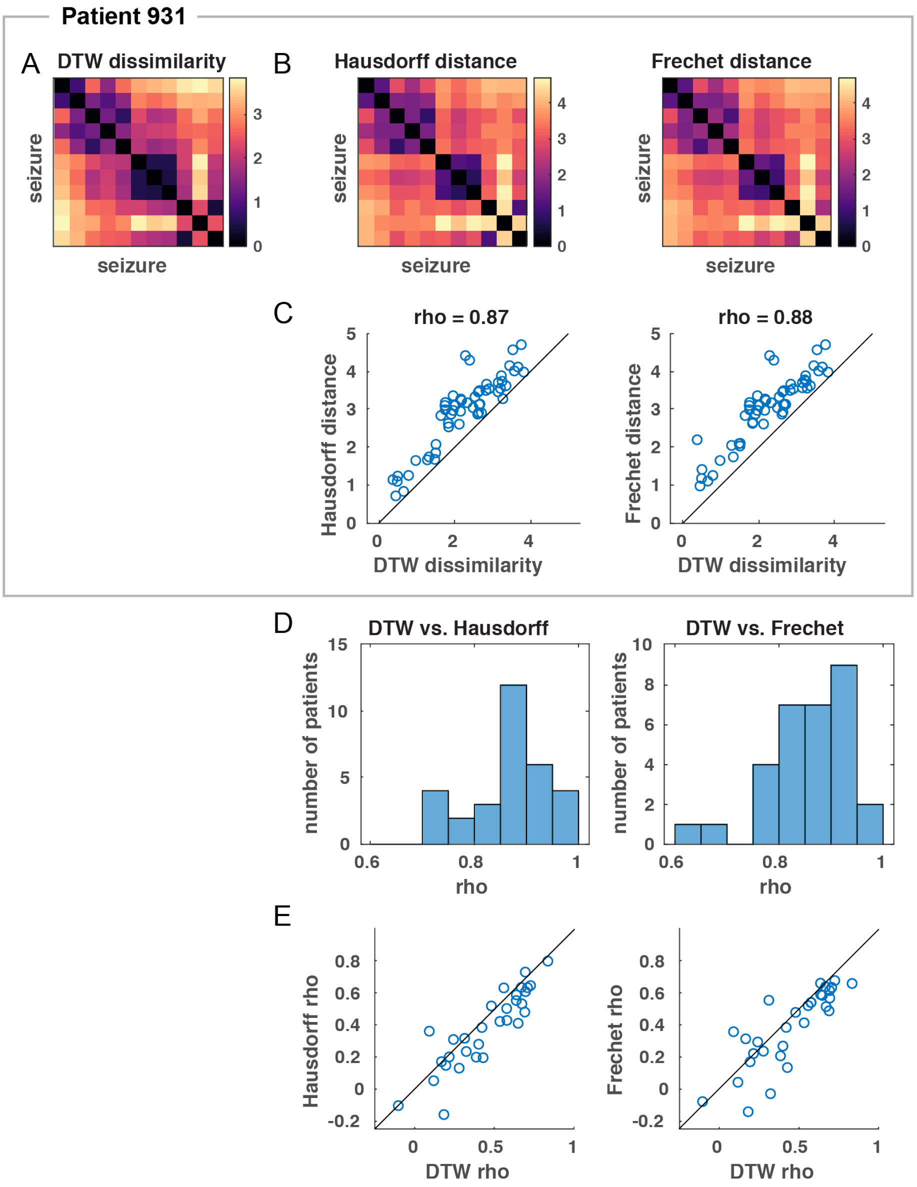

Fig. S12.1: Comparison of DTW dissimilarity measure to alternative dissimilarity measures. A) Seizure dissimilarities of patient 931, computed using our DTW measure of seizure dissimilarity. B) Seizure dissimilarities of patient 931, computed using two alternative measures: the Hausdorff distance (left) and Frechét distance (right). C) Scatter plots of the DTW seizure dissimilarities vs. the Hausdorff distances (left) and Frechét distances (right) of all seizure pairs in patient 931. The black line indicates the equivalence line. Spearman's correlation between each pair of measures is shown above each scatter plot. D) For each patient, Spearman's correlation was computed between the DTW dissimilarities and each of alternative measures for all pairs of seizures. These histograms show the distributions of those correlations across all patients. E) The correlation between each dissimilarity measure and temporal distances ("temporal correlation") was computed for each patient. These scatter plots show the temporal correlation from the DTW dissimilarity measure vs. the temporal correlations computed using each alternative measure of seizure dissimilarity. The black line indicates the equivalence line.

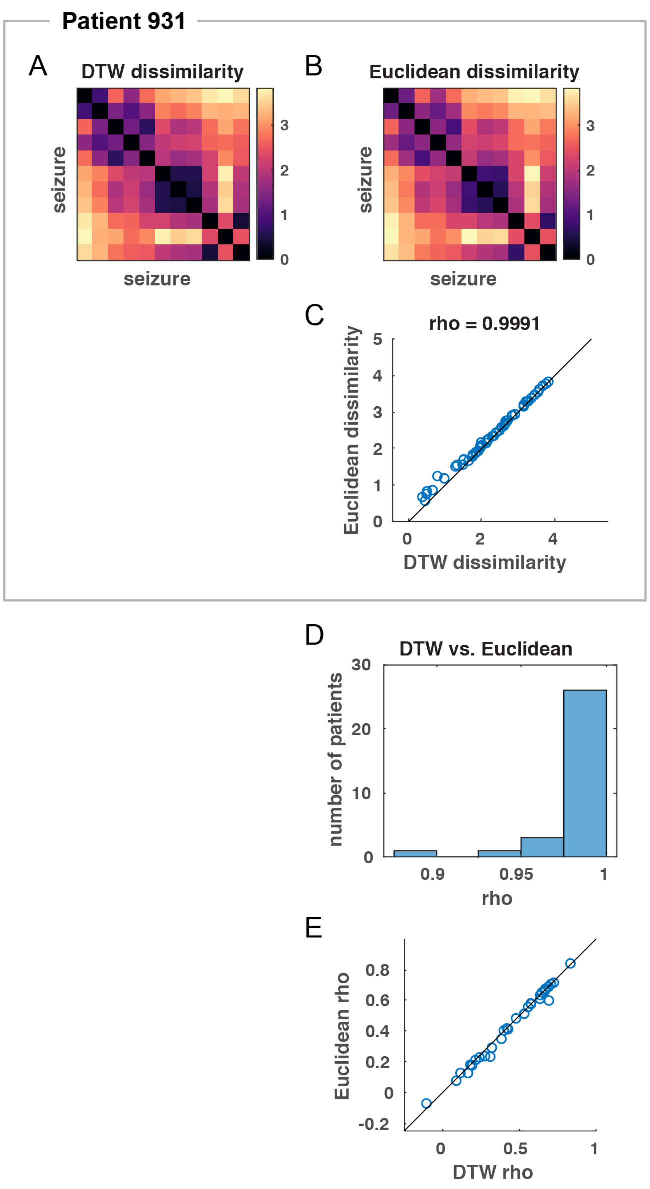

Fig. S12.2: Comparison of DTW dissimilarity measure to a metric approximation of the dissimilarities ("Euclidean dissimilarities"). A) Seizure dissimilarities of patient 931, computed using our DTW measure of seizure dissimilarity. B) Euclidean dissimilarities of patient 931, computed by embedding seizure dissimilarities in Euclidean space. Because the embedding is an approximation of the nonmetric dissimilarities, there will be some differences between the original dissimilarities and the approximation. C) Scatter plot of the DTW seizure dissimilarities vs. the Euclidean seizure dissimilarities of all seizure pairs in patient 931. The black line indicates the equivalence line. Spearman's correlation between the two measures is shown above the scatter plot. D) For each patient, Spearman's correlation was computed between the DTW dissimilarities and the Euclidean dissimilarities. This histogram show the distribution of correlations across all patients. E) The correlation between Euclidean dissimilarities and temporal distances ("temporal correlation") was computed for each patient. The scatter plot shows the temporal correlation from the DTW dissimilarity measure vs. the temporal correlation computed using the Euclidean dissimilarities. The black line indicates the equivalence line.

References

1. King-Stephens D, et al. (2015) Lateralization of mesial temporal lobe epilepsy with chronic ambulatory electrocorticography. *Epilepsia* 56(6):959–967.
2. Spencer SS, Spencer DD, Williamson PD, Mattson RH (1981) Ictal effects of anticonvulsant medication withdrawal in epileptic patients. *Epilepsia* 22:297–307.
3. Cook MJ, et al. (2016) Human focal seizures are characterized by populations of fixed duration and interval. *Epilepsia* 57(3):359–368.
4. Karoly PJ, et al. (2018) Seizure pathways : a model-based investigation. *PLoS Comput Biol* 14(10):e1006403.
5. Tibshirani R, Walther G, Hastie T (2001) Estimating the number of clusters in a data set via the gap statistic. *J R Stat Soc Ser B (Statistical Methodol* 63:411–423.
6. Bancaud J, et al. (1981) Proposal for revised clinical and electroencephalographic classification of epileptic seizures. *Epilepsia* 22:489–501.
7. Berg AT, et al. (2010) Revised terminology and concepts for organization of seizures and epilepsies: report of the ILAE Commission on Classification and Terminology, 2005-2009. *Epilepsia* 51(4):676–685.
8. Fisher RS, et al. (2017) Operational classification of seizure types by the International League Against Epilepsy: position paper of the ILAE Commission for Classification and Terminology. *Epilepsia* 58(4):522–530.
9. Engel JJ, Crandall PH (1983) Falsely localising ictal onsets with depth EEG telemetry during anticonvulsant withdrawal. *Epilepsia* 24:344–355.
10. Marciani MG, Gotman J (1986) Effects of drug withdrawal on location of seizure onset. *Epilepsia* 27(4):423–431.
11. Bardy AH (1992) Reduction of antiepileptic drug dosage for monitoring epileptic seizures. *Acta Neurol Scand* 86:466–469.
12. Meisel C, et al. (2015) Intrinsic excitability measures track antiepileptic drug action and uncover increasing/decreasing excitability over the wake/sleep cycle. *Proc Natl Acad Sci* 112(47):14694–14699.
13. Badawy RAB, Macdonell RAL, Berkovic SF, Newton MR, Jackson GD (2010) Predicting seizure control: cortical excitability and antiepileptic medication. *Ann Neurol* 67(1):64–73.
14. Wu S, et al. (2016) Stability-driven nonnegative matrix factorization to interpret spatial gene expression and build local gene networks. *Proc Natl Acad Sci* 113(16):4290–4295.
15. Burns SP, et al. (2014) Network dynamics of the brain and influence of the epileptic seizure onset zone. *Proc Natl Acad Sci* 111(49):E5321–E5330.
16. Khambhati AN, et al. (2017) Recurring functional interactions predict network architecture of interictal and ictal states in neocortical epilepsy. *eNeuro* 4(1):e0091–16.2017.
17. Khambhati AN, et al. (2015) Dynamic network drivers of seizure generation, propagation and termination in human neocortical epilepsy. *PLoS Comput Biol* 11(12):e1004608.
18. Kim J, He Y, Park H (2014) Algorithms for nonnegative matrix and tensor factorizations: a unified view based on block coordinate descent framework. *J Glob Optim* 58:285–319.
19. Kim J, Park H (2011) Fast nonnegative matrix factorization: an active-set-like method and comparisons. *SIAM J Sci Comput* 33(6):3261–3281.
20. Brunet JP, Tamayo P, Golub TR, Mesirov JP (2004) Metagenes and molecular pattern discovery using matrix factorization. *Proc Natl Acad Sci U S A* 101(12):4164–4169.
21. Kim H, Park H (2007) Sparse non-negative matrix factorizations via alternating non-negativity-constrained least squares for microarray data analysis. *Bioinformatics* 23(12):1495–1502.
22. Eiter T, Mannila H (1994) *Computing discrete Fréchet distance.*
